# Quantifying the relative impact of contact heterogeneity on MRSA transmission in ICUs - a modelling study

**DOI:** 10.1186/s12879-019-4738-0

**Published:** 2020-01-03

**Authors:** Hao Lei, Rachael M. Jones, Yuguo Li

**Affiliations:** 1 School of Public Health, Zhejiang University, Hangzhou, People’s Republic of China; 2 Zhejiang Institute of Research and Innovation, The University of Hong Kong, Lin An, Zhejiang People’s Republic of China; 3 Department of Family and Preventive Medicine, School of Medicine, University of Utah, Salt Lake City, UT USA; 4 Department of Mechanical Engineering, The University of Hong Kong, Pokfulam, Hong Kong, SAR People’s Republic of China

**Keywords:** MRSA, Mathematical model, Intensive-care unit, Contact heterogeneity, Surface hygiene

## Abstract

**Background:**

An efficient surface cleaning strategy would first target cleaning to surfaces that make large contributions to the risk of infections.

**Methods:**

In this study, we used data from the literature about methicillin-resistant *Staphylococcus aureus* (MRSA) and developed an ordinary differential equations based mathematical model to quantify the impact of contact heterogeneity on MRSA transmission in a hypothetical 6-bed intensive care unit (ICU). The susceptible patients are divided into two types, these who are cared by the same nurse as the MRSA infected patient (Type 1) and these who are not (Type 2).

**Results:**

The results showed that the mean MRSA concentration on three kinds of susceptible patient nearby surfaces was significantly linearly associated with the hand-touch frequency (*p* < 0.05). The noncompliance of daily cleaning on patient nearby high-touch surfaces (HTSs) had the most impact on MRSA transmission. If the HTSs were not cleaned, the MRSA exposure to Type 1 and 2 susceptible patients would increase 118.4% (standard deviation (SD): 33.0%) and 115.4% (SD: 30.5%) respectively. The communal surfaces (CSs) had the least impact, if CSs were not cleaned, the MRSA exposure to Type 1 susceptible patient would only increase 1.7% (SD: 1.3). The impact of clinical equipment (CE) differed largely for two types of susceptible patients. If the CE was not cleaned, the exposure to Type 1 patients would only increase 8.4% (SD: 3.0%), while for Type 2 patients, it can increase 70.4% (SD: 25.4%).

**Conclusions:**

This study provided a framework to study the pathogen concentration dynamics on environmental surfaces and quantitatively showed the importance of cleaning patient nearby HTSs on controlling the nosocomial infection transmission via contact route.

## Background

Healthcare-associated infections (HAIs) pose a significant health and economic burden globally [1]. HAIs transmission occurs through many processes, including the contact route, in which microorganisms move from surface to surface via contact [2]. An obvious intervention to limit the contact transmission of HAIs is to remove microorganisms from the surfaces and hands, such as through surface cleaning and hand hygiene. And these interventions have been shown to have impact on controlling HAIs [3–5].

An efficient strategy would first target cleaning to surfaces that make large contributions to the risk of HAIs. Studies on the improvement of room hygiene have focused on the high-touch surfaces (HTSs) [6], near patients surfaces [7, 8], or communal locations [9], which were associated with microbial contamination of HCW’s hands. The Centers for Disease Control and Prevention (CDC) recommends cleaning and disinfecting HTSs on a more frequent basis than low-touch surfaces (LTSs) in healthcare facilities [10]. However, there are few studies that quantitatively evaluate the relative effectiveness of cleaning and disinfecting different surfaces, including HTSs, near-patient surfaces and communal surfaces, on controlling HAIs via the contact route [11].

Mathematical models have been successfully applied to evaluate the transmission of HAIs [12, 13]. However, most of these studies assumed homogeneous mixing of surfaces, which cannot reproduce the intricacies of surface contact patterns in hospitals. In this study, similarly to previous work [14], we approach this problem using a mathematical model describing the concentration dynamics of methicillin-resistant *Staphylococcus aureus* (MRSA) on environmental surfaces and hands, but now consider a 6-bed intensive care unit (ICU) with a more realistic system of surfaces contacted by patients and healthcare workers (HCWs). We used this model to investigate how contact heterogeneity on hospital environmental surfaces may influence the exposure of susceptible patients to MRSA via the contact route, given non-compliance with surface cleaning.

## Methods

### Governing equations

Mathematical modeling has been used for many years to simulate MRSA transmission [15], including continuous-time dynamical model [16, 17] and stochastically discrete-time Monte Carlo model [18] or Markov model [19]. The continuous-time dynamical model described an average behavior of the discrete-time behaviors, and could provide a good approximation to the stochastic model in a large scale. Given the settings we considered in this study, a continuous-time ordinary differential equations (ODEs) based model, developed by Plipat et al. [20] and Lei et al. [14], was used to simulate the MRSA concentration dynamics in different compartments, representing environmental surfaces, patients’ hands, patients’ nares, HCWs’ hands and HCWs’ nares. A brief description is provided here, and more details are included in Additional file 1: Part A.

The model includes *N* compartments in total, where the MRSA concentration in compartments *i* and *j* at time *t* are *C*_*i*_(*t*) and *C*_*j*_(*t*) (CFU/cm^2^) respectively. Compartments *i* and *j* have area A_*i*_ and A_*j*_ (cm^2^), respectively. Assuming that after each contact, MRSA in compartments *i* and *j* would be uniformly distributed across the area. Also assuming there is continuous emission of MRSA into compartment *i* at rate *e*_*i*_ (CFU/ cm^2^/h), and two continuous removal processes: loss of infectivity (decay) at rate *d*_*i*_ (/h), and removal due to cleaning at rate *α*_*i*_ (/h).

Contact between compartments *i* and *j* will result in pathogen exchange between the compartments as follows. Denoting the pathogen concentration in compartments *i* to *j* at time *t* before contact as *C*_*i*_*(t)* and *C*_*j*_*(t)* (CFU/cm^2^), respectively. A contact between compartments *i* and *j* occurs in one time step, *Δt*, and after contact *C*_*i*_*(t + Δt)* − *C*_*i*_*(t) =* (*C*_*j*_*(t)*A_*ij*_*τ*_*ji*_ − *C*_*i*_*(t)*A_*ij*_*τ*_*ij*_)*/*A_*i*_, where *τ*_*ij*_ is the MRSA transfer efficiency from compartments *i* to *j* during contact (0 ≤ *τ*_*ij*_ *≤ 1)* and A_*ij*_ is the contact area between the two surfaces during contact. Similarly, *C*_*j*_*(t + Δt)* − *C*_*j*_*(t) =* (*C*_*i*_*(t)*A_*ij*_*τ*_*ij*_ − *C*_*j*_*(t)*A_*ij*_*τ*_*ji*_)*/*A_*j*_. Reflecting the conservation of mass, *C*_*i*_*(t) + C*_*j*_*(t) = C*_*i*_*(t + Δt) + C*_*j*_*(t + Δt)* if no loss processes occur. A matrix ***θ*** = (*θ*_*ij*_)_*N* × *N*_ is built to describe MRSA transfer rates (cm^2^/h) between the *N* compartments in the modeled ICU, where *θ*_*ij*_ is the product of: 1) the contact rate between compartment *i* and *j, β*_*ij*_ (/h), 2) the contact area between compartment *i* and *j* during contact, A_*ij*_ (cm^2^), and 3) the transfer efficiency from compartment *i* to *j, τ*_*ij*_. Thus *θ*_*ij*_ = *β*_*ij*_A_*ij*_*τ*_*ij*_.

The change in MRSA concentration in compartment *i* at time *t* is:

$$ \frac{d}{dt}{C}_i(t)={e}_i-{d}_i{C}_i(t)-{\alpha}_i{C}_i(t)+\frac{\left[\sum \limits_{j=1}^N{\theta}_{ji}{C}_j(t)-\sum \limits_{j=1}^N{\theta}_{ij}{C}_i(t)\right]}{A_i},i=1,2,\dots, N $$

### The ICU

**Fig. 1:**
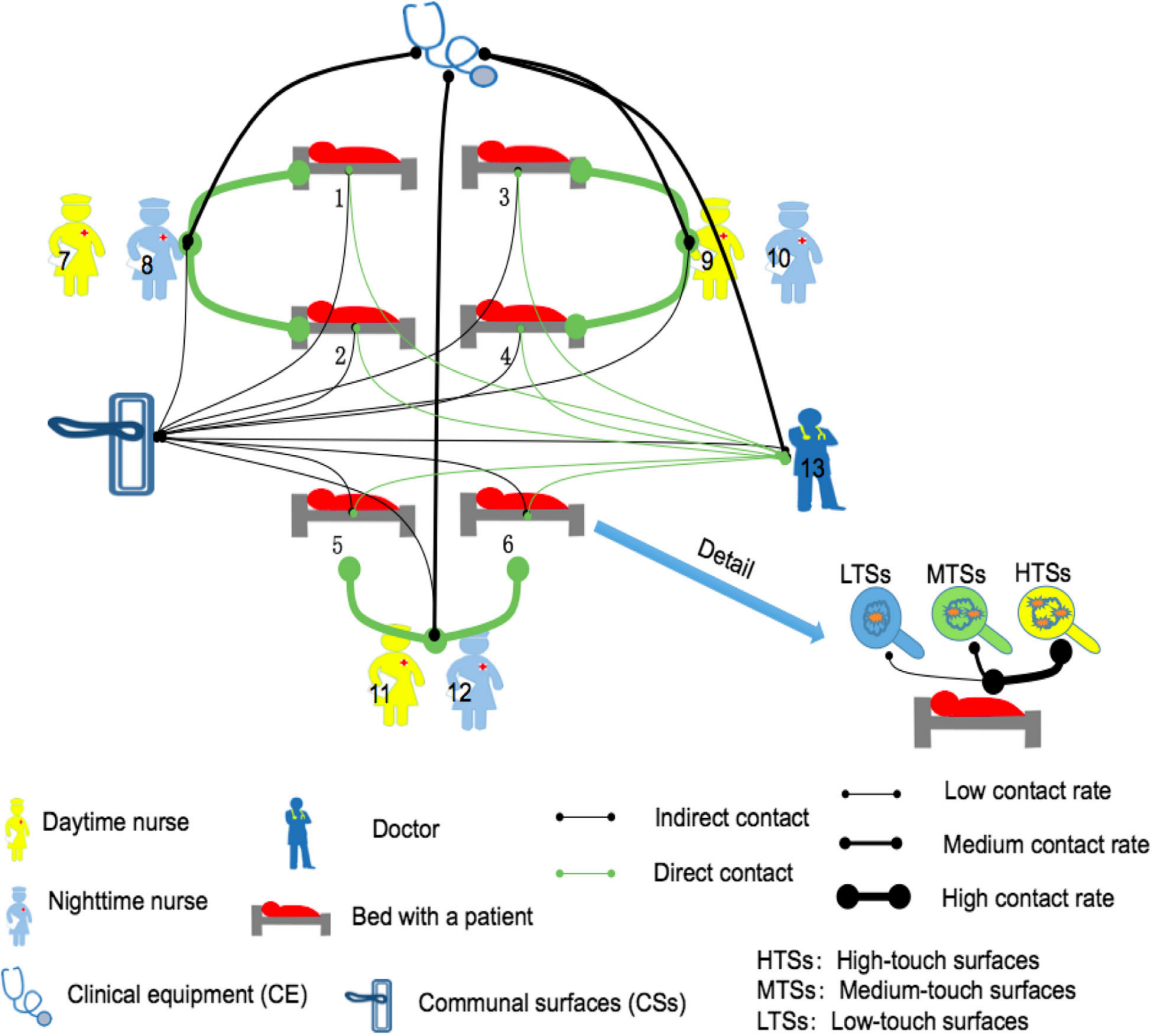
The contact network in the modeled 6-bed ICU

Since patients in an ICU are at increased risk of acquiring HAIs compared with those on a general ward [21], we modeled MRSA transmission via the contact route in a hypothetical 6-bed ICU. We considered that the ICU was staffed by three daytime and three nighttime nurses, each dedicated to a pair of beds, and one physician. Without loss of generality, it was assumed patient 1 is colonized with MRSA, and all other people are susceptible to MRSA colonization or infection (Fig. 1).

We classified the numerous surfaces in the ICU into three groups: communal surfaces, clinical equipment and surfaces near patients. Communal surfaces (CSs) are those touched by all patients and/or HCWs in the ICU, including: room dividers (curtains), door handles, etc. Clinical equipment (CE) are those items typically touched only by the HCWs. Near-patient surfaces (NPSs) are typically touched only by each patient and the HCWs that care for that patient, and include: the bedrail, bed surfaces and overbed table, etc. The NPSs were subdivided based on the rate of contacts by HCWs reported by Huslage et al. [6] into HTSs, medium-touch surfaces (MTSs) and low-touch surfaces (LTSs). As a result, for each patient, there were three kinds of NPSs (HTSs, MTSs, and LTSs). In total, 20 environmental surface compartments were included in the model: 1 CSs, 1 CE, 6 HTSs, 6 MTSs and 6 LTSs. Since our primary interest is the contribution of each type of surface to MRSA transmission, we did not further subdivide the five surface types. The assumed touched area of CSs, CE, and near-patient HTSs, MTSs and LTSs are 2000, 3000, 2100, 1800, 2600 cm^2^ respectively (Additional file 1: Part B, Table B.1). Since these values are relatively similar, we assumed each surface type had a 2300 cm^2^ area, the average of the areas of the five surface types. Each of the 13 individuals in the 6-bed ICU were represented in the model by two compartments: the hands and the exposure site – the nares, which is a site frequently colonized by MRSA. In total, the model included 46 compartments representing places where MRSA might be.

### HCW schedule and allocation

In an ICU, the nurse to patient ratio is often 1:1 to 1:2 [22]. We considered that nurses worked a daytime shift (9:00 to 21:00) and nighttime shift (21:00 to 9:00 in the next day), and that a 1:2 nurse to patient ratio was maintained. The physician to patient ratio was assumed to be 1:6 [23]. Nurses and the physician spent about 30% of their work shifts in direct patient care, and we assumed nurses and the physician visited patients 4 times and 1 time per work shift respectively [23, 24]. Thus, each nurse spent 27 min with each patient per visit (0.3 × 12 × 60/(2 × 4) = 27 min) and the physician spent 27 min with each patient per visit (0.3 × 9 × 60/6 = 27 min), which is close to the time estimated by Temime et al. [23]. For nurses, since each nurse visits each patient 4 times per work shift, it is assumed that the nurse visits patients once per 3 h and visits the two patients in the first 54 min (0.9 h) of the 3-h period, without considering the order of the visits, and spends the remaining time at the nurses’ station. For the physician, it was assumed that he/she visits 6 patients consecutively, starting at 9:00, spending 27 min with each patient, also without considering the visit order. For the remainder of the work shift, the physician is not present in the ICU.

The observation studies by Huslage et al. [6], McArdle et al. [25] and Cheng et al. [26] were used to estimate the nurse-to-patient and physician-to-patient contact rates during healthcare visits, and the contact rates for nurses, the physician and patients with environmental surfaces (Additional file 1: Part C).

### Model intervention

There is no set frequency of whole-room surface cleaning and disinfection in hospitals [27], but it is likely to occur daily in room with patients with an infectious disease [20]. To reflect this condition, the baseline surface cleaning in this study was daily cleaning on all surfaces at 8:00 am, just before the daytime nurses began to work at 9:00 am. We assumed that surface cleaning and disinfection efficacy was 91% [28]. Regarding compliance of hospital cleaning and disinfection, we assumed cleaning was either fully compliant (91% of the MRSA removed by cleaning and disinfection) or non-compliant (not cleaned and not disinfected). This is obviously not the real situation, but we felt it was pertinent as a case study.

In the ICU, it’s required that HCWs should wash and clean hands before and after contact with each patient, since each HCW spent 27 min with each patient per visit, so the required hand cleaning frequency was about 4.4 times per hour during healthcare visit. However the compliance with hand hygiene is poor [29, 30]. We assumed HCWs would clean their hands after 42% of healthcare visits with patients, which is the average hand hygiene compliance rate observed in 12 studies conducted in ICUs (Additional file 1: Part A. Table C.5). Then the HCWs hand cleaning frequency was 4.4 × 42% = 1.8 per hour. The hand hygiene efficacy at removing pathogens is 90% [23]. In order to represent the discrete nurse hand hygiene process in the continuous governing ODEs, the following translation is made. After each hand hygiene, only a fraction (1–90%) of MRSA remains on hands. Hand hygiene occurs 1.8 time per hour, and the time-average rate of pathogen removal due to hand hygiene is denoted by α_h_ (/h). Then on average, there is 1–90% = ${e}^{-{\upalpha}_h/1.8}$ and α_h_ = −log(1–90%)*1.8 = 4.1/h. It was assumed that patients do not wash their hands during the simulation time.

### Pathogen emission, inactivation and transmission

Methicillin-resistant *Staphylococcus aureus* (MRSA) is a common nosocomial pathogen, and was used as an example to study disease transmission via contact route. Data regarding the MRSA emission rate and the inactivation rate on surfaces are listed in Additional file 1: Part C. In this study, the transmission route of interest is the contact route, involving direct contact between HCWs and patients’ bodies and indirect contact by HCWs and patients with MRSA-contaminated surfaces (fomites); direct patient-to-patient transmission was ignored.

### Initial conditions

The initial MRSA concentration in 45 of 46 compartments was set to zero, with the exception of the exposure site (nares) of the index patient, which was assumed to have a constant MRSA concentration of 250 CFU/cm^2^ [20].

### Evaluation of surface cleaning effectiveness

The main aim of surface cleaning is to prevent the transmission of the MRSA from an index patient to susceptible patients. As a result, the mean pathogen concentration at the exposure site of susceptible patients over 1 day (defined as 8 am to 7:59 am of the next day) was used as the indicator of the impact of noncompliance with surface cleaning. The model was simulated for several consecutive days until the mean MRSA concentration at the exposure site of susceptible patients is similar from day to day. Since the baseline surface cleaning in this study was daily cleaning on all surfaces, then the effect of noncompliance with cleaning on a type of surface would be expected to increase the MRSA concentration at patient’s exposure sites.

### Simulation

For each cleaning condition, the model was implemented to simulate the change in MRSA concentrations over several consecutive days with 100 replications to reflect variance in parameter distributions. The mean and standard deviation (SD) of MRSA concentrations at the patient exposure sites and other surfaces estimated in the 100 model replications were presented.

## Results

The mean MRSA concentrations at the exposure site of susceptible patient 2 on 7 consecutive days after the index patient was admitted to the ICU are 0.17 (SD: 0.058), 0.22 (SD: 0.080), 0.23 (SD: 0.084), 0.23 (SD: 0.084), 0.23 (SD: 0.084), 0.23 (SD: 0.084), and 0.23 (SD: 0.084) CFU/cm^2^, respectively. The mean MRSA concentrations at the exposure site of patients 3–6 on 7 consecutive days are 0.020 (SD: 0.0087), 0.027 (SD: 0.012), 0.028 (SD: 0.013), 0.028 (SD: 0.013), 0.028 (SD: 0.013), 0.028 (SD: 0.013) and 0.028 (SD: 0.013) CFU/cm^2^, respectively. As we can see from the above results, the mean MRSA concentration on the nares of susceptible patients is stable between after days 3, so the MRSA concentration dynamics in the third day was used for analysis.

### MRSA concentration dynamics

**Fig. 2:**
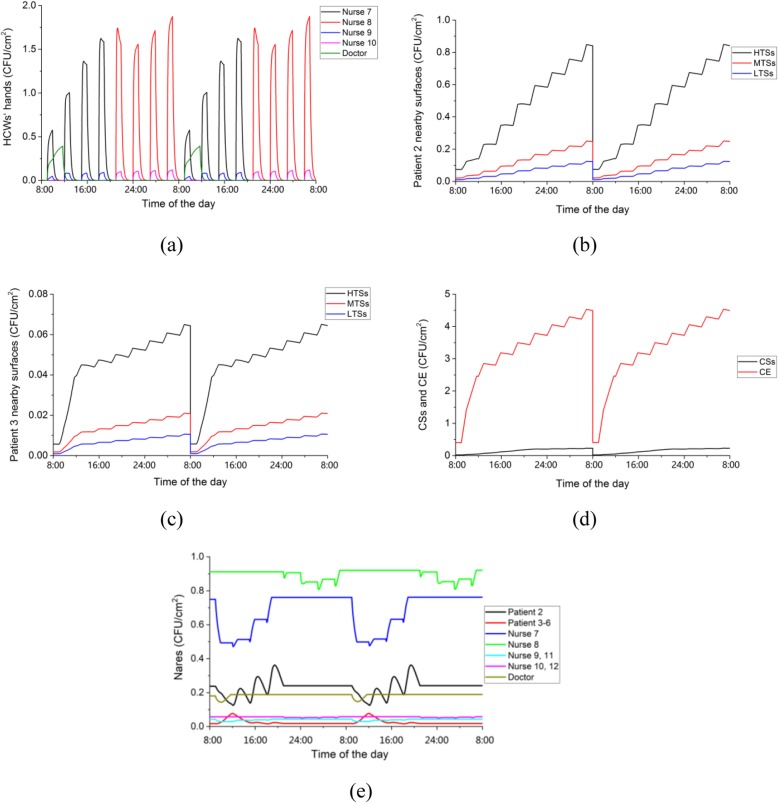
MRSA concentrations (CFU/cm^2^) dynamics on the third and fourth day after introduction of the index patient: **a** on hands of HCWs, **b** on bed nearby surfaces of patient 2, **c** on bed nearby surfaces of patient 3, 4, 5, 6, **d** on communal surfaces (CSs) and clinical equipment (CE), **e** on nares. Note difference in scales on y-axis

Figure 2 shows the temporal change of the mean MRSA concentrations on hands of HCWs and environmental surfaces during the third and fourth day after admission of the colonized index patient to the ICU. The MRSA concentration on HCWs’ hands increases during visits with patients, but decreases rapidly due to hand hygiene and relatively high inactivation rate of MRSA on hands (0.57 /h). Though the MRSA concentrations on environmental surfaces increased slowly compared with that on hands, MRSA will accumulate to a high level if surface cleaning is not performed, due to the relatively low rate of MRSA on environmental surfaces (0.0082 /h). This shows the importance of surface cleaning on control the influence of fomites on MRSA transmission.

The MRSA concentrations on surfaces near patient 2 is about 10-times greater than the concentrations on surfaces near patient 3. This is mainly because patient 2 is closer to the index patient than patient 3 in the contact network. That is, MRSA transmission to surfaces near patient 2 requires only two steps: 1) from the index patient (patient 1) to a nurse’s hands (nurse 7 or 8 in Fig. 1), and 2) from the nurse’s hands to surfaces near patient 2. Transmission to surfaces near patient 3 requires four steps: 1) from the index patient (patient 1) to a nurse’s hands (nurse 7 and/or 8), 2) from the nurse’s hands to CSs and/or CE, 3) from contaminated CSs and/or CE to a nurse’s hands (nurse 9 and/ or 10), and 4) from the nurse’s hands to surfaces near patient 3. The transfer efficiencies of the two additional steps were 0.23 and 0.28, so the MRSA concentration on surfaces near patient 3 would be about 6.5% (0.23 × 0.28 = 0.065) of the concentration on surfaces near patient 2. Of course, the steps for MRSA transmission from patient 1 to patients 2 and 3 via the physician are the same, so the ratio of MRSA concentrations on surfaces near patient 2 and patient 3 would be a little higher than 6.5%.

### Association between contact frequency and MRSA contamination

**Fig. 3:**
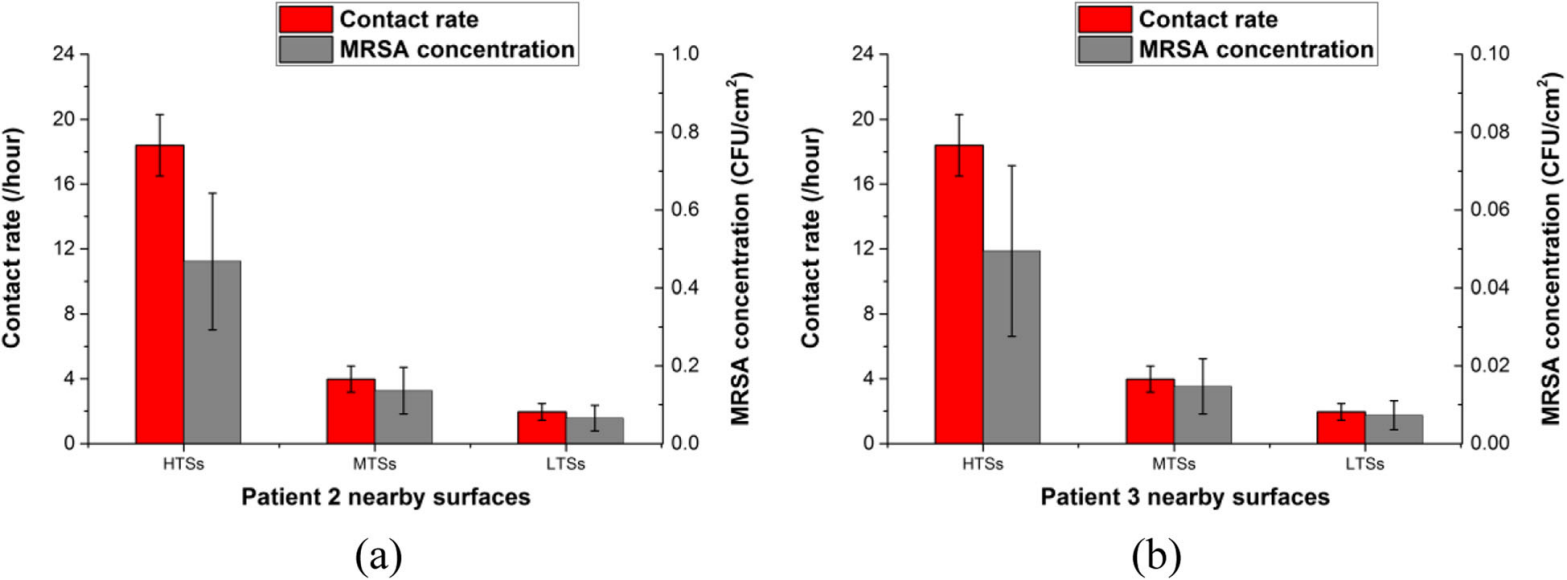
Hand-touch frequency and mean MRSA concentration for three kinds of patient nearby surfaces, **a** for patient 2, **b** for patient 3. Error bar represents the standard deviation from 100 replicate simulations

We tested the association between the contact frequency and MRSA contamination for the three kinds of near-patient surfaces: HTSs, MTSs and LTSs. The mean contact rates on HTSs, MTSs and LTSs were 18.4 (SD: 1,9), 3.98 (SD: 0.81) and 1.95 (SD: 0.52) per hour, respectively. There was a significant positive association between contact frequency and mean MRSA concentration on the three near-patient surfaces, yielding a Pearson’s ρ = 1 for surfaces near patient 2 (*p* = 0.033) and patient 3 (*p* = 0.032) (Fig. 3).

### Impact of the surface cleaning non-compliance on MRSA transmission

**Fig. 4:**
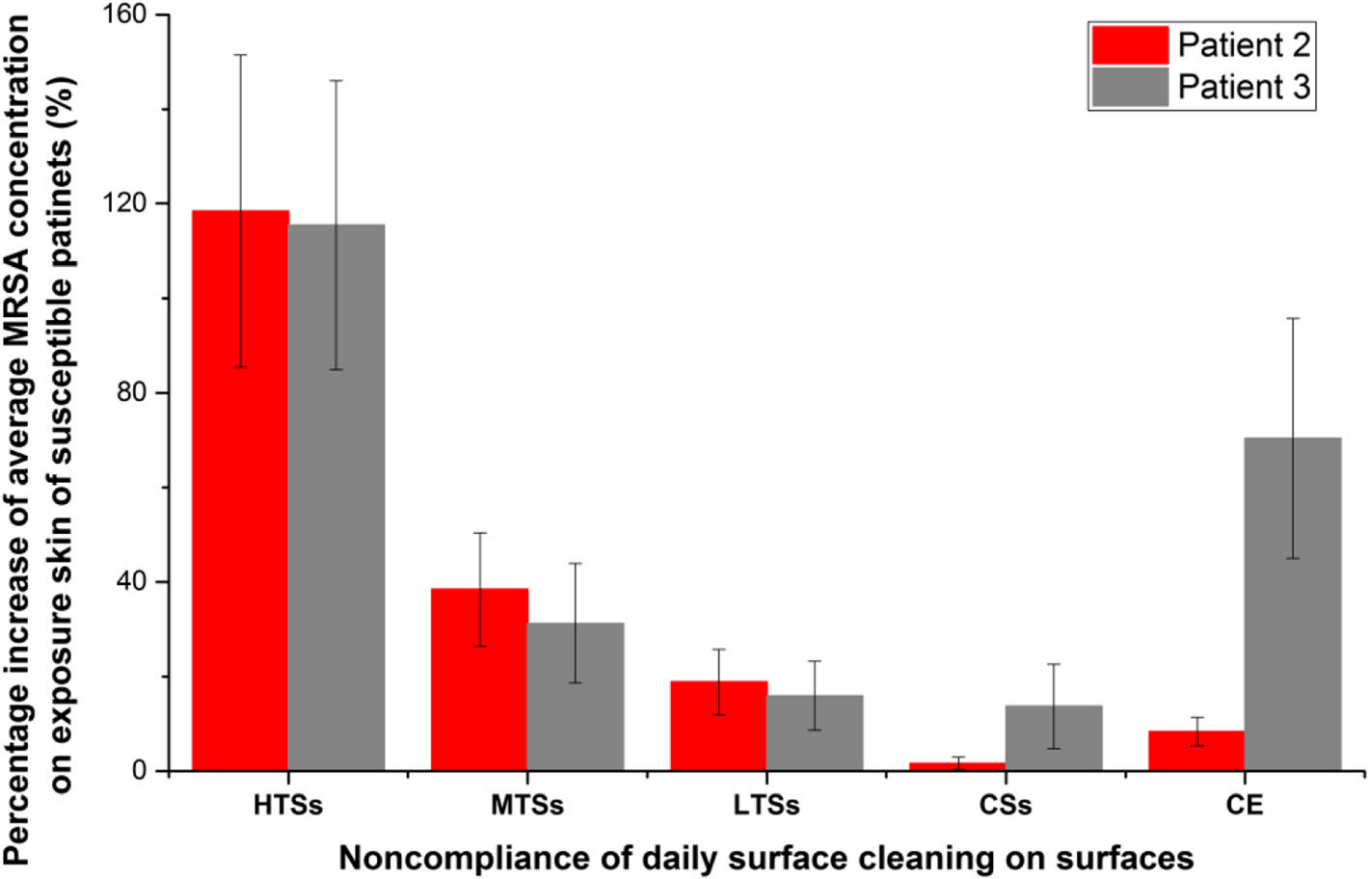
Percentage increase of mean MRSA concentration at the exposure site of susceptible patients on day 3 after admission of the index patient given non-compliance with cleaning of HTSs, MTSs, LTSs, CSs and CE respectively. HTSs: high-touch surfaces, MTSs: medium-touch surfaces, LTSs: low-touch surfaces, CSs: communal surfaces, CE: clinical equipment. Error bar represents the standard deviation

Figure 4 shows the predicted percentage increase of mean pathogen concentration on the exposure sites of patients 2 and 3 three days after admission of the index patient, given non-compliance with cleaning HTSs, MTSs, LTSs CSs, or CE. For both patients, cleaning non-compliance on HTSs has the most impact, leading to an 118% (SD: 33) and 115% (SD: 30) increase in MRSA concentration at the exposure site of patient 2 and 3 respectively. Non-compliance with cleaning CSs has the smallest impact. Non-compliance with cleaning CE increases the exposure of patient 3 more than the exposure of patient 2 (70% V.S. 8% increase). This is because different nurses care for patients 1 and 3, meaning that nurses’ contacts with CE mediates the transmission pathway.

## Discussion

It is widely accepted that environmental surfaces, clinical equipment, and the hands of HCWs contribute to the contact transmission of HAIs [6]. And it has been well documented that many nosocomial pathogens such as MRSA and vancomycin-resistant enterococci (VRE), can survive on hospital surfaces for days, up to a year [31]. These pathogens are always readily transmitted from environmental surfaces to HCWs’ and patients’ hands [7]. Surface cleaning has at least two impacts. First, it reduces the surface pathogen contamination level, which means that fewer pathogen is present to be transmitted to the susceptible patients during contact with hospital surfaces. Secondly, it reduces the HCW’s hand contamination level, and then controls the pathogen transmission from HCW-to-patient. A randomized crossover field study in an ICU found that enhanced cleaning in the near-patient environment significantly reduced the number of MRSA isolates in the environment and on the hands of the HCWs [32].

There is heterogeneity in the frequency with which HCWs and patients contact environmental surfaces, and it makes sense to clean and disinfect surfaces that are frequently touched, rather than clean and disinfect all possible surfaces equally, as touching transfers pathogens to and from hands [6]. In this study, we showed that MRSA concentrations on surfaces near MRSA-susceptible patients were positively associated with the frequency of hand contacts, partly due to that our model was a linear ODEs model. This trend was also observed in a field study in a 10-bed adult ICU, in which the bioburden on five near-patient surfaces was linearly associated with the frequency of hand contacts [21]. Theoretical work in network modeling has also shown that disease spread more easily among highly connected individuals [33]. According to the modeling study, the absolute MRSA concentration on the nares of susceptible patients was low, particularly compared to the index patient, this is because it was assumed that the MRSA on each compartment is uniformly distributed in the model. The real situation could be that the MRSA would be more concentrated on a small part of the compartment, then more pathogen would be transferred to susceptible individuals compared with the simulation results.

We quantitatively showed that HTSs near the patient play the most important role in the contact transmission to susceptible patients (Fig. 4). Failure to clean HTSs near the patients on a daily basis was estimated to increase the MRSA transmitted to susceptible patients by approximately 120%. Similarly, in an office building, daily disinfection of highly-touched surfaces had the greatest impact on controlling the MS2 phage concentration on workers’ hands, yielding an average reduction in phase concentrations of 41.7% [34], which means that noncompliance of daily disinfection of high-touch surface would lead to an 140% ([1–41.7%]/41.7% = 140%) increase of phage transmitted to workers. It is already known that traditional communal surfaces such as toilets, floor and sinks tend to attract high rates of cleaning [35], while near-patient hand-touch sites such as bed rail, overbed table and bedside table, are not always well cleaned [36, 37]. The responsibility for cleaning near-patient hand-touch sites has been found to fall to nurses, who are typically very busy and may be unable to perform this task consistently [38]. Two studies in ICUs have demonstrated an increased risk of HAIs following periods of inadequate nurse staffing [39, 40].

Our study is prone to a number of limitations. Firstly, this study used MRSA as an example of contract-transmissible pathogens relevant to healthcare settings, and used MRSA-specific values for model parameters and assumptions, which may be generalizable to other pathogens. For example, the assumption that no MRSA was released directly onto communal surfaces may be reasonable for some respiratory infections, such as influenza and SARS, but is not reasonable for norovirus, which is released into communal areas (washrooms) in diarrhea and vomit. In this context, cleaning of communal washroom surfaces should be very important in preventing the norovirus transmission via the contact route. The assumption about no emission to communal surfaces, may therefore overemphasize the importance of near-patient sites for some nosocomial pathogens transmitted via the contact route. In addition, this study also did not consider other source of pathogens, such as mobile contamination by healthcare workers. Second, in determining the pathogen transfer efficiency from one type of surface to hand and the reverse direction, the surfaces are all thought to be non-porous surfaces. This is due to most surfaces in UIC are non-porous surfaces. However, there still are some porous surfaces in hospital, such as bed surfaces and clothes. The relative low pathogen transfer efficiency from porous surface to hand and relative high pathogen transfer efficiency from hand to porous surface may lead much pathogen harbour on the bed surfaces, then the cleaning of HTSs (bed surface is one HTS in our model) in reality may be more important than we obtained in this study. Finally, the ODEs model we used in this study could provide a good approximation of the average behaviors in large scale settings, but may not be applicable in all situations.

## Conclusions

In summary, in this study, we built an ODEs based mathematical model to study the pathogen concentration dynamics and explored the relative importance of different surfaces with different contact frequencies on MRSA transmission in a 6-bed ICU. The pathogen concentration on patient nearby high-touch surfaces were found to be linearly associated with the contact frequencies, and therefore, the high-touch surfaces should be cleaned more carefully during the daily surface hygiene. The findings of this study contribute to the development of targeted surface cleaning strategies. The model and associated approaches could also be applied to other pathogens in indoor environments.

## Additional file

**Additional file 1.** Appendix.

## Data Availability

The datasets and computer program used in the study are available from the corresponding author on reasonable request.

## Abbreviations

CDC: Centers for Disease Control and Prevention
CE: Clinical equipment
CSs: Communal surfaces
HAIs: Healthcare-associated infections
HCWs: Healthcare workers
HTSs: High-touch surfaces
ICU: Intensive care unit
LTSs: Low-touch surfaces
MRSA: Methicillin-resistant *Staphylococcus aureus*
MTSs: Medium-touch surfaces
NPSs: Near-patient surfaces
SD: Standard deviation
