# Supplementary material for: Quantifying the relative impact of contact heterogeneity on MRSA transmission in ICUs - a modelling study
Source: BMC Infect Dis. 2020 Jan 3;20:6. doi: 10.1186/s12879-019-4738-0 (PMC6942315; doi:10.1186/s12879-019-4738-0)
Supplement: Supplementary file 1 — Additional file 1. Appendix. [file 12879_2019_4738_MOESM1_ESM.docx]

**Additional file**

**Part A. Contact network and governing equations in the modeled ICU.**

The IDs of the 46 compartments are arranged as follow: ID 1-5 refer to the hands, exposure skin, HTSs, MTSs and LTSs near patient 1 respectively. Then ID 6-10, 11-15, 16-20, 21-25, and 26-30 refer these of patient 2, 3, 4, 5, 6. ID 31-32, 33-34, 35-36, 37-38, 39-40, 41-42, 43-44 refer to the hands and exposure skin of the HCW 7, 8, 9, 10, 11, 12, 13 respectively. ID 45 refers to the communal surfaces (CSs) and ID 46 refers to the clinical equipment.

**Governing equations**

A matrix $\theta$=($\theta_{ij}$)_46×46_ built to describe pathogen transfer rates (cm^2^/h) between the 46 compartments (contact network) in the modeled ICU, where $\theta_{ij}$ is the pathogen transfer rate from compartment *i* and *j* (cm^2^/h), which is the product of contact rate between compartment *i* and *j,* $\beta_{ij}$ (/h), the contact area between compartment *i* and *j* during contact, A*_ij_* (cm^2^), and the transfer efficiency from compartment *i* to *j,* *τ_ij_*. Thus $\theta_{ij}=\beta_{ij}$A*_ij_τ_ij_*.

The pathogen concentration on 46 compartments is denoted by a matrix $C(t)$=($C_{i}(t)$)_46×1_. The pathogen emission rate on 46 compartments is denoted by a matrix $E$=($e_{i}$)_46×1_. And the pathogen inactivation rate on 46 compartments is denoted by a matrix $D$=($d_{i}$)_46×1_. Then there is

$\frac{d}{dt}C_{i}(t)=e_{i}-d_{i}C_{i}(t)+(\sum_{j=1}^{46} \theta_{ji}C_{j}(t)-\sum_{j=1}^{46} \theta_{ij}C_{i}(t))/A_{i}$, *i*=1, 2,…, 46.

When model intervention occurs, such as hand hygiene (for HCWs) and surface hygiene, it’s assumed that there is relative pathogen concentration changing in a instant at the time when the intervention occurs.

**Pathogen emission matrix *E*:**

$e_{1}=e_{3}=e_{4}=e_{5}=0.6$ (Index patient hands and his/her nearby surfaces), $e_{46}=0.6(\frac{W_{dn}}{2}+\frac{W_{nn}}{2}+\frac{W_{d}}{6})$ (emission on the clinical equipment when HCWs care the index patient), for others, $e_{i}=0$

Where $W_{dn}(t)$, $W_{nn}(t)$and $W_{d}(t)$ are the time indicator function for daytime, nighttime nurse and doctor (physician) working time respectively.

$$W_{dn}\left( t \right)=\left\{ \begin{aligned} 1, p+24n<t<p+0.9+24n, p\in\left( 9,12,15,18 \right),n\in Z^{+}. \\ 0, \mathrm{otherwise} \end{aligned} \right.$$

$$W_{nn}(t)=\left\{ \begin{aligned} 1, p+24n<t<p+0.9+24n, p\in\left( 21,24,3,6 \right),n\in Z^{+}. \\ 0, \mathrm{otherwise} \end{aligned} \right.$$

$$W_{d}(t)=\left\{ \begin{aligned} 1, 9+24n<t<9+2.7+24n, n\in Z^{+}. \\ 0, \mathrm{otherwise} \end{aligned} \right.$$

**Pathogen death matrix *D*:**

$d_{1}=d_{6}=d_{11}=d_{16}=d_{21}=d_{26}=$0.57 (hands), $d_{2}=d_{7}=d_{12}=d_{17}=d_{22}=d_{27}=$0 (exposure skin), for others (environmental surfaces), $d_{i}=0.0082$.

**Contact area matrix *A*:**

In this model, the hand contact area with all environmental surfaces is assumed to be 42 cm^2^. The hand contact area with all exposure skin is assumed to be 2 cm^2^. The hand contact area during hand-to-hand contact is assumed to be a 42 cm^2^.

Contact rate matrix ($\beta={{(\beta}_{ij})}_{46\times46}$)

$$\beta=$$

$$\begin{matrix} \\ \begin{matrix} 1 \\ 2 \\ \begin{matrix} 3 \\ 4 \\ \begin{matrix} 5 \\ 6 \\ \begin{matrix} 7 \\ 8 \\ \begin{matrix} 9 \\ 10 \\ \begin{matrix} 11 \\ 12 \\ \begin{matrix} 13 \\ 14 \\ \begin{matrix} 15 \\ 16 \\ \begin{matrix} 17 \\ 18 \\ \begin{matrix} 19 \\ 20 \\ \begin{matrix} 21 \\ 22 \\ \begin{matrix} 23 \\ 24 \\ \begin{matrix} 25 \\ 26 \\ \begin{matrix} 27 \\ 28 \\ \begin{matrix} 29 \\ 30 \\ \begin{matrix} 31 \\ 32 \\ \begin{matrix} 33 \\ 34 \\ \begin{matrix} 35 \\ 36 \\ \begin{matrix} 37 \\ 38 \\ \begin{matrix} 39 \\ 40 \\ \begin{matrix} 41 \\ 42 \\ \begin{matrix} 43 \\ 44 \\ \begin{matrix} \begin{matrix} 45 \\ 46 \end{matrix} \end{matrix} \end{matrix} \end{matrix} \end{matrix} \end{matrix} \end{matrix} \end{matrix} \end{matrix} \end{matrix} \end{matrix} \end{matrix} \end{matrix} \end{matrix} \end{matrix} \end{matrix} \end{matrix} \end{matrix} \end{matrix} \end{matrix} \end{matrix} \end{matrix} \end{matrix} \end{matrix} \end{matrix}\left[ \begin{matrix} 1 \\ \begin{matrix} 0 \\ 5.0 \\ \begin{matrix} 4.6 \\ 1.0 \\ \begin{matrix} 0.5 \\ 0 \\ \begin{matrix} 0 \\ 0 \\ \begin{matrix} 0 \\ 0 \\ \begin{matrix} 0 \\ 0 \\ \begin{matrix} 0 \\ 0 \\ \begin{matrix} 0 \\ 0 \\ \begin{matrix} 0 \\ 0 \\ \begin{matrix} 0 \\ 0 \\ \begin{matrix} 0 \\ 0 \\ \begin{matrix} 0 \\ 0 \\ \begin{matrix} 0 \\ 0 \\ \begin{matrix} 0 \\ 0 \\ \begin{matrix} 0 \\ 0 \\ \begin{matrix} 24.0W_{dn} \\ 0 \\ \begin{matrix} 24.0W_{nn} \\ 0 \\ \begin{matrix} 0 \\ 0 \\ \begin{matrix} 0 \\ 0 \\ \begin{matrix} 0 \\ 0 \\ \begin{matrix} 0 \\ 0 \\ \begin{matrix} 13.0W_{d} \\ 0 \\ \begin{matrix} 0.5 \\ 0 \end{matrix} \end{matrix} \end{matrix} \end{matrix} \end{matrix} \end{matrix} \end{matrix} \end{matrix} \end{matrix} \end{matrix} \end{matrix} \end{matrix} \end{matrix} \end{matrix} \end{matrix} \end{matrix} \end{matrix} \end{matrix} \end{matrix} \end{matrix} \end{matrix} \end{matrix} \end{matrix} \end{matrix}\begin{matrix} 2 \\ 5.0 \\ \begin{matrix} 0 \\ 0 \\ \begin{matrix} 0 \\ 0 \\ \begin{matrix} 0 \\ 0 \\ \begin{matrix} 0 \\ 0 \\ \begin{matrix} 0 \\ 0 \\ \begin{matrix} 0 \\ 0 \\ \begin{matrix} 0 \\ 0 \\ \begin{matrix} 0 \\ 0 \\ \begin{matrix} 0 \\ 0 \\ \begin{matrix} 0 \\ 0 \\ \begin{matrix} 0 \\ 0 \\ \begin{matrix} 0 \\ 0 \\ \begin{matrix} 0 \\ 0 \\ \begin{matrix} 0 \\ 0 \\ \begin{matrix} 0 \\ 0 \\ \begin{matrix} 0 \\ 0 \\ \begin{matrix} 0 \\ 0 \\ \begin{matrix} 0 \\ 0 \\ \begin{matrix} 0 \\ 0 \\ \begin{matrix} 0 \\ 0 \\ \begin{matrix} 0 \\ 0 \\ \begin{matrix} 0 \\ \begin{matrix} 0 \\ 0 \end{matrix} \end{matrix} \end{matrix} \end{matrix} \end{matrix} \end{matrix} \end{matrix} \end{matrix} \end{matrix} \end{matrix} \end{matrix} \end{matrix} \end{matrix} \end{matrix} \end{matrix} \end{matrix} \end{matrix} \end{matrix} \end{matrix} \end{matrix} \end{matrix} \end{matrix} \end{matrix} \end{matrix}\begin{matrix} 3 \\ 4.6 \\ \begin{matrix} 0 \\ 0 \\ \begin{matrix} 0 \\ 0 \\ \begin{matrix} 0 \\ 0 \\ \begin{matrix} 0 \\ 0 \\ \begin{matrix} 0 \\ 0 \\ \begin{matrix} 0 \\ 0 \\ \begin{matrix} 0 \\ 0 \\ \begin{matrix} 0 \\ 0 \\ \begin{matrix} 0 \\ 0 \\ \begin{matrix} 0 \\ 0 \\ \begin{matrix} 0 \\ 0 \\ \begin{matrix} 0 \\ 0 \\ \begin{matrix} 0 \\ 0 \\ \begin{matrix} 0 \\ 0 \\ \begin{matrix} 0 \\ {24.1W}_{dn} \\ \begin{matrix} 0 \\ 24.1W_{nn} \\ \begin{matrix} 0 \\ 0 \\ \begin{matrix} 0 \\ 0 \\ \begin{matrix} 0 \\ 0 \\ \begin{matrix} 0 \\ 0 \\ \begin{matrix} 0 \\ 6.9W_{d} \\ \begin{matrix} 0 \\ \begin{matrix} 0 \\ 0 \end{matrix} \end{matrix} \end{matrix} \end{matrix} \end{matrix} \end{matrix} \end{matrix} \end{matrix} \end{matrix} \end{matrix} \end{matrix} \end{matrix} \end{matrix} \end{matrix} \end{matrix} \end{matrix} \end{matrix} \end{matrix} \end{matrix} \end{matrix} \end{matrix} \end{matrix} \end{matrix} \end{matrix}\begin{matrix} 4 \\ 1.0 \\ \begin{matrix} 0 \\ 0 \\ \begin{matrix} 0 \\ 0 \\ \begin{matrix} 0 \\ 0 \\ \begin{matrix} 0 \\ 0 \\ \begin{matrix} 0 \\ 0 \\ \begin{matrix} 0 \\ 0 \\ \begin{matrix} 0 \\ 0 \\ \begin{matrix} 0 \\ 0 \\ \begin{matrix} 0 \\ 0 \\ \begin{matrix} 0 \\ 0 \\ \begin{matrix} 0 \\ 0 \\ \begin{matrix} 0 \\ 0 \\ \begin{matrix} 0 \\ 0 \\ \begin{matrix} 0 \\ 0 \\ \begin{matrix} 0 \\ 5.3W_{dn} \\ \begin{matrix} 0 \\ 5.3W_{nn} \\ \begin{matrix} 0 \\ 0 \\ \begin{matrix} 0 \\ 0 \\ \begin{matrix} 0 \\ 0 \\ \begin{matrix} 0 \\ 0 \\ \begin{matrix} 0 \\ 1.5W_{d} \\ \begin{matrix} 0 \\ \begin{matrix} 0 \\ 0 \end{matrix} \end{matrix} \end{matrix} \end{matrix} \end{matrix} \end{matrix} \end{matrix} \end{matrix} \end{matrix} \end{matrix} \end{matrix} \end{matrix} \end{matrix} \end{matrix} \end{matrix} \end{matrix} \end{matrix} \end{matrix} \end{matrix} \end{matrix} \end{matrix} \end{matrix} \end{matrix} \end{matrix}\begin{matrix} 5 \\ 0.5 \\ \begin{matrix} 0 \\ 0 \\ \begin{matrix} 0 \\ 0 \\ \begin{matrix} 0 \\ 0 \\ \begin{matrix} 0 \\ 0 \\ \begin{matrix} 0 \\ 0 \\ \begin{matrix} 0 \\ 0 \\ \begin{matrix} 0 \\ 0 \\ \begin{matrix} 0 \\ 0 \\ \begin{matrix} 0 \\ 0 \\ \begin{matrix} 0 \\ 0 \\ \begin{matrix} 0 \\ 0 \\ \begin{matrix} 0 \\ 0 \\ \begin{matrix} 0 \\ 0 \\ \begin{matrix} 0 \\ 0 \\ \begin{matrix} 0 \\ 2.4W_{dn} \\ \begin{matrix} 0 \\ 2.4W_{nn} \\ \begin{matrix} 0 \\ 0 \\ \begin{matrix} 0 \\ 0 \\ \begin{matrix} 0 \\ 0 \\ \begin{matrix} 0 \\ 0 \\ \begin{matrix} 0 \\ 0.7W_{d} \\ \begin{matrix} 0 \\ \begin{matrix} 0 \\ 0 \end{matrix} \end{matrix} \end{matrix} \end{matrix} \end{matrix} \end{matrix} \end{matrix} \end{matrix} \end{matrix} \end{matrix} \end{matrix} \end{matrix} \end{matrix} \end{matrix} \end{matrix} \end{matrix} \end{matrix} \end{matrix} \end{matrix} \end{matrix} \end{matrix} \end{matrix} \end{matrix} \end{matrix}\begin{matrix} 6 \\ 0 \\ \begin{matrix} 0 \\ 0 \\ \begin{matrix} 0 \\ 0 \\ \begin{matrix} 0 \\ 5.0 \\ \begin{matrix} 4.6 \\ 1.0 \\ \begin{matrix} 0.5 \\ 0 \\ \begin{matrix} 0 \\ 0 \\ \begin{matrix} 0 \\ 0 \\ \begin{matrix} 0 \\ 0 \\ \begin{matrix} 0 \\ 0 \\ \begin{matrix} 0 \\ 0 \\ \begin{matrix} 0 \\ 0 \\ \begin{matrix} 0 \\ 0 \\ \begin{matrix} 0 \\ 0 \\ \begin{matrix} 0 \\ 0 \\ \begin{matrix} 0 \\ 24.0W_{dn} \\ \begin{matrix} 0 \\ 24.0W_{nn} \\ \begin{matrix} 0 \\ 0 \\ \begin{matrix} 0 \\ 0 \\ \begin{matrix} 0 \\ 0 \\ \begin{matrix} 0 \\ 0 \\ \begin{matrix} 0 \\ 13.0W_{d} \\ \begin{matrix} 0 \\ \begin{matrix} 0.5 \\ 0 \end{matrix} \end{matrix} \end{matrix} \end{matrix} \end{matrix} \end{matrix} \end{matrix} \end{matrix} \end{matrix} \end{matrix} \end{matrix} \end{matrix} \end{matrix} \end{matrix} \end{matrix} \end{matrix} \end{matrix} \end{matrix} \end{matrix} \end{matrix} \end{matrix} \end{matrix} \end{matrix} \end{matrix}\begin{matrix} 7 \\ 0 \\ \begin{matrix} 0 \\ 0 \\ \begin{matrix} 0 \\ 0 \\ \begin{matrix} 5.0 \\ 0 \\ \begin{matrix} 0 \\ 0 \\ \begin{matrix} 0 \\ 0 \\ \begin{matrix} 0 \\ 0 \\ \begin{matrix} 0 \\ 0 \\ \begin{matrix} 0 \\ 0 \\ \begin{matrix} 0 \\ 0 \\ \begin{matrix} 0 \\ 0 \\ \begin{matrix} 0 \\ 0 \\ \begin{matrix} 0 \\ 0 \\ \begin{matrix} 0 \\ 0 \\ \begin{matrix} 0 \\ 0 \\ \begin{matrix} 0 \\ 0 \\ \begin{matrix} 0 \\ 0 \\ \begin{matrix} 0 \\ 0 \\ \begin{matrix} 0 \\ 0 \\ \begin{matrix} 0 \\ 0 \\ \begin{matrix} 0 \\ 0 \\ \begin{matrix} 0 \\ 0 \\ \begin{matrix} 0 \\ \begin{matrix} 0 \\ 0 \end{matrix} \end{matrix} \end{matrix} \end{matrix} \end{matrix} \end{matrix} \end{matrix} \end{matrix} \end{matrix} \end{matrix} \end{matrix} \end{matrix} \end{matrix} \end{matrix} \end{matrix} \end{matrix} \end{matrix} \end{matrix} \end{matrix} \end{matrix} \end{matrix} \end{matrix} \end{matrix} \end{matrix}\begin{matrix} 8 \\ 0 \\ \begin{matrix} 0 \\ 0 \\ \begin{matrix} 0 \\ 0 \\ \begin{matrix} 4.6 \\ 0 \\ \begin{matrix} 0 \\ 0 \\ \begin{matrix} 0 \\ 0 \\ \begin{matrix} 0 \\ 0 \\ \begin{matrix} 0 \\ 0 \\ \begin{matrix} 0 \\ 0 \\ \begin{matrix} 0 \\ 0 \\ \begin{matrix} 0 \\ 0 \\ \begin{matrix} 0 \\ 0 \\ \begin{matrix} 0 \\ 0 \\ \begin{matrix} 0 \\ 0 \\ \begin{matrix} 0 \\ 0 \\ \begin{matrix} 0 \\ 24.1W_{dn} \\ \begin{matrix} 0 \\ 24.01 \\ \begin{matrix} 0 \\ 0 \\ \begin{matrix} 0 \\ 0 \\ \begin{matrix} 0 \\ 0 \\ \begin{matrix} 0 \\ 0 \\ \begin{matrix} 0 \\ 6.9W_{d} \\ \begin{matrix} 0 \\ \begin{matrix} 0 \\ 0 \end{matrix} \end{matrix} \end{matrix} \end{matrix} \end{matrix} \end{matrix} \end{matrix} \end{matrix} \end{matrix} \end{matrix} \end{matrix} \end{matrix} \end{matrix} \end{matrix} \end{matrix} \end{matrix} \end{matrix} \end{matrix} \end{matrix} \end{matrix} \end{matrix} \end{matrix} \end{matrix} \end{matrix}\begin{matrix} 9 \\ 0 \\ \begin{matrix} 0 \\ 0 \\ \begin{matrix} 0 \\ 0 \\ \begin{matrix} 1.0 \\ 0 \\ \begin{matrix} 0 \\ 0 \\ \begin{matrix} 0 \\ 0 \\ \begin{matrix} 0 \\ 0 \\ \begin{matrix} 0 \\ 0 \\ \begin{matrix} 0 \\ 0 \\ \begin{matrix} 0 \\ 0 \\ \begin{matrix} 0 \\ 0 \\ \begin{matrix} 0 \\ 0 \\ \begin{matrix} 0 \\ 0 \\ \begin{matrix} 0 \\ 0 \\ \begin{matrix} 0 \\ 0 \\ \begin{matrix} 0 \\ 5.3W_{dn} \\ \begin{matrix} 0 \\ 5.3W_{nn} \\ \begin{matrix} 0 \\ 0 \\ \begin{matrix} 0 \\ 0 \\ \begin{matrix} 0 \\ 0 \\ \begin{matrix} 0 \\ 0 \\ \begin{matrix} 0 \\ 1.5W_{d} \\ \begin{matrix} 0 \\ \begin{matrix} 0 \\ 0 \end{matrix} \end{matrix} \end{matrix} \end{matrix} \end{matrix} \end{matrix} \end{matrix} \end{matrix} \end{matrix} \end{matrix} \end{matrix} \end{matrix} \end{matrix} \end{matrix} \end{matrix} \end{matrix} \end{matrix} \end{matrix} \end{matrix} \end{matrix} \end{matrix} \end{matrix} \end{matrix} \end{matrix}\begin{matrix} 10 \\ 0 \\ \begin{matrix} 0 \\ 0 \\ \begin{matrix} 0 \\ 0 \\ \begin{matrix} 0.5 \\ 0 \\ \begin{matrix} 0 \\ 0 \\ \begin{matrix} 0 \\ 0 \\ \begin{matrix} 0 \\ 0 \\ \begin{matrix} 0 \\ 0 \\ \begin{matrix} 0 \\ 0 \\ \begin{matrix} 0 \\ 0 \\ \begin{matrix} 0 \\ 0 \\ \begin{matrix} 0 \\ 0 \\ \begin{matrix} 0 \\ 0 \\ \begin{matrix} 0 \\ 0 \\ \begin{matrix} 0 \\ 0 \\ \begin{matrix} 0 \\ 2.5W_{dn} \\ \begin{matrix} 0 \\ 2.5W_{nn} \\ \begin{matrix} 0 \\ 0 \\ \begin{matrix} 0 \\ 0 \\ \begin{matrix} 0 \\ 0 \\ \begin{matrix} 0 \\ 0 \\ \begin{matrix} 0 \\ 0.7W_{d} \\ \begin{matrix} 0 \\ \begin{matrix} 0 \\ 0 \end{matrix} \end{matrix} \end{matrix} \end{matrix} \end{matrix} \end{matrix} \end{matrix} \end{matrix} \end{matrix} \end{matrix} \end{matrix} \end{matrix} \end{matrix} \end{matrix} \end{matrix} \end{matrix} \end{matrix} \end{matrix} \end{matrix} \end{matrix} \end{matrix} \end{matrix} \end{matrix} \end{matrix}\begin{matrix} 11 \\ \begin{matrix} 0 \\ 0 \\ \begin{matrix} 0 \\ 0 \\ \begin{matrix} 0 \\ 0 \\ \begin{matrix} 0 \\ 0 \\ \begin{matrix} 0 \\ 0 \\ \begin{matrix} 0 \\ 5.0 \\ \begin{matrix} 4.6 \\ 1.0 \\ \begin{matrix} 0.5 \\ 0 \\ \begin{matrix} 0 \\ 0 \\ \begin{matrix} 0 \\ 0 \\ \begin{matrix} 0 \\ 0 \\ \begin{matrix} 0 \\ 0 \\ \begin{matrix} 0 \\ 0 \\ \begin{matrix} 0 \\ 0 \\ \begin{matrix} 0 \\ 0 \\ \begin{matrix} 0 \\ 0 \\ \begin{matrix} 0 \\ 0 \\ \begin{matrix} 24.0W_{dn} \\ 0 \\ \begin{matrix} 24.0W_{nn} \\ 0 \\ \begin{matrix} 0 \\ 0 \\ \begin{matrix} 0 \\ 0 \\ \begin{matrix} 13.0W_{d} \\ 0 \\ \begin{matrix} 0.5 \\ 0 \end{matrix} \end{matrix} \end{matrix} \end{matrix} \end{matrix} \end{matrix} \end{matrix} \end{matrix} \end{matrix} \end{matrix} \end{matrix} \end{matrix} \end{matrix} \end{matrix} \end{matrix} \end{matrix} \end{matrix} \end{matrix} \end{matrix} \end{matrix} \end{matrix} \end{matrix} \end{matrix} \end{matrix}\begin{matrix} 12 \\ 0 \\ \begin{matrix} 0 \\ 0 \\ \begin{matrix} 0 \\ 0 \\ \begin{matrix} 0 \\ 0 \\ \begin{matrix} 0 \\ 0 \\ \begin{matrix} 0 \\ 5.0 \\ \begin{matrix} 0 \\ 0 \\ \begin{matrix} 0 \\ 0 \\ \begin{matrix} 0 \\ 0 \\ \begin{matrix} 0 \\ 0 \\ \begin{matrix} 0 \\ 0 \\ \begin{matrix} 0 \\ 0 \\ \begin{matrix} 0 \\ 0 \\ \begin{matrix} 0 \\ 0 \\ \begin{matrix} 0 \\ 0 \\ \begin{matrix} 0 \\ 0 \\ \begin{matrix} 0 \\ 0 \\ \begin{matrix} 0 \\ 0 \\ \begin{matrix} 0 \\ 0 \\ \begin{matrix} 0 \\ 0 \\ \begin{matrix} 0 \\ 0 \\ \begin{matrix} 0 \\ 0 \\ \begin{matrix} 0 \\ \begin{matrix} 0 \\ 0 \end{matrix} \end{matrix} \end{matrix} \end{matrix} \end{matrix} \end{matrix} \end{matrix} \end{matrix} \end{matrix} \end{matrix} \end{matrix} \end{matrix} \end{matrix} \end{matrix} \end{matrix} \end{matrix} \end{matrix} \end{matrix} \end{matrix} \end{matrix} \end{matrix} \end{matrix} \end{matrix} \end{matrix}\begin{matrix} 13 \\ 0 \\ \begin{matrix} 0 \\ 0 \\ \begin{matrix} 0 \\ 0 \\ \begin{matrix} 0 \\ 0 \\ \begin{matrix} 0 \\ 0 \\ \begin{matrix} 0 \\ 4.6 \\ \begin{matrix} 0 \\ 0 \\ \begin{matrix} 0 \\ 0 \\ \begin{matrix} 0 \\ 0 \\ \begin{matrix} 0 \\ 0 \\ \begin{matrix} 0 \\ 0 \\ \begin{matrix} 0 \\ 0 \\ \begin{matrix} 0 \\ 0 \\ \begin{matrix} 0 \\ 0 \\ \begin{matrix} 0 \\ 0 \\ \begin{matrix} 0 \\ 0 \\ \begin{matrix} 0 \\ 0 \\ \begin{matrix} 0 \\ 24.1W_{dn} \\ \begin{matrix} 0 \\ 24.1W_{nn} \\ \begin{matrix} 0 \\ 0 \\ \begin{matrix} 0 \\ 0 \\ \begin{matrix} 0 \\ 6.9W_{d} \\ \begin{matrix} 0 \\ \begin{matrix} 0 \\ 0 \end{matrix} \end{matrix} \end{matrix} \end{matrix} \end{matrix} \end{matrix} \end{matrix} \end{matrix} \end{matrix} \end{matrix} \end{matrix} \end{matrix} \end{matrix} \end{matrix} \end{matrix} \end{matrix} \end{matrix} \end{matrix} \end{matrix} \end{matrix} \end{matrix} \end{matrix} \end{matrix} \end{matrix}\begin{matrix} 14 \\ 0 \\ \begin{matrix} 0 \\ 0 \\ \begin{matrix} 0 \\ 0 \\ \begin{matrix} 0 \\ 0 \\ \begin{matrix} 0 \\ 0 \\ \begin{matrix} 0 \\ 1.0 \\ \begin{matrix} 0 \\ 0 \\ \begin{matrix} 0 \\ 0 \\ \begin{matrix} 0 \\ 0 \\ \begin{matrix} 0 \\ 0 \\ \begin{matrix} 0 \\ 0 \\ \begin{matrix} 0 \\ 0 \\ \begin{matrix} 0 \\ 0 \\ \begin{matrix} 0 \\ 0 \\ \begin{matrix} 0 \\ 0 \\ \begin{matrix} 0 \\ 0 \\ \begin{matrix} 0 \\ 0 \\ \begin{matrix} 0 \\ 5.3W_{dn} \\ \begin{matrix} 0 \\ 5.3W_{nn} \\ \begin{matrix} 0 \\ 0 \\ \begin{matrix} 0 \\ 0 \\ \begin{matrix} 0 \\ 1.5W_{d} \\ \begin{matrix} 0 \\ \begin{matrix} 0 \\ 0 \end{matrix} \end{matrix} \end{matrix} \end{matrix} \end{matrix} \end{matrix} \end{matrix} \end{matrix} \end{matrix} \end{matrix} \end{matrix} \end{matrix} \end{matrix} \end{matrix} \end{matrix} \end{matrix} \end{matrix} \end{matrix} \end{matrix} \end{matrix} \end{matrix} \end{matrix} \end{matrix} \end{matrix}\begin{matrix} 15 \\ 0 \\ \begin{matrix} 0 \\ 0 \\ \begin{matrix} 0 \\ 0 \\ \begin{matrix} 0 \\ 0 \\ \begin{matrix} 0 \\ 0 \\ \begin{matrix} 0 \\ 0.5 \\ \begin{matrix} 0 \\ 0 \\ \begin{matrix} 0 \\ 0 \\ \begin{matrix} 0 \\ 0 \\ \begin{matrix} 0 \\ 0 \\ \begin{matrix} 0 \\ 0 \\ \begin{matrix} 0 \\ 0 \\ \begin{matrix} 0 \\ 0 \\ \begin{matrix} 0 \\ 0 \\ \begin{matrix} 0 \\ 0 \\ \begin{matrix} 0 \\ 0 \\ \begin{matrix} 0 \\ 0 \\ \begin{matrix} 0 \\ 2.4W_{dn} \\ \begin{matrix} 0 \\ 2.4W_{nn} \\ \begin{matrix} 0 \\ 0 \\ \begin{matrix} 0 \\ 0 \\ \begin{matrix} 0 \\ 0.7W_{d} \\ \begin{matrix} 0 \\ \begin{matrix} 0 \\ 0 \end{matrix} \end{matrix} \end{matrix} \end{matrix} \end{matrix} \end{matrix} \end{matrix} \end{matrix} \end{matrix} \end{matrix} \end{matrix} \end{matrix} \end{matrix} \end{matrix} \end{matrix} \end{matrix} \end{matrix} \end{matrix} \end{matrix} \end{matrix} \end{matrix} \end{matrix} \end{matrix} \end{matrix}\begin{matrix} 16 \\ \begin{matrix} 0 \\ 0 \\ \begin{matrix} 0 \\ 0 \\ \begin{matrix} 0 \\ 0 \\ \begin{matrix} 0 \\ 0 \\ \begin{matrix} 0 \\ 0 \\ \begin{matrix} 0 \\ 0 \\ \begin{matrix} 0 \\ 0 \\ \begin{matrix} 0 \\ 0 \\ \begin{matrix} 5.0 \\ 4.6 \\ \begin{matrix} 1.0 \\ 0.5 \\ \begin{matrix} 0 \\ 0 \\ \begin{matrix} 0 \\ 0 \\ \begin{matrix} 0 \\ 0 \\ \begin{matrix} 0 \\ 0 \\ \begin{matrix} 0 \\ 0 \\ \begin{matrix} 0 \\ 0 \\ \begin{matrix} 0 \\ 0 \\ \begin{matrix} 24.0W_{dn} \\ 0 \\ \begin{matrix} 24.0W_{nn} \\ 0 \\ \begin{matrix} 0 \\ 0 \\ \begin{matrix} 0 \\ 0 \\ \begin{matrix} 6.9W_{d} \\ 0 \\ \begin{matrix} 0.7 \\ 0 \end{matrix} \end{matrix} \end{matrix} \end{matrix} \end{matrix} \end{matrix} \end{matrix} \end{matrix} \end{matrix} \end{matrix} \end{matrix} \end{matrix} \end{matrix} \end{matrix} \end{matrix} \end{matrix} \end{matrix} \end{matrix} \end{matrix} \end{matrix} \end{matrix} \end{matrix} \end{matrix} \end{matrix}\begin{matrix} 17 \\ 0 \\ \begin{matrix} 0 \\ 0 \\ \begin{matrix} 0 \\ 0 \\ \begin{matrix} 0 \\ 0 \\ \begin{matrix} 0 \\ 0 \\ \begin{matrix} 0 \\ 0 \\ \begin{matrix} 0 \\ 0 \\ \begin{matrix} 0 \\ 0 \\ \begin{matrix} 5.00 \\ 0 \\ \begin{matrix} 0 \\ 0 \\ \begin{matrix} 0 \\ 0 \\ \begin{matrix} 0 \\ 0 \\ \begin{matrix} 0 \\ 0 \\ \begin{matrix} 0 \\ 0 \\ \begin{matrix} 0 \\ 0 \\ \begin{matrix} 0 \\ 0 \\ \begin{matrix} 0 \\ 0 \\ \begin{matrix} 0 \\ 0 \\ \begin{matrix} 0 \\ 0 \\ \begin{matrix} 0 \\ 0 \\ \begin{matrix} 0 \\ 0 \\ \begin{matrix} 0 \\ 0 \\ \begin{matrix} 0 \\ \begin{matrix} 0 \\ 0 \end{matrix} \end{matrix} \end{matrix} \end{matrix} \end{matrix} \end{matrix} \end{matrix} \end{matrix} \end{matrix} \end{matrix} \end{matrix} \end{matrix} \end{matrix} \end{matrix} \end{matrix} \end{matrix} \end{matrix} \end{matrix} \end{matrix} \end{matrix} \end{matrix} \end{matrix} \end{matrix} \end{matrix}\begin{matrix} 18 \\ 0 \\ \begin{matrix} 0 \\ 0 \\ \begin{matrix} 0 \\ 0 \\ \begin{matrix} 0 \\ 0 \\ \begin{matrix} 0 \\ 0 \\ \begin{matrix} 0 \\ 0 \\ \begin{matrix} 0 \\ 0 \\ \begin{matrix} 0 \\ 0 \\ \begin{matrix} 4.6 \\ 0 \\ \begin{matrix} 0 \\ 0 \\ \begin{matrix} 0 \\ 0 \\ \begin{matrix} 0 \\ 0 \\ \begin{matrix} 0 \\ 0 \\ \begin{matrix} 0 \\ 0 \\ \begin{matrix} 0 \\ 0 \\ \begin{matrix} 0 \\ 0 \\ \begin{matrix} 0 \\ 0 \\ \begin{matrix} 0 \\ 24.1W_{dn} \\ \begin{matrix} 0 \\ 24.1W_{nn} \\ \begin{matrix} 0 \\ 0 \\ \begin{matrix} 0 \\ 0 \\ \begin{matrix} 0 \\ 6.9W_{d} \\ \begin{matrix} 0 \\ \begin{matrix} 0 \\ 0 \end{matrix} \end{matrix} \end{matrix} \end{matrix} \end{matrix} \end{matrix} \end{matrix} \end{matrix} \end{matrix} \end{matrix} \end{matrix} \end{matrix} \end{matrix} \end{matrix} \end{matrix} \end{matrix} \end{matrix} \end{matrix} \end{matrix} \end{matrix} \end{matrix} \end{matrix} \end{matrix} \end{matrix}\begin{matrix} 19 \\ 0 \\ \begin{matrix} 0 \\ 0 \\ \begin{matrix} 0 \\ 0 \\ \begin{matrix} 0 \\ 0 \\ \begin{matrix} 0 \\ 0 \\ \begin{matrix} 0 \\ 0 \\ \begin{matrix} 0 \\ 0 \\ \begin{matrix} 0 \\ 0 \\ \begin{matrix} 1.0 \\ 0 \\ \begin{matrix} 0 \\ 0 \\ \begin{matrix} 0 \\ 0 \\ \begin{matrix} 0 \\ 0 \\ \begin{matrix} 0 \\ 0 \\ \begin{matrix} 0 \\ 0 \\ \begin{matrix} 0 \\ 0 \\ \begin{matrix} 0 \\ 0 \\ \begin{matrix} 0 \\ 0 \\ \begin{matrix} 0 \\ 5.3W_{dn} \\ \begin{matrix} 0 \\ 5.3W_{nn} \\ \begin{matrix} 0 \\ 0 \\ \begin{matrix} 0 \\ 0 \\ \begin{matrix} 0 \\ 1.5W_{d} \\ \begin{matrix} 0 \\ \begin{matrix} 0 \\ 0 \end{matrix} \end{matrix} \end{matrix} \end{matrix} \end{matrix} \end{matrix} \end{matrix} \end{matrix} \end{matrix} \end{matrix} \end{matrix} \end{matrix} \end{matrix} \end{matrix} \end{matrix} \end{matrix} \end{matrix} \end{matrix} \end{matrix} \end{matrix} \end{matrix} \end{matrix} \end{matrix} \end{matrix}\begin{matrix} 20 \\ 0 \\ \begin{matrix} 0 \\ 0 \\ \begin{matrix} 0 \\ 0 \\ \begin{matrix} 0 \\ 0 \\ \begin{matrix} 0 \\ 0 \\ \begin{matrix} 0 \\ 0 \\ \begin{matrix} 0 \\ 0 \\ \begin{matrix} 0 \\ 0 \\ \begin{matrix} 0.5 \\ 0 \\ \begin{matrix} 0 \\ 0 \\ \begin{matrix} 0 \\ 0 \\ \begin{matrix} 0 \\ 0 \\ \begin{matrix} 0 \\ 0 \\ \begin{matrix} 0 \\ 0 \\ \begin{matrix} 0 \\ 0 \\ \begin{matrix} 0 \\ 0 \\ \begin{matrix} 0 \\ 0 \\ \begin{matrix} 0 \\ 2.5W_{dn} \\ \begin{matrix} 0 \\ 2.5W_{nn} \\ \begin{matrix} 0 \\ 0 \\ \begin{matrix} 0 \\ 0 \\ \begin{matrix} 0 \\ 0.7W_{d} \\ \begin{matrix} 0 \\ \begin{matrix} 0 \\ 0 \end{matrix} \end{matrix} \end{matrix} \end{matrix} \end{matrix} \end{matrix} \end{matrix} \end{matrix} \end{matrix} \end{matrix} \end{matrix} \end{matrix} \end{matrix} \end{matrix} \end{matrix} \end{matrix} \end{matrix} \end{matrix} \end{matrix} \end{matrix} \end{matrix} \end{matrix} \end{matrix} \end{matrix}\begin{matrix} 21 \\ 0 \\ \begin{matrix} 0 \\ 0 \\ \begin{matrix} 0 \\ 0 \\ \begin{matrix} 0 \\ 0 \\ \begin{matrix} 0 \\ 0 \\ \begin{matrix} 0 \\ 0 \\ \begin{matrix} 0 \\ 0 \\ \begin{matrix} 0 \\ 0 \\ \begin{matrix} 0 \\ 0 \\ \begin{matrix} 0 \\ 0 \\ \begin{matrix} 0 \\ 0 \\ \begin{matrix} 5.0 \\ 4.6 \\ \begin{matrix} 1.0 \\ 0.5 \\ \begin{matrix} 0 \\ 0 \\ \begin{matrix} 0 \\ 0 \\ \begin{matrix} 0 \\ 0 \\ \begin{matrix} 0 \\ 0 \\ \begin{matrix} 0 \\ 0 \\ \begin{matrix} 0 \\ 0 \\ \begin{matrix} 0 \\ 24.0W_{dn} \\ \begin{matrix} 0 \\ 24.0W_{nn} \\ \begin{matrix} 0 \\ 13.0W_{d} \\ \begin{matrix} 0 \\ \begin{matrix} 0.5 \\ 0 \end{matrix} \end{matrix} \end{matrix} \end{matrix} \end{matrix} \end{matrix} \end{matrix} \end{matrix} \end{matrix} \end{matrix} \end{matrix} \end{matrix} \end{matrix} \end{matrix} \end{matrix} \end{matrix} \end{matrix} \end{matrix} \end{matrix} \end{matrix} \end{matrix} \end{matrix} \end{matrix} \end{matrix}\begin{matrix} 22 \\ 0 \\ \begin{matrix} 0 \\ 0 \\ \begin{matrix} 0 \\ 0 \\ \begin{matrix} 0 \\ 0 \\ \begin{matrix} 0 \\ 0 \\ \begin{matrix} 0 \\ 0 \\ \begin{matrix} 0 \\ 0 \\ \begin{matrix} 0 \\ 0 \\ \begin{matrix} 0 \\ 0 \\ \begin{matrix} 0 \\ 0 \\ \begin{matrix} 0 \\ 5.0 \\ \begin{matrix} 0 \\ 0 \\ \begin{matrix} 0 \\ 0 \\ \begin{matrix} 0 \\ 0 \\ \begin{matrix} 0 \\ 0 \\ \begin{matrix} 0 \\ 0 \\ \begin{matrix} 0 \\ 0 \\ \begin{matrix} 0 \\ 0 \\ \begin{matrix} 0 \\ 0 \\ \begin{matrix} 0 \\ 0 \\ \begin{matrix} 0 \\ 0 \\ \begin{matrix} 0 \\ 0 \\ \begin{matrix} 0 \\ \begin{matrix} 0 \\ 0 \end{matrix} \end{matrix} \end{matrix} \end{matrix} \end{matrix} \end{matrix} \end{matrix} \end{matrix} \end{matrix} \end{matrix} \end{matrix} \end{matrix} \end{matrix} \end{matrix} \end{matrix} \end{matrix} \end{matrix} \end{matrix} \end{matrix} \end{matrix} \end{matrix} \end{matrix} \end{matrix} \end{matrix}\begin{matrix} 23 \\ 0 \\ \begin{matrix} 0 \\ 0 \\ \begin{matrix} 0 \\ 0 \\ \begin{matrix} 0 \\ 0 \\ \begin{matrix} 0 \\ 0 \\ \begin{matrix} 0 \\ 0 \\ \begin{matrix} 0 \\ 0 \\ \begin{matrix} 0 \\ 0 \\ \begin{matrix} 0 \\ 0 \\ \begin{matrix} 0 \\ 0 \\ \begin{matrix} 0 \\ 4.6 \\ \begin{matrix} 0 \\ 0 \\ \begin{matrix} 0 \\ 0 \\ \begin{matrix} 0 \\ 0 \\ \begin{matrix} 0 \\ 0 \\ \begin{matrix} 0 \\ 0 \\ \begin{matrix} 0 \\ 0 \\ \begin{matrix} 0 \\ 0 \\ \begin{matrix} 0 \\ 0 \\ \begin{matrix} 0 \\ 24.1W_{dn} \\ \begin{matrix} 0 \\ 24.1W_{nn} \\ \begin{matrix} 0 \\ 6.9W_{d} \\ \begin{matrix} 0 \\ \begin{matrix} 0 \\ 0 \end{matrix} \end{matrix} \end{matrix} \end{matrix} \end{matrix} \end{matrix} \end{matrix} \end{matrix} \end{matrix} \end{matrix} \end{matrix} \end{matrix} \end{matrix} \end{matrix} \end{matrix} \end{matrix} \end{matrix} \end{matrix} \end{matrix} \end{matrix} \end{matrix} \end{matrix} \end{matrix} \end{matrix}\begin{matrix} 24 \\ 0 \\ \begin{matrix} 0 \\ 0 \\ \begin{matrix} 0 \\ 0 \\ \begin{matrix} 0 \\ 0 \\ \begin{matrix} 0 \\ 0 \\ \begin{matrix} 0 \\ 0 \\ \begin{matrix} 0 \\ 0 \\ \begin{matrix} 0 \\ 0 \\ \begin{matrix} 0 \\ 0 \\ \begin{matrix} 0 \\ 0 \\ \begin{matrix} 0 \\ 1.0 \\ \begin{matrix} 0 \\ 0 \\ \begin{matrix} 0 \\ 0 \\ \begin{matrix} 0 \\ 0 \\ \begin{matrix} 0 \\ 0 \\ \begin{matrix} 0 \\ 0 \\ \begin{matrix} 0 \\ 0 \\ \begin{matrix} 0 \\ 0 \\ \begin{matrix} 0 \\ 0 \\ \begin{matrix} 0 \\ 5.3W_{dn} \\ \begin{matrix} 0 \\ 5.3W_{nn} \\ \begin{matrix} 0 \\ 1.5W_{d} \\ \begin{matrix} 0 \\ \begin{matrix} 0 \\ 0 \end{matrix} \end{matrix} \end{matrix} \end{matrix} \end{matrix} \end{matrix} \end{matrix} \end{matrix} \end{matrix} \end{matrix} \end{matrix} \end{matrix} \end{matrix} \end{matrix} \end{matrix} \end{matrix} \end{matrix} \end{matrix} \end{matrix} \end{matrix} \end{matrix} \end{matrix} \end{matrix} \end{matrix}\begin{matrix} 25 \\ 0 \\ \begin{matrix} 0 \\ 0 \\ \begin{matrix} 0 \\ 0 \\ \begin{matrix} 0 \\ 0 \\ \begin{matrix} 0 \\ 0 \\ \begin{matrix} 0 \\ 0 \\ \begin{matrix} 0 \\ 0 \\ \begin{matrix} 0 \\ 0 \\ \begin{matrix} 0 \\ 0 \\ \begin{matrix} 0 \\ 0 \\ \begin{matrix} 0 \\ 0.5 \\ \begin{matrix} 0 \\ 0 \\ \begin{matrix} 0 \\ 0 \\ \begin{matrix} 0 \\ 0 \\ \begin{matrix} 0 \\ 0 \\ \begin{matrix} 0 \\ 0 \\ \begin{matrix} 0 \\ 0 \\ \begin{matrix} 0 \\ 0 \\ \begin{matrix} 0 \\ 0 \\ \begin{matrix} 0 \\ 2.4W_{dn} \\ \begin{matrix} 0 \\ 2.4W_{nn} \\ \begin{matrix} 0 \\ 0.7W_{d} \\ \begin{matrix} 0 \\ \begin{matrix} 0 \\ 0 \end{matrix} \end{matrix} \end{matrix} \end{matrix} \end{matrix} \end{matrix} \end{matrix} \end{matrix} \end{matrix} \end{matrix} \end{matrix} \end{matrix} \end{matrix} \end{matrix} \end{matrix} \end{matrix} \end{matrix} \end{matrix} \end{matrix} \end{matrix} \end{matrix} \end{matrix} \end{matrix} \end{matrix}\begin{matrix} 26 \\ 0 \\ \begin{matrix} 0 \\ 0 \\ \begin{matrix} 0 \\ 0 \\ \begin{matrix} 0 \\ 0 \\ \begin{matrix} 0 \\ 0 \\ \begin{matrix} 0 \\ 0 \\ \begin{matrix} 0 \\ 0 \\ \begin{matrix} 0 \\ 0 \\ \begin{matrix} 0 \\ 0 \\ \begin{matrix} 0 \\ 0 \\ \begin{matrix} 0 \\ 0 \\ \begin{matrix} 0 \\ 0 \\ \begin{matrix} 0 \\ 0 \\ \begin{matrix} 0 \\ 5.0 \\ \begin{matrix} 4.6 \\ 1.0 \\ \begin{matrix} 0.5 \\ 0 \\ \begin{matrix} 0 \\ 0 \\ \begin{matrix} 0 \\ 0 \\ \begin{matrix} 0 \\ 0 \\ \begin{matrix} 0 \\ 24.0W_{dn} \\ \begin{matrix} 0 \\ 24.0W_{nn} \\ \begin{matrix} 0 \\ 13.0W_{d} \\ \begin{matrix} 0 \\ \begin{matrix} 0.5 \\ 0 \end{matrix} \end{matrix} \end{matrix} \end{matrix} \end{matrix} \end{matrix} \end{matrix} \end{matrix} \end{matrix} \end{matrix} \end{matrix} \end{matrix} \end{matrix} \end{matrix} \end{matrix} \end{matrix} \end{matrix} \end{matrix} \end{matrix} \end{matrix} \end{matrix} \end{matrix} \end{matrix} \end{matrix}\begin{matrix} 27 \\ 0 \\ \begin{matrix} 0 \\ 0 \\ \begin{matrix} 0 \\ 0 \\ \begin{matrix} 0 \\ 0 \\ \begin{matrix} 0 \\ 0 \\ \begin{matrix} 0 \\ 0 \\ \begin{matrix} 0 \\ 0 \\ \begin{matrix} 0 \\ 0 \\ \begin{matrix} 0 \\ 0 \\ \begin{matrix} 0 \\ 0 \\ \begin{matrix} 0 \\ 0 \\ \begin{matrix} 0 \\ 0 \\ \begin{matrix} 0 \\ 0 \\ \begin{matrix} 5.0 \\ 0 \\ \begin{matrix} 0 \\ 0 \\ \begin{matrix} 0 \\ 0 \\ \begin{matrix} 0 \\ 0 \\ \begin{matrix} 0 \\ 0 \\ \begin{matrix} 0 \\ 0 \\ \begin{matrix} 0 \\ 0 \\ \begin{matrix} 0 \\ 0 \\ \begin{matrix} 0 \\ 0 \\ \begin{matrix} 0 \\ \begin{matrix} 0 \\ 0 \end{matrix} \end{matrix} \end{matrix} \end{matrix} \end{matrix} \end{matrix} \end{matrix} \end{matrix} \end{matrix} \end{matrix} \end{matrix} \end{matrix} \end{matrix} \end{matrix} \end{matrix} \end{matrix} \end{matrix} \end{matrix} \end{matrix} \end{matrix} \end{matrix} \end{matrix} \end{matrix} \end{matrix}\begin{matrix} 28 \\ 0 \\ \begin{matrix} 0 \\ 0 \\ \begin{matrix} 0 \\ 0 \\ \begin{matrix} 0 \\ 0 \\ \begin{matrix} 0 \\ 0 \\ \begin{matrix} 0 \\ 0 \\ \begin{matrix} 0 \\ 0 \\ \begin{matrix} 0 \\ 0 \\ \begin{matrix} 0 \\ 0 \\ \begin{matrix} 0 \\ 0 \\ \begin{matrix} 0 \\ 0 \\ \begin{matrix} 0 \\ 0 \\ \begin{matrix} 0 \\ 0 \\ \begin{matrix} 4.6 \\ 0 \\ \begin{matrix} 0 \\ 0 \\ \begin{matrix} 0 \\ 0 \\ \begin{matrix} 0 \\ 0 \\ \begin{matrix} 0 \\ 0 \\ \begin{matrix} 0 \\ 0 \\ \begin{matrix} 0 \\ 24.1W_{dn} \\ \begin{matrix} 0 \\ 24.1W_{nn} \\ \begin{matrix} 0 \\ 6.9W_{d} \\ \begin{matrix} 0 \\ \begin{matrix} 0 \\ 0 \end{matrix} \end{matrix} \end{matrix} \end{matrix} \end{matrix} \end{matrix} \end{matrix} \end{matrix} \end{matrix} \end{matrix} \end{matrix} \end{matrix} \end{matrix} \end{matrix} \end{matrix} \end{matrix} \end{matrix} \end{matrix} \end{matrix} \end{matrix} \end{matrix} \end{matrix} \end{matrix} \end{matrix}\begin{matrix} 29 \\ 0 \\ \begin{matrix} 0 \\ 0 \\ \begin{matrix} 0 \\ 0 \\ \begin{matrix} 0 \\ 0 \\ \begin{matrix} 0 \\ 0 \\ \begin{matrix} 0 \\ 0 \\ \begin{matrix} 0 \\ 0 \\ \begin{matrix} 0 \\ 0 \\ \begin{matrix} 0 \\ 0 \\ \begin{matrix} 0 \\ 0 \\ \begin{matrix} 0 \\ 0 \\ \begin{matrix} 0 \\ 0 \\ \begin{matrix} 0 \\ 0 \\ \begin{matrix} 1.0 \\ 0 \\ \begin{matrix} 0 \\ 0 \\ \begin{matrix} 0 \\ 0 \\ \begin{matrix} 0 \\ 0 \\ \begin{matrix} 0 \\ 0 \\ \begin{matrix} 0 \\ 0 \\ \begin{matrix} 0 \\ 5.3W_{dn} \\ \begin{matrix} 0 \\ 5.3W_{nn} \\ \begin{matrix} 0 \\ 1.5W_{d} \\ \begin{matrix} 0 \\ \begin{matrix} 0 \\ 0 \end{matrix} \end{matrix} \end{matrix} \end{matrix} \end{matrix} \end{matrix} \end{matrix} \end{matrix} \end{matrix} \end{matrix} \end{matrix} \end{matrix} \end{matrix} \end{matrix} \end{matrix} \end{matrix} \end{matrix} \end{matrix} \end{matrix} \end{matrix} \end{matrix} \end{matrix} \end{matrix} \end{matrix}\begin{matrix} 30 \\ 0 \\ \begin{matrix} 0 \\ 0 \\ \begin{matrix} 0 \\ 0 \\ \begin{matrix} 0 \\ 0 \\ \begin{matrix} 0 \\ 0 \\ \begin{matrix} 0 \\ 0 \\ \begin{matrix} 0 \\ 0 \\ \begin{matrix} 0 \\ 0 \\ \begin{matrix} 0 \\ 0 \\ \begin{matrix} 0 \\ 0 \\ \begin{matrix} 0 \\ 0 \\ \begin{matrix} 0 \\ 0 \\ \begin{matrix} 0 \\ 0 \\ \begin{matrix} 0.5 \\ 0 \\ \begin{matrix} 0 \\ 0 \\ \begin{matrix} 0 \\ 0 \\ \begin{matrix} 0 \\ 0 \\ \begin{matrix} 0 \\ 0 \\ \begin{matrix} 0 \\ 0 \\ \begin{matrix} 0 \\ 2.4W_{dn} \\ \begin{matrix} 0 \\ 2.4W_{nn} \\ \begin{matrix} 0 \\ 0.7W_{d} \\ \begin{matrix} 0 \\ \begin{matrix} 0 \\ 0 \end{matrix} \end{matrix} \end{matrix} \end{matrix} \end{matrix} \end{matrix} \end{matrix} \end{matrix} \end{matrix} \end{matrix} \end{matrix} \end{matrix} \end{matrix} \end{matrix} \end{matrix} \end{matrix} \end{matrix} \end{matrix} \end{matrix} \end{matrix} \end{matrix} \end{matrix} \end{matrix} \end{matrix}\begin{matrix} 31 \\ \begin{matrix} 24.0W_{dn} \\ 0 \\ \begin{matrix} 24.1W_{dn} \\ 5.3W_{dn} \\ \begin{matrix} 2.5W_{dn} \\ 24.0W_{dn} \\ \begin{matrix} 0 \\ 24.1W_{dn} \\ \begin{matrix} 5.3W_{dn} \\ 2.5W_{dn} \\ \begin{matrix} 0 \\ 0 \\ \begin{matrix} 0 \\ 0 \\ \begin{matrix} 0 \\ 0 \\ \begin{matrix} 0 \\ 0 \\ \begin{matrix} 0 \\ 0 \\ \begin{matrix} 0 \\ 0 \\ \begin{matrix} 0 \\ 0 \\ \begin{matrix} 0 \\ 0 \\ \begin{matrix} 0 \\ 0 \\ \begin{matrix} 0 \\ 0 \\ \begin{matrix} 5.0 \\ 0 \\ \begin{matrix} 0 \\ 0 \\ \begin{matrix} 0 \\ 0 \\ \begin{matrix} 0 \\ 0 \\ \begin{matrix} 0 \\ 0 \\ \begin{matrix} 0 \\ 0 \\ \begin{matrix} 0 \\ 0 \\ \begin{matrix} {2.5W}_{dn} \\ 4.9W_{dn} \end{matrix} \end{matrix} \end{matrix} \end{matrix} \end{matrix} \end{matrix} \end{matrix} \end{matrix} \end{matrix} \end{matrix} \end{matrix} \end{matrix} \end{matrix} \end{matrix} \end{matrix} \end{matrix} \end{matrix} \end{matrix} \end{matrix} \end{matrix} \end{matrix} \end{matrix} \end{matrix} \end{matrix}\begin{matrix} 32 \\ 0 \\ \begin{matrix} 0 \\ 0 \\ \begin{matrix} 0 \\ 0 \\ \begin{matrix} 0 \\ 0 \\ \begin{matrix} 0 \\ 0 \\ \begin{matrix} 0 \\ 0 \\ \begin{matrix} 0 \\ 0 \\ \begin{matrix} 0 \\ 0 \\ \begin{matrix} 0 \\ 0 \\ \begin{matrix} 0 \\ 0 \\ \begin{matrix} 0 \\ 0 \\ \begin{matrix} 0 \\ 0 \\ \begin{matrix} 0 \\ 0 \\ \begin{matrix} 0 \\ 0 \\ \begin{matrix} 0 \\ 0 \\ \begin{matrix} 5.0 \\ 0 \\ \begin{matrix} 0 \\ 0 \\ \begin{matrix} 0 \\ 0 \\ \begin{matrix} 0 \\ 0 \\ \begin{matrix} 0 \\ 0 \\ \begin{matrix} 0 \\ 0 \\ \begin{matrix} 0 \\ 0 \\ \begin{matrix} 0 \\ \begin{matrix} 0 \\ 0 \end{matrix} \end{matrix} \end{matrix} \end{matrix} \end{matrix} \end{matrix} \end{matrix} \end{matrix} \end{matrix} \end{matrix} \end{matrix} \end{matrix} \end{matrix} \end{matrix} \end{matrix} \end{matrix} \end{matrix} \end{matrix} \end{matrix} \end{matrix} \end{matrix} \end{matrix} \end{matrix} \end{matrix}\begin{matrix} 33 \\ \begin{matrix} 24.0W_{nn} \\ 0 \\ \begin{matrix} 24.1W_{nn} \\ 5.3W_{nn} \\ \begin{matrix} 2.5W_{nn} \\ 24.0W_{nn} \\ \begin{matrix} 0 \\ 24.1W_{nn} \\ \begin{matrix} 5.3W_{nn} \\ 2.5W_{nn} \\ \begin{matrix} 0 \\ 0 \\ \begin{matrix} 0 \\ 0 \\ \begin{matrix} 0 \\ 0 \\ \begin{matrix} 0 \\ 0 \\ \begin{matrix} 0 \\ 0 \\ \begin{matrix} 0 \\ 0 \\ \begin{matrix} 0 \\ 0 \\ \begin{matrix} 0 \\ 0 \\ \begin{matrix} 0 \\ 0 \\ \begin{matrix} 0 \\ 0 \\ \begin{matrix} 0 \\ 0 \\ \begin{matrix} 5.0 \\ 0 \\ \begin{matrix} 0 \\ 0 \\ \begin{matrix} 0 \\ 0 \\ \begin{matrix} 0 \\ 0 \\ \begin{matrix} 0 \\ 0 \\ \begin{matrix} 0 \\ 0 \\ \begin{matrix} {2.5W}_{nn} \\ 4.9W_{nn} \end{matrix} \end{matrix} \end{matrix} \end{matrix} \end{matrix} \end{matrix} \end{matrix} \end{matrix} \end{matrix} \end{matrix} \end{matrix} \end{matrix} \end{matrix} \end{matrix} \end{matrix} \end{matrix} \end{matrix} \end{matrix} \end{matrix} \end{matrix} \end{matrix} \end{matrix} \end{matrix} \end{matrix}\begin{matrix} 34 \\ 0 \\ \begin{matrix} 0 \\ 0 \\ \begin{matrix} 0 \\ 0 \\ \begin{matrix} 0 \\ 0 \\ \begin{matrix} 0 \\ 0 \\ \begin{matrix} 0 \\ 0 \\ \begin{matrix} 0 \\ 0 \\ \begin{matrix} 0 \\ 0 \\ \begin{matrix} 0 \\ 0 \\ \begin{matrix} 0 \\ 0 \\ \begin{matrix} 0 \\ 0 \\ \begin{matrix} 0 \\ 0 \\ \begin{matrix} 0 \\ 0 \\ \begin{matrix} 0 \\ 0 \\ \begin{matrix} 0 \\ 0 \\ \begin{matrix} 0 \\ 0 \\ \begin{matrix} 5.0 \\ 0 \\ \begin{matrix} 0 \\ 0 \\ \begin{matrix} 0 \\ 0 \\ \begin{matrix} 0 \\ 0 \\ \begin{matrix} 0 \\ 0 \\ \begin{matrix} 0 \\ 0 \\ \begin{matrix} 0 \\ \begin{matrix} 0 \\ 0 \end{matrix} \end{matrix} \end{matrix} \end{matrix} \end{matrix} \end{matrix} \end{matrix} \end{matrix} \end{matrix} \end{matrix} \end{matrix} \end{matrix} \end{matrix} \end{matrix} \end{matrix} \end{matrix} \end{matrix} \end{matrix} \end{matrix} \end{matrix} \end{matrix} \end{matrix} \end{matrix} \end{matrix}\begin{matrix} 35 \\ 0 \\ \begin{matrix} 0 \\ 0 \\ \begin{matrix} 0 \\ 0 \\ \begin{matrix} 0 \\ 0 \\ \begin{matrix} 0 \\ 0 \\ \begin{matrix} 0 \\ 24.0W_{dn} \\ \begin{matrix} 0 \\ 24.1W_{dn} \\ \begin{matrix} 5.3W_{dn} \\ 2.5W_{dn} \\ \begin{matrix} 24.0W_{dn} \\ 0 \\ \begin{matrix} 24.1W_{dn} \\ 5.3W_{dn} \\ \begin{matrix} 2.5W_{dn} \\ 0 \\ \begin{matrix} 0 \\ 0 \\ \begin{matrix} 0 \\ 0 \\ \begin{matrix} 0 \\ 0 \\ \begin{matrix} 0 \\ 0 \\ \begin{matrix} 0 \\ 0 \\ \begin{matrix} 0 \\ 0 \\ \begin{matrix} 0 \\ 5.0 \\ \begin{matrix} 0 \\ 0 \\ \begin{matrix} 0 \\ 0 \\ \begin{matrix} 0 \\ 0 \\ \begin{matrix} 0 \\ 0 \\ \begin{matrix} 0 \\ \begin{matrix} {2.5W}_{dn} \\ 4.9W_{dn} \end{matrix} \end{matrix} \end{matrix} \end{matrix} \end{matrix} \end{matrix} \end{matrix} \end{matrix} \end{matrix} \end{matrix} \end{matrix} \end{matrix} \end{matrix} \end{matrix} \end{matrix} \end{matrix} \end{matrix} \end{matrix} \end{matrix} \end{matrix} \end{matrix} \end{matrix} \end{matrix} \end{matrix}\begin{matrix} 36 \\ 0 \\ \begin{matrix} 0 \\ 0 \\ \begin{matrix} 0 \\ 0 \\ \begin{matrix} 0 \\ 0 \\ \begin{matrix} 0 \\ 0 \\ \begin{matrix} 0 \\ 0 \\ \begin{matrix} 0 \\ 0 \\ \begin{matrix} 0 \\ 0 \\ \begin{matrix} 0 \\ 0 \\ \begin{matrix} 0 \\ 0 \\ \begin{matrix} 0 \\ 0 \\ \begin{matrix} 0 \\ 0 \\ \begin{matrix} 0 \\ 0 \\ \begin{matrix} 0 \\ 0 \\ \begin{matrix} 0 \\ 0 \\ \begin{matrix} 0 \\ 0 \\ \begin{matrix} 0 \\ 0 \\ \begin{matrix} 5.0 \\ 0 \\ \begin{matrix} 0 \\ 0 \\ \begin{matrix} 0 \\ 0 \\ \begin{matrix} 0 \\ 0 \\ \begin{matrix} 0 \\ 0 \\ \begin{matrix} 0 \\ \begin{matrix} 0 \\ 0 \end{matrix} \end{matrix} \end{matrix} \end{matrix} \end{matrix} \end{matrix} \end{matrix} \end{matrix} \end{matrix} \end{matrix} \end{matrix} \end{matrix} \end{matrix} \end{matrix} \end{matrix} \end{matrix} \end{matrix} \end{matrix} \end{matrix} \end{matrix} \end{matrix} \end{matrix} \end{matrix} \end{matrix}\begin{matrix} 37 \\ 0 \\ \begin{matrix} 0 \\ 0 \\ \begin{matrix} 0 \\ 0 \\ \begin{matrix} 0 \\ 0 \\ \begin{matrix} 0 \\ 0 \\ \begin{matrix} 0 \\ 6.60W_{nn} \\ \begin{matrix} 0 \\ 15.44W_{nn} \\ \begin{matrix} 3.44W_{nn} \\ 1.56W_{nn} \\ \begin{matrix} 6.60W_{nn} \\ 0 \\ \begin{matrix} 15.44W_{nn} \\ 3.44W_{nn} \\ \begin{matrix} 1.56W_{nn} \\ 0 \\ \begin{matrix} 0 \\ 0 \\ \begin{matrix} 0 \\ 0 \\ \begin{matrix} 0 \\ 0 \\ \begin{matrix} 0 \\ 0 \\ \begin{matrix} 0 \\ 0 \\ \begin{matrix} 0 \\ 0 \\ \begin{matrix} 0 \\ 0 \\ \begin{matrix} 0 \\ 5.0 \\ \begin{matrix} 0 \\ 0 \\ \begin{matrix} 0 \\ 0 \\ \begin{matrix} 0 \\ 0 \\ \begin{matrix} 0 \\ \begin{matrix} {2.5W}_{nn} \\ 4.9W_{nn} \end{matrix} \end{matrix} \end{matrix} \end{matrix} \end{matrix} \end{matrix} \end{matrix} \end{matrix} \end{matrix} \end{matrix} \end{matrix} \end{matrix} \end{matrix} \end{matrix} \end{matrix} \end{matrix} \end{matrix} \end{matrix} \end{matrix} \end{matrix} \end{matrix} \end{matrix} \end{matrix} \end{matrix}\begin{matrix} 38 \\ 0 \\ \begin{matrix} 0 \\ 0 \\ \begin{matrix} 0 \\ 0 \\ \begin{matrix} 0 \\ 0 \\ \begin{matrix} 0 \\ 0 \\ \begin{matrix} 0 \\ 0 \\ \begin{matrix} 0 \\ 0 \\ \begin{matrix} 0 \\ 0 \\ \begin{matrix} 0 \\ 0 \\ \begin{matrix} 0 \\ 0 \\ \begin{matrix} 0 \\ 0 \\ \begin{matrix} 0 \\ 0 \\ \begin{matrix} 0 \\ 0 \\ \begin{matrix} 0 \\ 0 \\ \begin{matrix} 0 \\ 0 \\ \begin{matrix} 0 \\ 0 \\ \begin{matrix} 0 \\ 0 \\ \begin{matrix} 0 \\ 0 \\ \begin{matrix} 5.0 \\ 0 \\ \begin{matrix} 0 \\ 0 \\ \begin{matrix} 0 \\ 0 \\ \begin{matrix} 0 \\ 0 \\ \begin{matrix} 0 \\ \begin{matrix} 0 \\ 0 \end{matrix} \end{matrix} \end{matrix} \end{matrix} \end{matrix} \end{matrix} \end{matrix} \end{matrix} \end{matrix} \end{matrix} \end{matrix} \end{matrix} \end{matrix} \end{matrix} \end{matrix} \end{matrix} \end{matrix} \end{matrix} \end{matrix} \end{matrix} \end{matrix} \end{matrix} \end{matrix} \end{matrix}\begin{matrix} 39 \\ 0 \\ \begin{matrix} 0 \\ 0 \\ \begin{matrix} 0 \\ 0 \\ \begin{matrix} 0 \\ 0 \\ \begin{matrix} 0 \\ 0 \\ \begin{matrix} 0 \\ 0 \\ \begin{matrix} 0 \\ 0 \\ \begin{matrix} 0 \\ 0 \\ \begin{matrix} 0 \\ 0 \\ \begin{matrix} 0 \\ 0 \\ \begin{matrix} 0 \\ 24.0W_{dn} \\ \begin{matrix} 0 \\ 24.1W_{dn} \\ \begin{matrix} 5.3W_{dn} \\ {2.5W}_{dn} \\ \begin{matrix} 24.0W_{dn} \\ 0 \\ \begin{matrix} 24.1W_{dn} \\ 5.3W_{dn} \\ \begin{matrix} 2.5W_{dn} \\ 0 \\ \begin{matrix} 0 \\ 0 \\ \begin{matrix} 0 \\ 0 \\ \begin{matrix} 0 \\ 0 \\ \begin{matrix} 0 \\ 0 \\ \begin{matrix} 5.0 \\ 0 \\ \begin{matrix} 0 \\ 0 \\ \begin{matrix} 0 \\ \begin{matrix} {2.5W}_{dn} \\ 4.9W_{dn} \end{matrix} \end{matrix} \end{matrix} \end{matrix} \end{matrix} \end{matrix} \end{matrix} \end{matrix} \end{matrix} \end{matrix} \end{matrix} \end{matrix} \end{matrix} \end{matrix} \end{matrix} \end{matrix} \end{matrix} \end{matrix} \end{matrix} \end{matrix} \end{matrix} \end{matrix} \end{matrix} \end{matrix}\begin{matrix} 40 \\ 0 \\ \begin{matrix} 0 \\ 0 \\ \begin{matrix} 0 \\ 0 \\ \begin{matrix} 0 \\ 0 \\ \begin{matrix} 0 \\ 0 \\ \begin{matrix} 0 \\ 0 \\ \begin{matrix} 0 \\ 0 \\ \begin{matrix} 0 \\ 0 \\ \begin{matrix} 0 \\ 0 \\ \begin{matrix} 0 \\ 0 \\ \begin{matrix} 0 \\ 0 \\ \begin{matrix} 0 \\ 0 \\ \begin{matrix} 0 \\ 0 \\ \begin{matrix} 0 \\ 0 \\ \begin{matrix} 0 \\ 0 \\ \begin{matrix} 0 \\ 0 \\ \begin{matrix} 0 \\ 0 \\ \begin{matrix} 0 \\ 0 \\ \begin{matrix} 0 \\ 0 \\ \begin{matrix} 0 \\ 5.0 \\ \begin{matrix} 0 \\ 0 \\ \begin{matrix} 0 \\ 0 \\ \begin{matrix} 0 \\ \begin{matrix} 0 \\ 0 \end{matrix} \end{matrix} \end{matrix} \end{matrix} \end{matrix} \end{matrix} \end{matrix} \end{matrix} \end{matrix} \end{matrix} \end{matrix} \end{matrix} \end{matrix} \end{matrix} \end{matrix} \end{matrix} \end{matrix} \end{matrix} \end{matrix} \end{matrix} \end{matrix} \end{matrix} \end{matrix} \end{matrix}\begin{matrix} 41 \\ 0 \\ \begin{matrix} 0 \\ 0 \\ \begin{matrix} 0 \\ 0 \\ \begin{matrix} 0 \\ 0 \\ \begin{matrix} 0 \\ 0 \\ \begin{matrix} 0 \\ 0 \\ \begin{matrix} 0 \\ 0 \\ \begin{matrix} 0 \\ 0 \\ \begin{matrix} 0 \\ 0 \\ \begin{matrix} 0 \\ 0 \\ \begin{matrix} 0 \\ 24.0W_{nn} \\ \begin{matrix} 0 \\ 24.1W_{nn} \\ \begin{matrix} 5.3W_{nn} \\ 2.5W_{nn} \\ \begin{matrix} 24.0W_{nn} \\ 0 \\ \begin{matrix} 24.1W_{nn} \\ 5.3W_{nn} \\ \begin{matrix} 2.5W_{nn} \\ 0 \\ \begin{matrix} 0 \\ 0 \\ \begin{matrix} 0 \\ 0 \\ \begin{matrix} 0 \\ 0 \\ \begin{matrix} 0 \\ 0 \\ \begin{matrix} 0 \\ 0 \\ \begin{matrix} 5.0 \\ 0 \\ \begin{matrix} 0 \\ \begin{matrix} {2.5W}_{nn} \\ 4.9W_{nn} \end{matrix} \end{matrix} \end{matrix} \end{matrix} \end{matrix} \end{matrix} \end{matrix} \end{matrix} \end{matrix} \end{matrix} \end{matrix} \end{matrix} \end{matrix} \end{matrix} \end{matrix} \end{matrix} \end{matrix} \end{matrix} \end{matrix} \end{matrix} \end{matrix} \end{matrix} \end{matrix} \end{matrix}\begin{matrix} 42 \\ 0 \\ \begin{matrix} 0 \\ 0 \\ \begin{matrix} 0 \\ 0 \\ \begin{matrix} 0 \\ 0 \\ \begin{matrix} 0 \\ 0 \\ \begin{matrix} 0 \\ 0 \\ \begin{matrix} 0 \\ 0 \\ \begin{matrix} 0 \\ 0 \\ \begin{matrix} 0 \\ 0 \\ \begin{matrix} 0 \\ 0 \\ \begin{matrix} 0 \\ 0 \\ \begin{matrix} 0 \\ 0 \\ \begin{matrix} 0 \\ 0 \\ \begin{matrix} 0 \\ 0 \\ \begin{matrix} 0 \\ 0 \\ \begin{matrix} 0 \\ 0 \\ \begin{matrix} 0 \\ 0 \\ \begin{matrix} 0 \\ 0 \\ \begin{matrix} 0 \\ 0 \\ \begin{matrix} 0 \\ 0 \\ \begin{matrix} 0 \\ 5.0 \\ \begin{matrix} 0 \\ 0 \\ \begin{matrix} 0 \\ \begin{matrix} 0 \\ 0 \end{matrix} \end{matrix} \end{matrix} \end{matrix} \end{matrix} \end{matrix} \end{matrix} \end{matrix} \end{matrix} \end{matrix} \end{matrix} \end{matrix} \end{matrix} \end{matrix} \end{matrix} \end{matrix} \end{matrix} \end{matrix} \end{matrix} \end{matrix} \end{matrix} \end{matrix} \end{matrix} \end{matrix}\begin{matrix} 43 \\ \begin{matrix} 13.0W_{d} \\ 0 \\ \begin{matrix} 6.9W_{d} \\ 1.5W_{d} \\ \begin{matrix} 0.7W_{d} \\ 13.0W_{d} \\ \begin{matrix} 0 \\ 6.9W_{d} \\ \begin{matrix} 1.5W_{d} \\ 0.7W_{d} \\ \begin{matrix} 13.0W_{d} \\ 0 \\ \begin{matrix} 6.9W_{d} \\ 1.5W_{d} \\ \begin{matrix} 0.7W_{d} \\ 13.0W_{d} \\ \begin{matrix} 0 \\ 6.9W_{d} \\ \begin{matrix} 1.5W_{d} \\ 0.7W_{d} \\ \begin{matrix} 13.0W_{d} \\ 0 \\ \begin{matrix} 6.9W_{d} \\ 1.5W_{d} \\ \begin{matrix} 0.7W_{d} \\ 13.0W_{d} \\ \begin{matrix} 0 \\ 6.9W_{d} \\ \begin{matrix} 1.5W_{d} \\ 0.7W_{d} \\ \begin{matrix} 0 \\ 0 \\ \begin{matrix} 0 \\ 0 \\ \begin{matrix} 0 \\ 0 \\ \begin{matrix} 0 \\ 0 \\ \begin{matrix} 0 \\ 0 \\ \begin{matrix} 0 \\ 0 \\ \begin{matrix} 0 \\ 5.0 \\ \begin{matrix} {0.7W}_{d} \\ 1.4W_{d} \end{matrix} \end{matrix} \end{matrix} \end{matrix} \end{matrix} \end{matrix} \end{matrix} \end{matrix} \end{matrix} \end{matrix} \end{matrix} \end{matrix} \end{matrix} \end{matrix} \end{matrix} \end{matrix} \end{matrix} \end{matrix} \end{matrix} \end{matrix} \end{matrix} \end{matrix} \end{matrix} \end{matrix}\begin{matrix} 44 \\ 0 \\ \begin{matrix} 0 \\ 0 \\ \begin{matrix} 0 \\ 0 \\ \begin{matrix} 0 \\ 0 \\ \begin{matrix} 0 \\ 0 \\ \begin{matrix} 0 \\ 0 \\ \begin{matrix} 0 \\ 0 \\ \begin{matrix} 0 \\ 0 \\ \begin{matrix} 0 \\ 0 \\ \begin{matrix} 0 \\ 0 \\ \begin{matrix} 0 \\ 0 \\ \begin{matrix} 0 \\ 0 \\ \begin{matrix} 0 \\ 0 \\ \begin{matrix} 0 \\ 0 \\ \begin{matrix} 0 \\ 0 \\ \begin{matrix} 0 \\ 0 \\ \begin{matrix} 0 \\ 0 \\ \begin{matrix} 0 \\ 0 \\ \begin{matrix} 0 \\ 0 \\ \begin{matrix} 0 \\ 0 \\ \begin{matrix} 0 \\ 0 \\ \begin{matrix} 0 \\ 5.0 \\ \begin{matrix} 0 \\ \begin{matrix} 0 \\ 0 \end{matrix} \end{matrix} \end{matrix} \end{matrix} \end{matrix} \end{matrix} \end{matrix} \end{matrix} \end{matrix} \end{matrix} \end{matrix} \end{matrix} \end{matrix} \end{matrix} \end{matrix} \end{matrix} \end{matrix} \end{matrix} \end{matrix} \end{matrix} \end{matrix} \end{matrix} \end{matrix} \end{matrix}\begin{matrix} 45 \\ 0.5 \\ \begin{matrix} 0 \\ 0 \\ \begin{matrix} 0 \\ 0 \\ \begin{matrix} 0.5 \\ 0 \\ \begin{matrix} 0 \\ 0 \\ \begin{matrix} 0 \\ 0.5 \\ \begin{matrix} 0 \\ 0 \\ \begin{matrix} 0 \\ 0 \\ \begin{matrix} 0.5 \\ 0 \\ \begin{matrix} 0 \\ 0 \\ \begin{matrix} 0 \\ 0.5 \\ \begin{matrix} 0 \\ 0 \\ \begin{matrix} 0 \\ 0 \\ \begin{matrix} 0.5 \\ 0 \\ \begin{matrix} 0 \\ 0 \\ \begin{matrix} 0 \\ {2.5W}_{dn} \\ \begin{matrix} 0 \\ {2.5W}_{nn} \\ \begin{matrix} 0 \\ {2.5W}_{dn} \\ \begin{matrix} 0 \\ {2.5W}_{nn} \\ \begin{matrix} 0 \\ {2.5W}_{dn} \\ \begin{matrix} 0 \\ {2.5W}_{nn} \\ \begin{matrix} 0 \\ {2.5W}_{d} \\ \begin{matrix} 0 \\ \begin{matrix} 0 \\ 0 \end{matrix} \end{matrix} \end{matrix} \end{matrix} \end{matrix} \end{matrix} \end{matrix} \end{matrix} \end{matrix} \end{matrix} \end{matrix} \end{matrix} \end{matrix} \end{matrix} \end{matrix} \end{matrix} \end{matrix} \end{matrix} \end{matrix} \end{matrix} \end{matrix} \end{matrix} \end{matrix} \end{matrix}\begin{matrix} 46 \\ 0 \\ \begin{matrix} 0 \\ 0 \\ \begin{matrix} 0 \\ 0 \\ \begin{matrix} 0 \\ 0 \\ \begin{matrix} 0 \\ 0 \\ \begin{matrix} 0 \\ 0 \\ \begin{matrix} 0 \\ 0 \\ \begin{matrix} 0 \\ 0 \\ \begin{matrix} 0 \\ 0 \\ \begin{matrix} 0 \\ 0 \\ \begin{matrix} 0 \\ 0 \\ \begin{matrix} 0 \\ 0 \\ \begin{matrix} 0 \\ 0 \\ \begin{matrix} 0 \\ 0 \\ \begin{matrix} 0 \\ 0 \\ \begin{matrix} 0 \\ 4.9W_{dn} \\ \begin{matrix} 0 \\ 4.9W_{nn} \\ \begin{matrix} 0 \\ 4.9W_{dn} \\ \begin{matrix} 0 \\ 4.9W_{nn} \\ \begin{matrix} 0 \\ 4.9W_{dn} \\ \begin{matrix} 0 \\ 4.9W_{nn} \\ \begin{matrix} 0 \\ 4.9W_{d} \\ \begin{matrix} 0 \\ \begin{matrix} 0 \\ 0 \end{matrix} \end{matrix} \end{matrix} \end{matrix} \end{matrix} \end{matrix} \end{matrix} \end{matrix} \end{matrix} \end{matrix} \end{matrix} \end{matrix} \end{matrix} \end{matrix} \end{matrix} \end{matrix} \end{matrix} \end{matrix} \end{matrix} \end{matrix} \end{matrix} \end{matrix} \end{matrix} \end{matrix} \right]$$

Pathogen transfer efficiency matrix ($\tau={{(\tau}_{ij})}_{46\times46}$), (since pathogen transfer only occurs during hand contact, so the transfer efficiency between the two compartments there are contact between them are provided）

$$\tau=$$

$$\begin{matrix} \\ \begin{matrix} 1 \\ 2 \\ \begin{matrix} 3 \\ 4 \\ \begin{matrix} 5 \\ 6 \\ \begin{matrix} 7 \\ 8 \\ \begin{matrix} 9 \\ 10 \\ \begin{matrix} 11 \\ 12 \\ \begin{matrix} 13 \\ 14 \\ \begin{matrix} 15 \\ 16 \\ \begin{matrix} 17 \\ 18 \\ \begin{matrix} 19 \\ 20 \\ \begin{matrix} 21 \\ 22 \\ \begin{matrix} 23 \\ 24 \\ \begin{matrix} 25 \\ 26 \\ \begin{matrix} 27 \\ 28 \\ \begin{matrix} 29 \\ 30 \\ \begin{matrix} 31 \\ 32 \\ \begin{matrix} 33 \\ 34 \\ \begin{matrix} 35 \\ 36 \\ \begin{matrix} 37 \\ 38 \\ \begin{matrix} 39 \\ 40 \\ \begin{matrix} 41 \\ 42 \\ \begin{matrix} 43 \\ 44 \\ \begin{matrix} \begin{matrix} 45 \\ 46 \end{matrix} \end{matrix} \end{matrix} \end{matrix} \end{matrix} \end{matrix} \end{matrix} \end{matrix} \end{matrix} \end{matrix} \end{matrix} \end{matrix} \end{matrix} \end{matrix} \end{matrix} \end{matrix} \end{matrix} \end{matrix} \end{matrix} \end{matrix} \end{matrix} \end{matrix} \end{matrix} \end{matrix} \end{matrix}\left[ \begin{matrix} 1 \\ \begin{matrix} 0 \\ 0.35 \\ \begin{matrix} 0.23 \\ 0.23 \\ \begin{matrix} 0.23 \\ 0 \\ \begin{matrix} 0 \\ 0 \\ \begin{matrix} 0 \\ 0 \\ \begin{matrix} 0 \\ 0 \\ \begin{matrix} 0 \\ 0 \\ \begin{matrix} 0 \\ 0 \\ \begin{matrix} 0 \\ 0 \\ \begin{matrix} 0 \\ 0 \\ \begin{matrix} 0 \\ 0 \\ \begin{matrix} 0 \\ 0 \\ \begin{matrix} 0 \\ 0 \\ \begin{matrix} 0 \\ 0 \\ \begin{matrix} 0 \\ 0 \\ \begin{matrix} 0.35 \\ 0 \\ \begin{matrix} 0.35 \\ 0 \\ \begin{matrix} 0 \\ 0 \\ \begin{matrix} 0 \\ 0 \\ \begin{matrix} 0 \\ 0 \\ \begin{matrix} 0 \\ 0 \\ \begin{matrix} 0.35 \\ 0 \\ \begin{matrix} 0.23 \\ 0 \end{matrix} \end{matrix} \end{matrix} \end{matrix} \end{matrix} \end{matrix} \end{matrix} \end{matrix} \end{matrix} \end{matrix} \end{matrix} \end{matrix} \end{matrix} \end{matrix} \end{matrix} \end{matrix} \end{matrix} \end{matrix} \end{matrix} \end{matrix} \end{matrix} \end{matrix} \end{matrix} \end{matrix}\begin{matrix} 2 \\ 0.2 \\ \begin{matrix} 0 \\ 0 \\ \begin{matrix} 0 \\ 0 \\ \begin{matrix} 0 \\ 0 \\ \begin{matrix} 0 \\ 0 \\ \begin{matrix} 0 \\ 0 \\ \begin{matrix} 0 \\ 0 \\ \begin{matrix} 0 \\ 0 \\ \begin{matrix} 0 \\ 0 \\ \begin{matrix} 0 \\ 0 \\ \begin{matrix} 0 \\ 0 \\ \begin{matrix} 0 \\ 0 \\ \begin{matrix} 0 \\ 0 \\ \begin{matrix} 0 \\ 0 \\ \begin{matrix} 0 \\ 0 \\ \begin{matrix} 0 \\ 0 \\ \begin{matrix} 0 \\ 0 \\ \begin{matrix} 0 \\ 0 \\ \begin{matrix} 0 \\ 0 \\ \begin{matrix} 0 \\ 0 \\ \begin{matrix} 0 \\ 0 \\ \begin{matrix} 0 \\ 0 \\ \begin{matrix} 0 \\ \begin{matrix} 0 \\ 0 \end{matrix} \end{matrix} \end{matrix} \end{matrix} \end{matrix} \end{matrix} \end{matrix} \end{matrix} \end{matrix} \end{matrix} \end{matrix} \end{matrix} \end{matrix} \end{matrix} \end{matrix} \end{matrix} \end{matrix} \end{matrix} \end{matrix} \end{matrix} \end{matrix} \end{matrix} \end{matrix} \end{matrix}\begin{matrix} 3 \\ 0.28 \\ \begin{matrix} 0 \\ 0 \\ \begin{matrix} 0 \\ 0 \\ \begin{matrix} 0 \\ 0 \\ \begin{matrix} 0 \\ 0 \\ \begin{matrix} 0 \\ 0 \\ \begin{matrix} 0 \\ 0 \\ \begin{matrix} 0 \\ 0 \\ \begin{matrix} 0 \\ 0 \\ \begin{matrix} 0 \\ 0 \\ \begin{matrix} 0 \\ 0 \\ \begin{matrix} 0 \\ 0 \\ \begin{matrix} 0 \\ 0 \\ \begin{matrix} 0 \\ 0 \\ \begin{matrix} 0 \\ 0 \\ \begin{matrix} 0 \\ 0.28 \\ \begin{matrix} 0 \\ 0.28 \\ \begin{matrix} 0 \\ 0 \\ \begin{matrix} 0 \\ 0 \\ \begin{matrix} 0 \\ 0 \\ \begin{matrix} 0 \\ 0 \\ \begin{matrix} 0 \\ 0.28 \\ \begin{matrix} 0 \\ \begin{matrix} 0 \\ 0 \end{matrix} \end{matrix} \end{matrix} \end{matrix} \end{matrix} \end{matrix} \end{matrix} \end{matrix} \end{matrix} \end{matrix} \end{matrix} \end{matrix} \end{matrix} \end{matrix} \end{matrix} \end{matrix} \end{matrix} \end{matrix} \end{matrix} \end{matrix} \end{matrix} \end{matrix} \end{matrix} \end{matrix}\begin{matrix} 4 \\ 0.28 \\ \begin{matrix} 0 \\ 0 \\ \begin{matrix} 0 \\ 0 \\ \begin{matrix} 0 \\ 0 \\ \begin{matrix} 0 \\ 0 \\ \begin{matrix} 0 \\ 0 \\ \begin{matrix} 0 \\ 0 \\ \begin{matrix} 0 \\ 0 \\ \begin{matrix} 0 \\ 0 \\ \begin{matrix} 0 \\ 0 \\ \begin{matrix} 0 \\ 0 \\ \begin{matrix} 0 \\ 0 \\ \begin{matrix} 0 \\ 0 \\ \begin{matrix} 0 \\ 0 \\ \begin{matrix} 0 \\ 0 \\ \begin{matrix} 0 \\ 0.28 \\ \begin{matrix} 0 \\ 0.28 \\ \begin{matrix} 0 \\ 0 \\ \begin{matrix} 0 \\ 0 \\ \begin{matrix} 0 \\ 0 \\ \begin{matrix} 0 \\ 0 \\ \begin{matrix} 0 \\ 0.28 \\ \begin{matrix} 0 \\ \begin{matrix} 0 \\ 0 \end{matrix} \end{matrix} \end{matrix} \end{matrix} \end{matrix} \end{matrix} \end{matrix} \end{matrix} \end{matrix} \end{matrix} \end{matrix} \end{matrix} \end{matrix} \end{matrix} \end{matrix} \end{matrix} \end{matrix} \end{matrix} \end{matrix} \end{matrix} \end{matrix} \end{matrix} \end{matrix} \end{matrix}\begin{matrix} 5 \\ 0.28 \\ \begin{matrix} 0 \\ 0 \\ \begin{matrix} 0 \\ 0 \\ \begin{matrix} 0 \\ 0 \\ \begin{matrix} 0 \\ 0 \\ \begin{matrix} 0 \\ 0 \\ \begin{matrix} 0 \\ 0 \\ \begin{matrix} 0 \\ 0 \\ \begin{matrix} 0 \\ 0 \\ \begin{matrix} 0 \\ 0 \\ \begin{matrix} 0 \\ 0 \\ \begin{matrix} 0 \\ 0 \\ \begin{matrix} 0 \\ 0 \\ \begin{matrix} 0 \\ 0 \\ \begin{matrix} 0 \\ 0 \\ \begin{matrix} 0 \\ 0.28 \\ \begin{matrix} 0 \\ 0.28 \\ \begin{matrix} 0 \\ 0 \\ \begin{matrix} 0 \\ 0 \\ \begin{matrix} 0 \\ 0 \\ \begin{matrix} 0 \\ 0 \\ \begin{matrix} 0 \\ 0.28 \\ \begin{matrix} 0 \\ \begin{matrix} 0 \\ 0 \end{matrix} \end{matrix} \end{matrix} \end{matrix} \end{matrix} \end{matrix} \end{matrix} \end{matrix} \end{matrix} \end{matrix} \end{matrix} \end{matrix} \end{matrix} \end{matrix} \end{matrix} \end{matrix} \end{matrix} \end{matrix} \end{matrix} \end{matrix} \end{matrix} \end{matrix} \end{matrix} \end{matrix}\begin{matrix} 6 \\ 0 \\ \begin{matrix} 0 \\ 0 \\ \begin{matrix} 0 \\ 0 \\ \begin{matrix} 0 \\ 0.35 \\ \begin{matrix} 0.23 \\ 0.23 \\ \begin{matrix} 0.23 \\ 0 \\ \begin{matrix} 0 \\ 0 \\ \begin{matrix} 0 \\ 0 \\ \begin{matrix} 0 \\ 0 \\ \begin{matrix} 0 \\ 0 \\ \begin{matrix} 0 \\ 0 \\ \begin{matrix} 0 \\ 0 \\ \begin{matrix} 0 \\ 0 \\ \begin{matrix} 0 \\ 0 \\ \begin{matrix} 0 \\ 0 \\ \begin{matrix} 0 \\ 0.35 \\ \begin{matrix} 0 \\ 0.35 \\ \begin{matrix} 0 \\ 0 \\ \begin{matrix} 0 \\ 0 \\ \begin{matrix} 0 \\ 0 \\ \begin{matrix} 0 \\ 0 \\ \begin{matrix} 0 \\ 0.35 \\ \begin{matrix} 0 \\ \begin{matrix} 0.23 \\ 0 \end{matrix} \end{matrix} \end{matrix} \end{matrix} \end{matrix} \end{matrix} \end{matrix} \end{matrix} \end{matrix} \end{matrix} \end{matrix} \end{matrix} \end{matrix} \end{matrix} \end{matrix} \end{matrix} \end{matrix} \end{matrix} \end{matrix} \end{matrix} \end{matrix} \end{matrix} \end{matrix} \end{matrix}\begin{matrix} 7 \\ 0 \\ \begin{matrix} 0 \\ 0 \\ \begin{matrix} 0 \\ 0 \\ \begin{matrix} 0.2 \\ 0 \\ \begin{matrix} 0 \\ 0 \\ \begin{matrix} 0 \\ 0 \\ \begin{matrix} 0 \\ 0 \\ \begin{matrix} 0 \\ 0 \\ \begin{matrix} 0 \\ 0 \\ \begin{matrix} 0 \\ 0 \\ \begin{matrix} 0 \\ 0 \\ \begin{matrix} 0 \\ 0 \\ \begin{matrix} 0 \\ 0 \\ \begin{matrix} 0 \\ 0 \\ \begin{matrix} 0 \\ 0 \\ \begin{matrix} 0 \\ 0 \\ \begin{matrix} 0 \\ 0 \\ \begin{matrix} 0 \\ 0 \\ \begin{matrix} 0 \\ 0 \\ \begin{matrix} 0 \\ 0 \\ \begin{matrix} 0 \\ 0 \\ \begin{matrix} 0 \\ 0 \\ \begin{matrix} 0 \\ \begin{matrix} 0 \\ 0 \end{matrix} \end{matrix} \end{matrix} \end{matrix} \end{matrix} \end{matrix} \end{matrix} \end{matrix} \end{matrix} \end{matrix} \end{matrix} \end{matrix} \end{matrix} \end{matrix} \end{matrix} \end{matrix} \end{matrix} \end{matrix} \end{matrix} \end{matrix} \end{matrix} \end{matrix} \end{matrix} \end{matrix}\begin{matrix} 8 \\ 0 \\ \begin{matrix} 0 \\ 0 \\ \begin{matrix} 0 \\ 0 \\ \begin{matrix} 0.28 \\ 0 \\ \begin{matrix} 0 \\ 0 \\ \begin{matrix} 0 \\ 0 \\ \begin{matrix} 0 \\ 0 \\ \begin{matrix} 0 \\ 0 \\ \begin{matrix} 0 \\ 0 \\ \begin{matrix} 0 \\ 0 \\ \begin{matrix} 0 \\ 0 \\ \begin{matrix} 0 \\ 0 \\ \begin{matrix} 0 \\ 0 \\ \begin{matrix} 0 \\ 0 \\ \begin{matrix} 0 \\ 0 \\ \begin{matrix} 0 \\ 0.35 \\ \begin{matrix} 0 \\ 0.35 \\ \begin{matrix} 0 \\ 0 \\ \begin{matrix} 0 \\ 0 \\ \begin{matrix} 0 \\ 0 \\ \begin{matrix} 0 \\ 0 \\ \begin{matrix} 0 \\ 0.35 \\ \begin{matrix} 0 \\ \begin{matrix} 0 \\ 0 \end{matrix} \end{matrix} \end{matrix} \end{matrix} \end{matrix} \end{matrix} \end{matrix} \end{matrix} \end{matrix} \end{matrix} \end{matrix} \end{matrix} \end{matrix} \end{matrix} \end{matrix} \end{matrix} \end{matrix} \end{matrix} \end{matrix} \end{matrix} \end{matrix} \end{matrix} \end{matrix} \end{matrix}\begin{matrix} 9 \\ 0 \\ \begin{matrix} 0 \\ 0 \\ \begin{matrix} 0 \\ 0 \\ \begin{matrix} 0.28 \\ 0 \\ \begin{matrix} 0 \\ 0 \\ \begin{matrix} 0 \\ 0 \\ \begin{matrix} 0 \\ 0 \\ \begin{matrix} 0 \\ 0 \\ \begin{matrix} 0 \\ 0 \\ \begin{matrix} 0 \\ 0 \\ \begin{matrix} 0 \\ 0 \\ \begin{matrix} 0 \\ 0 \\ \begin{matrix} 0 \\ 0 \\ \begin{matrix} 0 \\ 0 \\ \begin{matrix} 0 \\ 0 \\ \begin{matrix} 0 \\ 0.28 \\ \begin{matrix} 0 \\ 0.28 \\ \begin{matrix} 0 \\ 0 \\ \begin{matrix} 0 \\ 0 \\ \begin{matrix} 0 \\ 0 \\ \begin{matrix} 0 \\ 0 \\ \begin{matrix} 0 \\ 0.28 \\ \begin{matrix} 0 \\ \begin{matrix} 0 \\ 0 \end{matrix} \end{matrix} \end{matrix} \end{matrix} \end{matrix} \end{matrix} \end{matrix} \end{matrix} \end{matrix} \end{matrix} \end{matrix} \end{matrix} \end{matrix} \end{matrix} \end{matrix} \end{matrix} \end{matrix} \end{matrix} \end{matrix} \end{matrix} \end{matrix} \end{matrix} \end{matrix} \end{matrix}\begin{matrix} 10 \\ 0 \\ \begin{matrix} 0 \\ 0 \\ \begin{matrix} 0 \\ 0 \\ \begin{matrix} 0.28 \\ 0 \\ \begin{matrix} 0 \\ 0 \\ \begin{matrix} 0 \\ 0 \\ \begin{matrix} 0 \\ 0 \\ \begin{matrix} 0 \\ 0 \\ \begin{matrix} 0 \\ 0 \\ \begin{matrix} 0 \\ 0 \\ \begin{matrix} 0 \\ 0 \\ \begin{matrix} 0 \\ 0 \\ \begin{matrix} 0 \\ 0 \\ \begin{matrix} 0 \\ 0 \\ \begin{matrix} 0 \\ 0 \\ \begin{matrix} 0 \\ 0.28 \\ \begin{matrix} 0 \\ 0.28 \\ \begin{matrix} 0 \\ 0 \\ \begin{matrix} 0 \\ 0 \\ \begin{matrix} 0 \\ 0 \\ \begin{matrix} 0 \\ 0 \\ \begin{matrix} 0 \\ 0.28 \\ \begin{matrix} 0 \\ \begin{matrix} 0 \\ 0 \end{matrix} \end{matrix} \end{matrix} \end{matrix} \end{matrix} \end{matrix} \end{matrix} \end{matrix} \end{matrix} \end{matrix} \end{matrix} \end{matrix} \end{matrix} \end{matrix} \end{matrix} \end{matrix} \end{matrix} \end{matrix} \end{matrix} \end{matrix} \end{matrix} \end{matrix} \end{matrix} \end{matrix}\begin{matrix} 11 \\ \begin{matrix} 0 \\ 0 \\ \begin{matrix} 0 \\ 0 \\ \begin{matrix} 0 \\ 0 \\ \begin{matrix} 0 \\ 0 \\ \begin{matrix} 0 \\ 0 \\ \begin{matrix} 0 \\ 0.35 \\ \begin{matrix} 0.23 \\ 0.23 \\ \begin{matrix} 0.23 \\ 0 \\ \begin{matrix} 0 \\ 0 \\ \begin{matrix} 0 \\ 0 \\ \begin{matrix} 0 \\ 0 \\ \begin{matrix} 0 \\ 0 \\ \begin{matrix} 0 \\ 0 \\ \begin{matrix} 0 \\ 0 \\ \begin{matrix} 0 \\ 0 \\ \begin{matrix} 0 \\ 0 \\ \begin{matrix} 0 \\ 0 \\ \begin{matrix} 0.35 \\ 0 \\ \begin{matrix} 0.35 \\ 0 \\ \begin{matrix} 0 \\ 0 \\ \begin{matrix} 0 \\ 0 \\ \begin{matrix} 0.35 \\ 0 \\ \begin{matrix} 0.23 \\ 0 \end{matrix} \end{matrix} \end{matrix} \end{matrix} \end{matrix} \end{matrix} \end{matrix} \end{matrix} \end{matrix} \end{matrix} \end{matrix} \end{matrix} \end{matrix} \end{matrix} \end{matrix} \end{matrix} \end{matrix} \end{matrix} \end{matrix} \end{matrix} \end{matrix} \end{matrix} \end{matrix} \end{matrix}\begin{matrix} 12 \\ 0 \\ \begin{matrix} 0 \\ 0 \\ \begin{matrix} 0 \\ 0 \\ \begin{matrix} 0 \\ 0 \\ \begin{matrix} 0 \\ 0 \\ \begin{matrix} 0 \\ 0.2 \\ \begin{matrix} 0 \\ 0 \\ \begin{matrix} 0 \\ 0 \\ \begin{matrix} 0 \\ 0 \\ \begin{matrix} 0 \\ 0 \\ \begin{matrix} 0 \\ 0 \\ \begin{matrix} 0 \\ 0 \\ \begin{matrix} 0 \\ 0 \\ \begin{matrix} 0 \\ 0 \\ \begin{matrix} 0 \\ 0 \\ \begin{matrix} 0 \\ 0 \\ \begin{matrix} 0 \\ 0 \\ \begin{matrix} 0 \\ 0 \\ \begin{matrix} 0 \\ 0 \\ \begin{matrix} 0 \\ 0 \\ \begin{matrix} 0 \\ 0 \\ \begin{matrix} 0 \\ 0 \\ \begin{matrix} 0 \\ \begin{matrix} 0 \\ 0 \end{matrix} \end{matrix} \end{matrix} \end{matrix} \end{matrix} \end{matrix} \end{matrix} \end{matrix} \end{matrix} \end{matrix} \end{matrix} \end{matrix} \end{matrix} \end{matrix} \end{matrix} \end{matrix} \end{matrix} \end{matrix} \end{matrix} \end{matrix} \end{matrix} \end{matrix} \end{matrix} \end{matrix}\begin{matrix} 13 \\ 0 \\ \begin{matrix} 0 \\ 0 \\ \begin{matrix} 0 \\ 0 \\ \begin{matrix} 0 \\ 0 \\ \begin{matrix} 0 \\ 0 \\ \begin{matrix} 0 \\ 0.28 \\ \begin{matrix} 0 \\ 0 \\ \begin{matrix} 0 \\ 0 \\ \begin{matrix} 0 \\ 0 \\ \begin{matrix} 0 \\ 0 \\ \begin{matrix} 0 \\ 0 \\ \begin{matrix} 0 \\ 0 \\ \begin{matrix} 0 \\ 0 \\ \begin{matrix} 0 \\ 0 \\ \begin{matrix} 0 \\ 0 \\ \begin{matrix} 0 \\ 0 \\ \begin{matrix} 0 \\ 0 \\ \begin{matrix} 0 \\ 0.28 \\ \begin{matrix} 0 \\ 0.28 \\ \begin{matrix} 0 \\ 0 \\ \begin{matrix} 0 \\ 0 \\ \begin{matrix} 0 \\ 0.28 \\ \begin{matrix} 0 \\ \begin{matrix} 0 \\ 0 \end{matrix} \end{matrix} \end{matrix} \end{matrix} \end{matrix} \end{matrix} \end{matrix} \end{matrix} \end{matrix} \end{matrix} \end{matrix} \end{matrix} \end{matrix} \end{matrix} \end{matrix} \end{matrix} \end{matrix} \end{matrix} \end{matrix} \end{matrix} \end{matrix} \end{matrix} \end{matrix} \end{matrix}\begin{matrix} 14 \\ 0 \\ \begin{matrix} 0 \\ 0 \\ \begin{matrix} 0 \\ 0 \\ \begin{matrix} 0 \\ 0 \\ \begin{matrix} 0 \\ 0 \\ \begin{matrix} 0 \\ 0.28 \\ \begin{matrix} 0 \\ 0 \\ \begin{matrix} 0 \\ 0 \\ \begin{matrix} 0 \\ 0 \\ \begin{matrix} 0 \\ 0 \\ \begin{matrix} 0 \\ 0 \\ \begin{matrix} 0 \\ 0 \\ \begin{matrix} 0 \\ 0 \\ \begin{matrix} 0 \\ 0 \\ \begin{matrix} 0 \\ 0 \\ \begin{matrix} 0 \\ 0 \\ \begin{matrix} 0 \\ 0 \\ \begin{matrix} 0 \\ 0.28 \\ \begin{matrix} 0 \\ 0.28 \\ \begin{matrix} 0 \\ 0 \\ \begin{matrix} 0 \\ 0 \\ \begin{matrix} 0 \\ 0.28 \\ \begin{matrix} 0 \\ \begin{matrix} 0 \\ 0 \end{matrix} \end{matrix} \end{matrix} \end{matrix} \end{matrix} \end{matrix} \end{matrix} \end{matrix} \end{matrix} \end{matrix} \end{matrix} \end{matrix} \end{matrix} \end{matrix} \end{matrix} \end{matrix} \end{matrix} \end{matrix} \end{matrix} \end{matrix} \end{matrix} \end{matrix} \end{matrix} \end{matrix}\begin{matrix} 15 \\ 0 \\ \begin{matrix} 0 \\ 0 \\ \begin{matrix} 0 \\ 0 \\ \begin{matrix} 0 \\ 0 \\ \begin{matrix} 0 \\ 0 \\ \begin{matrix} 0 \\ 0.28 \\ \begin{matrix} 0 \\ 0 \\ \begin{matrix} 0 \\ 0 \\ \begin{matrix} 0 \\ 0 \\ \begin{matrix} 0 \\ 0 \\ \begin{matrix} 0 \\ 0 \\ \begin{matrix} 0 \\ 0 \\ \begin{matrix} 0 \\ 0 \\ \begin{matrix} 0 \\ 0 \\ \begin{matrix} 0 \\ 0 \\ \begin{matrix} 0 \\ 0 \\ \begin{matrix} 0 \\ 0 \\ \begin{matrix} 0 \\ 0.28 \\ \begin{matrix} 0 \\ 0.28 \\ \begin{matrix} 0 \\ 0 \\ \begin{matrix} 0 \\ 0 \\ \begin{matrix} 0 \\ 0.28 \\ \begin{matrix} 0 \\ \begin{matrix} 0 \\ 0 \end{matrix} \end{matrix} \end{matrix} \end{matrix} \end{matrix} \end{matrix} \end{matrix} \end{matrix} \end{matrix} \end{matrix} \end{matrix} \end{matrix} \end{matrix} \end{matrix} \end{matrix} \end{matrix} \end{matrix} \end{matrix} \end{matrix} \end{matrix} \end{matrix} \end{matrix} \end{matrix} \end{matrix}\begin{matrix} 16 \\ \begin{matrix} 0 \\ 0 \\ \begin{matrix} 0 \\ 0 \\ \begin{matrix} 0 \\ 0 \\ \begin{matrix} 0 \\ 0 \\ \begin{matrix} 0 \\ 0 \\ \begin{matrix} 0 \\ 0 \\ \begin{matrix} 0 \\ 0 \\ \begin{matrix} 0 \\ 0 \\ \begin{matrix} 0.35 \\ 0.23 \\ \begin{matrix} 0.23 \\ 0.23 \\ \begin{matrix} 0 \\ 0 \\ \begin{matrix} 0 \\ 0 \\ \begin{matrix} 0 \\ 0 \\ \begin{matrix} 0 \\ 0 \\ \begin{matrix} 0 \\ 0 \\ \begin{matrix} 0 \\ 0 \\ \begin{matrix} 0 \\ 0 \\ \begin{matrix} 0.35 \\ 0 \\ \begin{matrix} 0.35 \\ 0 \\ \begin{matrix} 0 \\ 0 \\ \begin{matrix} 0 \\ 0 \\ \begin{matrix} 0.35 \\ 0 \\ \begin{matrix} 0.23 \\ 0 \end{matrix} \end{matrix} \end{matrix} \end{matrix} \end{matrix} \end{matrix} \end{matrix} \end{matrix} \end{matrix} \end{matrix} \end{matrix} \end{matrix} \end{matrix} \end{matrix} \end{matrix} \end{matrix} \end{matrix} \end{matrix} \end{matrix} \end{matrix} \end{matrix} \end{matrix} \end{matrix} \end{matrix}\begin{matrix} 17 \\ 0 \\ \begin{matrix} 0 \\ 0 \\ \begin{matrix} 0 \\ 0 \\ \begin{matrix} 0 \\ 0 \\ \begin{matrix} 0 \\ 0 \\ \begin{matrix} 0 \\ 0 \\ \begin{matrix} 0 \\ 0 \\ \begin{matrix} 0 \\ 0 \\ \begin{matrix} 0.2 \\ 0 \\ \begin{matrix} 0 \\ 0 \\ \begin{matrix} 0 \\ 0 \\ \begin{matrix} 0 \\ 0 \\ \begin{matrix} 0 \\ 0 \\ \begin{matrix} 0 \\ 0 \\ \begin{matrix} 0 \\ 0 \\ \begin{matrix} 0 \\ 0 \\ \begin{matrix} 0 \\ 0 \\ \begin{matrix} 0 \\ 0 \\ \begin{matrix} 0 \\ 0 \\ \begin{matrix} 0 \\ 0 \\ \begin{matrix} 0 \\ 0 \\ \begin{matrix} 0 \\ 0 \\ \begin{matrix} 0 \\ \begin{matrix} 0 \\ 0 \end{matrix} \end{matrix} \end{matrix} \end{matrix} \end{matrix} \end{matrix} \end{matrix} \end{matrix} \end{matrix} \end{matrix} \end{matrix} \end{matrix} \end{matrix} \end{matrix} \end{matrix} \end{matrix} \end{matrix} \end{matrix} \end{matrix} \end{matrix} \end{matrix} \end{matrix} \end{matrix} \end{matrix}\begin{matrix} 18 \\ 0 \\ \begin{matrix} 0 \\ 0 \\ \begin{matrix} 0 \\ 0 \\ \begin{matrix} 0 \\ 0 \\ \begin{matrix} 0 \\ 0 \\ \begin{matrix} 0 \\ 0 \\ \begin{matrix} 0 \\ 0 \\ \begin{matrix} 0 \\ 0 \\ \begin{matrix} 0.28 \\ 0 \\ \begin{matrix} 0 \\ 0 \\ \begin{matrix} 0 \\ 0 \\ \begin{matrix} 0 \\ 0 \\ \begin{matrix} 0 \\ 0 \\ \begin{matrix} 0 \\ 0 \\ \begin{matrix} 0 \\ 0 \\ \begin{matrix} 0 \\ 0 \\ \begin{matrix} 0 \\ 0 \\ \begin{matrix} 0 \\ 0.28 \\ \begin{matrix} 0 \\ 0.28 \\ \begin{matrix} 0 \\ 0 \\ \begin{matrix} 0 \\ 0 \\ \begin{matrix} 0 \\ 0.28 \\ \begin{matrix} 0 \\ \begin{matrix} 0 \\ 0 \end{matrix} \end{matrix} \end{matrix} \end{matrix} \end{matrix} \end{matrix} \end{matrix} \end{matrix} \end{matrix} \end{matrix} \end{matrix} \end{matrix} \end{matrix} \end{matrix} \end{matrix} \end{matrix} \end{matrix} \end{matrix} \end{matrix} \end{matrix} \end{matrix} \end{matrix} \end{matrix} \end{matrix}\begin{matrix} 19 \\ 0 \\ \begin{matrix} 0 \\ 0 \\ \begin{matrix} 0 \\ 0 \\ \begin{matrix} 0 \\ 0 \\ \begin{matrix} 0 \\ 0 \\ \begin{matrix} 0 \\ 0 \\ \begin{matrix} 0 \\ 0 \\ \begin{matrix} 0 \\ 0 \\ \begin{matrix} 0.28 \\ 0 \\ \begin{matrix} 0 \\ 0 \\ \begin{matrix} 0 \\ 0 \\ \begin{matrix} 0 \\ 0 \\ \begin{matrix} 0 \\ 0 \\ \begin{matrix} 0 \\ 0 \\ \begin{matrix} 0 \\ 0 \\ \begin{matrix} 0 \\ 0 \\ \begin{matrix} 0 \\ 0 \\ \begin{matrix} 0 \\ 0.28 \\ \begin{matrix} 0 \\ 0.28 \\ \begin{matrix} 0 \\ 0 \\ \begin{matrix} 0 \\ 0 \\ \begin{matrix} 0 \\ 0.28 \\ \begin{matrix} 0 \\ \begin{matrix} 0 \\ 0 \end{matrix} \end{matrix} \end{matrix} \end{matrix} \end{matrix} \end{matrix} \end{matrix} \end{matrix} \end{matrix} \end{matrix} \end{matrix} \end{matrix} \end{matrix} \end{matrix} \end{matrix} \end{matrix} \end{matrix} \end{matrix} \end{matrix} \end{matrix} \end{matrix} \end{matrix} \end{matrix} \end{matrix}\begin{matrix} 20 \\ 0 \\ \begin{matrix} 0 \\ 0 \\ \begin{matrix} 0 \\ 0 \\ \begin{matrix} 0 \\ 0 \\ \begin{matrix} 0 \\ 0 \\ \begin{matrix} 0 \\ 0 \\ \begin{matrix} 0 \\ 0 \\ \begin{matrix} 0 \\ 0 \\ \begin{matrix} 0.28 \\ 0 \\ \begin{matrix} 0 \\ 0 \\ \begin{matrix} 0 \\ 0 \\ \begin{matrix} 0 \\ 0 \\ \begin{matrix} 0 \\ 0 \\ \begin{matrix} 0 \\ 0 \\ \begin{matrix} 0 \\ 0 \\ \begin{matrix} 0 \\ 0 \\ \begin{matrix} 0 \\ 0 \\ \begin{matrix} 0 \\ 0.28 \\ \begin{matrix} 0 \\ 0.28 \\ \begin{matrix} 0 \\ 0 \\ \begin{matrix} 0 \\ 0 \\ \begin{matrix} 0 \\ 0.28 \\ \begin{matrix} 0 \\ \begin{matrix} 0 \\ 0 \end{matrix} \end{matrix} \end{matrix} \end{matrix} \end{matrix} \end{matrix} \end{matrix} \end{matrix} \end{matrix} \end{matrix} \end{matrix} \end{matrix} \end{matrix} \end{matrix} \end{matrix} \end{matrix} \end{matrix} \end{matrix} \end{matrix} \end{matrix} \end{matrix} \end{matrix} \end{matrix} \end{matrix}\begin{matrix} 21 \\ 0 \\ \begin{matrix} 0 \\ 0 \\ \begin{matrix} 0 \\ 0 \\ \begin{matrix} 0 \\ 0 \\ \begin{matrix} 0 \\ 0 \\ \begin{matrix} 0 \\ 0 \\ \begin{matrix} 0 \\ 0 \\ \begin{matrix} 0 \\ 0 \\ \begin{matrix} 0 \\ 0 \\ \begin{matrix} 0 \\ 0 \\ \begin{matrix} 0 \\ 0 \\ \begin{matrix} 0.35 \\ 0.23 \\ \begin{matrix} 0.23 \\ 0.23 \\ \begin{matrix} 0 \\ 0 \\ \begin{matrix} 0 \\ 0 \\ \begin{matrix} 0 \\ 0 \\ \begin{matrix} 0 \\ 0 \\ \begin{matrix} 0 \\ 0 \\ \begin{matrix} 0 \\ 0 \\ \begin{matrix} 0 \\ 0.35 \\ \begin{matrix} 0 \\ 0.35 \\ \begin{matrix} 0 \\ 0.35 \\ \begin{matrix} 0 \\ \begin{matrix} 0.23 \\ 0 \end{matrix} \end{matrix} \end{matrix} \end{matrix} \end{matrix} \end{matrix} \end{matrix} \end{matrix} \end{matrix} \end{matrix} \end{matrix} \end{matrix} \end{matrix} \end{matrix} \end{matrix} \end{matrix} \end{matrix} \end{matrix} \end{matrix} \end{matrix} \end{matrix} \end{matrix} \end{matrix} \end{matrix}\begin{matrix} 22 \\ 0 \\ \begin{matrix} 0 \\ 0 \\ \begin{matrix} 0 \\ 0 \\ \begin{matrix} 0 \\ 0 \\ \begin{matrix} 0 \\ 0 \\ \begin{matrix} 0 \\ 0 \\ \begin{matrix} 0 \\ 0 \\ \begin{matrix} 0 \\ 0 \\ \begin{matrix} 0 \\ 0 \\ \begin{matrix} 0 \\ 0 \\ \begin{matrix} 0 \\ 0.2 \\ \begin{matrix} 0 \\ 0 \\ \begin{matrix} 0 \\ 0 \\ \begin{matrix} 0 \\ 0 \\ \begin{matrix} 0 \\ 0 \\ \begin{matrix} 0 \\ 0 \\ \begin{matrix} 0 \\ 0 \\ \begin{matrix} 0 \\ 0 \\ \begin{matrix} 0 \\ 0 \\ \begin{matrix} 0 \\ 0 \\ \begin{matrix} 0 \\ 0 \\ \begin{matrix} 0 \\ 0 \\ \begin{matrix} 0 \\ \begin{matrix} 0 \\ 0 \end{matrix} \end{matrix} \end{matrix} \end{matrix} \end{matrix} \end{matrix} \end{matrix} \end{matrix} \end{matrix} \end{matrix} \end{matrix} \end{matrix} \end{matrix} \end{matrix} \end{matrix} \end{matrix} \end{matrix} \end{matrix} \end{matrix} \end{matrix} \end{matrix} \end{matrix} \end{matrix} \end{matrix}\begin{matrix} 23 \\ 0 \\ \begin{matrix} 0 \\ 0 \\ \begin{matrix} 0 \\ 0 \\ \begin{matrix} 0 \\ 0 \\ \begin{matrix} 0 \\ 0 \\ \begin{matrix} 0 \\ 0 \\ \begin{matrix} 0 \\ 0 \\ \begin{matrix} 0 \\ 0 \\ \begin{matrix} 0 \\ 0 \\ \begin{matrix} 0 \\ 0 \\ \begin{matrix} 0 \\ 0.28 \\ \begin{matrix} 0 \\ 0 \\ \begin{matrix} 0 \\ 0 \\ \begin{matrix} 0 \\ 0 \\ \begin{matrix} 0 \\ 0 \\ \begin{matrix} 0 \\ 0 \\ \begin{matrix} 0 \\ 0 \\ \begin{matrix} 0 \\ 0 \\ \begin{matrix} 0 \\ 0 \\ \begin{matrix} 0 \\ 0.28 \\ \begin{matrix} 0 \\ 0.28 \\ \begin{matrix} 0 \\ 0.28 \\ \begin{matrix} 0 \\ \begin{matrix} 0 \\ 0 \end{matrix} \end{matrix} \end{matrix} \end{matrix} \end{matrix} \end{matrix} \end{matrix} \end{matrix} \end{matrix} \end{matrix} \end{matrix} \end{matrix} \end{matrix} \end{matrix} \end{matrix} \end{matrix} \end{matrix} \end{matrix} \end{matrix} \end{matrix} \end{matrix} \end{matrix} \end{matrix} \end{matrix}\begin{matrix} 24 \\ 0 \\ \begin{matrix} 0 \\ 0 \\ \begin{matrix} 0 \\ 0 \\ \begin{matrix} 0 \\ 0 \\ \begin{matrix} 0 \\ 0 \\ \begin{matrix} 0 \\ 0 \\ \begin{matrix} 0 \\ 0 \\ \begin{matrix} 0 \\ 0 \\ \begin{matrix} 0 \\ 0 \\ \begin{matrix} 0 \\ 0 \\ \begin{matrix} 0 \\ 0.28 \\ \begin{matrix} 0 \\ 0 \\ \begin{matrix} 0 \\ 0 \\ \begin{matrix} 0 \\ 0 \\ \begin{matrix} 0 \\ 0 \\ \begin{matrix} 0 \\ 0 \\ \begin{matrix} 0 \\ 0 \\ \begin{matrix} 0 \\ 0 \\ \begin{matrix} 0 \\ 0 \\ \begin{matrix} 0 \\ 0.28 \\ \begin{matrix} 0 \\ 0.28 \\ \begin{matrix} 0 \\ 0.28 \\ \begin{matrix} 0 \\ \begin{matrix} 0 \\ 0 \end{matrix} \end{matrix} \end{matrix} \end{matrix} \end{matrix} \end{matrix} \end{matrix} \end{matrix} \end{matrix} \end{matrix} \end{matrix} \end{matrix} \end{matrix} \end{matrix} \end{matrix} \end{matrix} \end{matrix} \end{matrix} \end{matrix} \end{matrix} \end{matrix} \end{matrix} \end{matrix} \end{matrix}\begin{matrix} 25 \\ 0 \\ \begin{matrix} 0 \\ 0 \\ \begin{matrix} 0 \\ 0 \\ \begin{matrix} 0 \\ 0 \\ \begin{matrix} 0 \\ 0 \\ \begin{matrix} 0 \\ 0 \\ \begin{matrix} 0 \\ 0 \\ \begin{matrix} 0 \\ 0 \\ \begin{matrix} 0 \\ 0 \\ \begin{matrix} 0 \\ 0 \\ \begin{matrix} 0 \\ 0.28 \\ \begin{matrix} 0 \\ 0 \\ \begin{matrix} 0 \\ 0 \\ \begin{matrix} 0 \\ 0 \\ \begin{matrix} 0 \\ 0 \\ \begin{matrix} 0 \\ 0 \\ \begin{matrix} 0 \\ 0 \\ \begin{matrix} 0 \\ 0 \\ \begin{matrix} 0 \\ 0 \\ \begin{matrix} 0 \\ 0.28 \\ \begin{matrix} 0 \\ 0.28 \\ \begin{matrix} 0 \\ 0.28 \\ \begin{matrix} 0 \\ \begin{matrix} 0 \\ 0 \end{matrix} \end{matrix} \end{matrix} \end{matrix} \end{matrix} \end{matrix} \end{matrix} \end{matrix} \end{matrix} \end{matrix} \end{matrix} \end{matrix} \end{matrix} \end{matrix} \end{matrix} \end{matrix} \end{matrix} \end{matrix} \end{matrix} \end{matrix} \end{matrix} \end{matrix} \end{matrix} \end{matrix}\begin{matrix} 26 \\ 0 \\ \begin{matrix} 0 \\ 0 \\ \begin{matrix} 0 \\ 0 \\ \begin{matrix} 0 \\ 0 \\ \begin{matrix} 0 \\ 0 \\ \begin{matrix} 0 \\ 0 \\ \begin{matrix} 0 \\ 0 \\ \begin{matrix} 0 \\ 0 \\ \begin{matrix} 0 \\ 0 \\ \begin{matrix} 0 \\ 0 \\ \begin{matrix} 0 \\ 0 \\ \begin{matrix} 0 \\ 0 \\ \begin{matrix} 0 \\ 0 \\ \begin{matrix} 0 \\ 0.35 \\ \begin{matrix} 0.23 \\ 0.23 \\ \begin{matrix} 0.23 \\ 0 \\ \begin{matrix} 0 \\ 0 \\ \begin{matrix} 0 \\ 0 \\ \begin{matrix} 0 \\ 0 \\ \begin{matrix} 0 \\ 0.35 \\ \begin{matrix} 0 \\ 0.35 \\ \begin{matrix} 0 \\ 035 \\ \begin{matrix} 0 \\ \begin{matrix} 0.23 \\ 0 \end{matrix} \end{matrix} \end{matrix} \end{matrix} \end{matrix} \end{matrix} \end{matrix} \end{matrix} \end{matrix} \end{matrix} \end{matrix} \end{matrix} \end{matrix} \end{matrix} \end{matrix} \end{matrix} \end{matrix} \end{matrix} \end{matrix} \end{matrix} \end{matrix} \end{matrix} \end{matrix} \end{matrix}\begin{matrix} 27 \\ 0 \\ \begin{matrix} 0 \\ 0 \\ \begin{matrix} 0 \\ 0 \\ \begin{matrix} 0 \\ 0 \\ \begin{matrix} 0 \\ 0 \\ \begin{matrix} 0 \\ 0 \\ \begin{matrix} 0 \\ 0 \\ \begin{matrix} 0 \\ 0 \\ \begin{matrix} 0 \\ 0 \\ \begin{matrix} 0 \\ 0 \\ \begin{matrix} 0 \\ 0 \\ \begin{matrix} 0 \\ 0 \\ \begin{matrix} 0 \\ 0 \\ \begin{matrix} 0.2 \\ 0 \\ \begin{matrix} 0 \\ 0 \\ \begin{matrix} 0 \\ 0 \\ \begin{matrix} 0 \\ 0 \\ \begin{matrix} 0 \\ 0 \\ \begin{matrix} 0 \\ 0 \\ \begin{matrix} 0 \\ 0 \\ \begin{matrix} 0 \\ 0 \\ \begin{matrix} 0 \\ 0 \\ \begin{matrix} 0 \\ \begin{matrix} 0 \\ 0 \end{matrix} \end{matrix} \end{matrix} \end{matrix} \end{matrix} \end{matrix} \end{matrix} \end{matrix} \end{matrix} \end{matrix} \end{matrix} \end{matrix} \end{matrix} \end{matrix} \end{matrix} \end{matrix} \end{matrix} \end{matrix} \end{matrix} \end{matrix} \end{matrix} \end{matrix} \end{matrix} \end{matrix}\begin{matrix} 28 \\ 0 \\ \begin{matrix} 0 \\ 0 \\ \begin{matrix} 0 \\ 0 \\ \begin{matrix} 0 \\ 0 \\ \begin{matrix} 0 \\ 0 \\ \begin{matrix} 0 \\ 0 \\ \begin{matrix} 0 \\ 0 \\ \begin{matrix} 0 \\ 0 \\ \begin{matrix} 0 \\ 0 \\ \begin{matrix} 0 \\ 0 \\ \begin{matrix} 0 \\ 0 \\ \begin{matrix} 0 \\ 0 \\ \begin{matrix} 0 \\ 0 \\ \begin{matrix} 0.28 \\ 0 \\ \begin{matrix} 0 \\ 0 \\ \begin{matrix} 0 \\ 0 \\ \begin{matrix} 0 \\ 0 \\ \begin{matrix} 0 \\ 0 \\ \begin{matrix} 0 \\ 0 \\ \begin{matrix} 0 \\ 0.28 \\ \begin{matrix} 0 \\ 0.28 \\ \begin{matrix} 0 \\ 0.28 \\ \begin{matrix} 0 \\ \begin{matrix} 0 \\ 0 \end{matrix} \end{matrix} \end{matrix} \end{matrix} \end{matrix} \end{matrix} \end{matrix} \end{matrix} \end{matrix} \end{matrix} \end{matrix} \end{matrix} \end{matrix} \end{matrix} \end{matrix} \end{matrix} \end{matrix} \end{matrix} \end{matrix} \end{matrix} \end{matrix} \end{matrix} \end{matrix} \end{matrix}\begin{matrix} 29 \\ 0 \\ \begin{matrix} 0 \\ 0 \\ \begin{matrix} 0 \\ 0 \\ \begin{matrix} 0 \\ 0 \\ \begin{matrix} 0 \\ 0 \\ \begin{matrix} 0 \\ 0 \\ \begin{matrix} 0 \\ 0 \\ \begin{matrix} 0 \\ 0 \\ \begin{matrix} 0 \\ 0 \\ \begin{matrix} 0 \\ 0 \\ \begin{matrix} 0 \\ 0 \\ \begin{matrix} 0 \\ 0 \\ \begin{matrix} 0 \\ 0 \\ \begin{matrix} 0.28 \\ 0 \\ \begin{matrix} 0 \\ 0 \\ \begin{matrix} 0 \\ 0 \\ \begin{matrix} 0 \\ 0 \\ \begin{matrix} 0 \\ 0 \\ \begin{matrix} 0 \\ 0 \\ \begin{matrix} 0 \\ 0.28 \\ \begin{matrix} 0 \\ 0.28 \\ \begin{matrix} 0 \\ 0.28 \\ \begin{matrix} 0 \\ \begin{matrix} 0 \\ 0 \end{matrix} \end{matrix} \end{matrix} \end{matrix} \end{matrix} \end{matrix} \end{matrix} \end{matrix} \end{matrix} \end{matrix} \end{matrix} \end{matrix} \end{matrix} \end{matrix} \end{matrix} \end{matrix} \end{matrix} \end{matrix} \end{matrix} \end{matrix} \end{matrix} \end{matrix} \end{matrix} \end{matrix}\begin{matrix} 30 \\ 0 \\ \begin{matrix} 0 \\ 0 \\ \begin{matrix} 0 \\ 0 \\ \begin{matrix} 0 \\ 0 \\ \begin{matrix} 0 \\ 0 \\ \begin{matrix} 0 \\ 0 \\ \begin{matrix} 0 \\ 0 \\ \begin{matrix} 0 \\ 0 \\ \begin{matrix} 0 \\ 0 \\ \begin{matrix} 0 \\ 0 \\ \begin{matrix} 0 \\ 0 \\ \begin{matrix} 0 \\ 0 \\ \begin{matrix} 0 \\ 0 \\ \begin{matrix} 0.28 \\ 0 \\ \begin{matrix} 0 \\ 0 \\ \begin{matrix} 0 \\ 0 \\ \begin{matrix} 0 \\ 0 \\ \begin{matrix} 0 \\ 0 \\ \begin{matrix} 0 \\ 0 \\ \begin{matrix} 0 \\ 0.28 \\ \begin{matrix} 0 \\ 0.28 \\ \begin{matrix} 0 \\ 0.28 \\ \begin{matrix} 0 \\ \begin{matrix} 0 \\ 0 \end{matrix} \end{matrix} \end{matrix} \end{matrix} \end{matrix} \end{matrix} \end{matrix} \end{matrix} \end{matrix} \end{matrix} \end{matrix} \end{matrix} \end{matrix} \end{matrix} \end{matrix} \end{matrix} \end{matrix} \end{matrix} \end{matrix} \end{matrix} \end{matrix} \end{matrix} \end{matrix} \end{matrix}\begin{matrix} 31 \\ \begin{matrix} 0.35 \\ 0 \\ \begin{matrix} 0.23 \\ 0.23 \\ \begin{matrix} 0.23 \\ 0.35 \\ \begin{matrix} 0 \\ 0.23 \\ \begin{matrix} 0.23 \\ 0.23 \\ \begin{matrix} 0 \\ 0 \\ \begin{matrix} 0 \\ 0 \\ \begin{matrix} 0 \\ 0 \\ \begin{matrix} 0 \\ 0 \\ \begin{matrix} 0 \\ 0 \\ \begin{matrix} 0 \\ 0 \\ \begin{matrix} 0 \\ 0 \\ \begin{matrix} 0 \\ 0 \\ \begin{matrix} 0 \\ 0 \\ \begin{matrix} 0 \\ 0 \\ \begin{matrix} 0.35 \\ 0 \\ \begin{matrix} 0 \\ 0 \\ \begin{matrix} 0 \\ 0 \\ \begin{matrix} 0 \\ 0 \\ \begin{matrix} 0 \\ 0 \\ \begin{matrix} 0 \\ 0 \\ \begin{matrix} 0 \\ 0 \\ \begin{matrix} 0.23 \\ 0.23 \end{matrix} \end{matrix} \end{matrix} \end{matrix} \end{matrix} \end{matrix} \end{matrix} \end{matrix} \end{matrix} \end{matrix} \end{matrix} \end{matrix} \end{matrix} \end{matrix} \end{matrix} \end{matrix} \end{matrix} \end{matrix} \end{matrix} \end{matrix} \end{matrix} \end{matrix} \end{matrix} \end{matrix}\begin{matrix} 32 \\ 0 \\ \begin{matrix} 0 \\ 0 \\ \begin{matrix} 0 \\ 0 \\ \begin{matrix} 0 \\ 0 \\ \begin{matrix} 0 \\ 0 \\ \begin{matrix} 0 \\ 0 \\ \begin{matrix} 0 \\ 0 \\ \begin{matrix} 0 \\ 0 \\ \begin{matrix} 0 \\ 0 \\ \begin{matrix} 0 \\ 0 \\ \begin{matrix} 0 \\ 0 \\ \begin{matrix} 0 \\ 0 \\ \begin{matrix} 0 \\ 0 \\ \begin{matrix} 0 \\ 0 \\ \begin{matrix} 0 \\ 0 \\ \begin{matrix} 0.2 \\ 0 \\ \begin{matrix} 0 \\ 0 \\ \begin{matrix} 0 \\ 0 \\ \begin{matrix} 0 \\ 0 \\ \begin{matrix} 0 \\ 0 \\ \begin{matrix} 0 \\ 0 \\ \begin{matrix} 0 \\ 0 \\ \begin{matrix} 0 \\ \begin{matrix} 0 \\ 0 \end{matrix} \end{matrix} \end{matrix} \end{matrix} \end{matrix} \end{matrix} \end{matrix} \end{matrix} \end{matrix} \end{matrix} \end{matrix} \end{matrix} \end{matrix} \end{matrix} \end{matrix} \end{matrix} \end{matrix} \end{matrix} \end{matrix} \end{matrix} \end{matrix} \end{matrix} \end{matrix} \end{matrix}\begin{matrix} 33 \\ \begin{matrix} 0.35 \\ 0 \\ \begin{matrix} 0.23 \\ 0.23 \\ \begin{matrix} 0.23 \\ 0.35 \\ \begin{matrix} 0 \\ 0.23 \\ \begin{matrix} 0.23 \\ 0.23 \\ \begin{matrix} 0 \\ 0 \\ \begin{matrix} 0 \\ 0 \\ \begin{matrix} 0 \\ 0 \\ \begin{matrix} 0 \\ 0 \\ \begin{matrix} 0 \\ 0 \\ \begin{matrix} 0 \\ 0 \\ \begin{matrix} 0 \\ 0 \\ \begin{matrix} 0 \\ 0 \\ \begin{matrix} 0 \\ 0 \\ \begin{matrix} 0 \\ 0 \\ \begin{matrix} 0 \\ 0 \\ \begin{matrix} 0.35 \\ 0 \\ \begin{matrix} 0 \\ 0 \\ \begin{matrix} 0 \\ 0 \\ \begin{matrix} 0 \\ 0 \\ \begin{matrix} 0 \\ 0 \\ \begin{matrix} 0 \\ 0 \\ \begin{matrix} 0.23 \\ 0.23 \end{matrix} \end{matrix} \end{matrix} \end{matrix} \end{matrix} \end{matrix} \end{matrix} \end{matrix} \end{matrix} \end{matrix} \end{matrix} \end{matrix} \end{matrix} \end{matrix} \end{matrix} \end{matrix} \end{matrix} \end{matrix} \end{matrix} \end{matrix} \end{matrix} \end{matrix} \end{matrix} \end{matrix}\begin{matrix} 34 \\ 0 \\ \begin{matrix} 0 \\ 0 \\ \begin{matrix} 0 \\ 0 \\ \begin{matrix} 0 \\ 0 \\ \begin{matrix} 0 \\ 0 \\ \begin{matrix} 0 \\ 0 \\ \begin{matrix} 0 \\ 0 \\ \begin{matrix} 0 \\ 0 \\ \begin{matrix} 0 \\ 0 \\ \begin{matrix} 0 \\ 0 \\ \begin{matrix} 0 \\ 0 \\ \begin{matrix} 0 \\ 0 \\ \begin{matrix} 0 \\ 0 \\ \begin{matrix} 0 \\ 0 \\ \begin{matrix} 0 \\ 0 \\ \begin{matrix} 0 \\ 0 \\ \begin{matrix} 0.2 \\ 0 \\ \begin{matrix} 0 \\ 0 \\ \begin{matrix} 0 \\ 0 \\ \begin{matrix} 0 \\ 0 \\ \begin{matrix} 0 \\ 0 \\ \begin{matrix} 0 \\ 0 \\ \begin{matrix} 0 \\ \begin{matrix} 0 \\ 0 \end{matrix} \end{matrix} \end{matrix} \end{matrix} \end{matrix} \end{matrix} \end{matrix} \end{matrix} \end{matrix} \end{matrix} \end{matrix} \end{matrix} \end{matrix} \end{matrix} \end{matrix} \end{matrix} \end{matrix} \end{matrix} \end{matrix} \end{matrix} \end{matrix} \end{matrix} \end{matrix} \end{matrix}\begin{matrix} 35 \\ 0 \\ \begin{matrix} 0 \\ 0 \\ \begin{matrix} 0 \\ 0 \\ \begin{matrix} 0 \\ 0 \\ \begin{matrix} 0 \\ 0 \\ \begin{matrix} 0 \\ 0.35 \\ \begin{matrix} 0 \\ 0.23 \\ \begin{matrix} 0.23 \\ 0.23 \\ \begin{matrix} 0.35 \\ 0 \\ \begin{matrix} 0.23 \\ 0.23 \\ \begin{matrix} 0.23 \\ 0 \\ \begin{matrix} 0 \\ 0 \\ \begin{matrix} 0 \\ 0 \\ \begin{matrix} 0 \\ 0 \\ \begin{matrix} 0 \\ 0 \\ \begin{matrix} 0 \\ 0 \\ \begin{matrix} 0 \\ 0 \\ \begin{matrix} 0 \\ 0.35 \\ \begin{matrix} 0 \\ 0 \\ \begin{matrix} 0 \\ 0 \\ \begin{matrix} 0 \\ 0 \\ \begin{matrix} 0 \\ 0 \\ \begin{matrix} 0 \\ \begin{matrix} 0.23 \\ 0.23 \end{matrix} \end{matrix} \end{matrix} \end{matrix} \end{matrix} \end{matrix} \end{matrix} \end{matrix} \end{matrix} \end{matrix} \end{matrix} \end{matrix} \end{matrix} \end{matrix} \end{matrix} \end{matrix} \end{matrix} \end{matrix} \end{matrix} \end{matrix} \end{matrix} \end{matrix} \end{matrix} \end{matrix}\begin{matrix} 36 \\ 0 \\ \begin{matrix} 0 \\ 0 \\ \begin{matrix} 0 \\ 0 \\ \begin{matrix} 0 \\ 0 \\ \begin{matrix} 0 \\ 0 \\ \begin{matrix} 0 \\ 0 \\ \begin{matrix} 0 \\ 0 \\ \begin{matrix} 0 \\ 0 \\ \begin{matrix} 0 \\ 0 \\ \begin{matrix} 0 \\ 0 \\ \begin{matrix} 0 \\ 0 \\ \begin{matrix} 0 \\ 0 \\ \begin{matrix} 0 \\ 0 \\ \begin{matrix} 0 \\ 0 \\ \begin{matrix} 0 \\ 0 \\ \begin{matrix} 0 \\ 0 \\ \begin{matrix} 0 \\ 0 \\ \begin{matrix} 0.2 \\ 0 \\ \begin{matrix} 0 \\ 0 \\ \begin{matrix} 0 \\ 0 \\ \begin{matrix} 0 \\ 0 \\ \begin{matrix} 0 \\ 0 \\ \begin{matrix} 0 \\ \begin{matrix} 0 \\ 0 \end{matrix} \end{matrix} \end{matrix} \end{matrix} \end{matrix} \end{matrix} \end{matrix} \end{matrix} \end{matrix} \end{matrix} \end{matrix} \end{matrix} \end{matrix} \end{matrix} \end{matrix} \end{matrix} \end{matrix} \end{matrix} \end{matrix} \end{matrix} \end{matrix} \end{matrix} \end{matrix} \end{matrix}\begin{matrix} 37 \\ 0 \\ \begin{matrix} 0 \\ 0 \\ \begin{matrix} 0 \\ 0 \\ \begin{matrix} 0 \\ 0 \\ \begin{matrix} 0 \\ 0 \\ \begin{matrix} 0 \\ 0.35 \\ \begin{matrix} 0 \\ 0.23 \\ \begin{matrix} 0.23 \\ 0.23 \\ \begin{matrix} 0.35 \\ 0 \\ \begin{matrix} 0.23 \\ 0.23 \\ \begin{matrix} 0.23 \\ 0 \\ \begin{matrix} 0 \\ 0 \\ \begin{matrix} 0 \\ 0 \\ \begin{matrix} 0 \\ 0 \\ \begin{matrix} 0 \\ 0 \\ \begin{matrix} 0 \\ 0 \\ \begin{matrix} 0 \\ 0 \\ \begin{matrix} 0 \\ 0 \\ \begin{matrix} 0 \\ 0.35 \\ \begin{matrix} 0 \\ 0 \\ \begin{matrix} 0 \\ 0 \\ \begin{matrix} 0 \\ 0 \\ \begin{matrix} 0 \\ \begin{matrix} 0.23 \\ 0.23 \end{matrix} \end{matrix} \end{matrix} \end{matrix} \end{matrix} \end{matrix} \end{matrix} \end{matrix} \end{matrix} \end{matrix} \end{matrix} \end{matrix} \end{matrix} \end{matrix} \end{matrix} \end{matrix} \end{matrix} \end{matrix} \end{matrix} \end{matrix} \end{matrix} \end{matrix} \end{matrix} \end{matrix}\begin{matrix} 38 \\ 0 \\ \begin{matrix} 0 \\ 0 \\ \begin{matrix} 0 \\ 0 \\ \begin{matrix} 0 \\ 0 \\ \begin{matrix} 0 \\ 0 \\ \begin{matrix} 0 \\ 0 \\ \begin{matrix} 0 \\ 0 \\ \begin{matrix} 0 \\ 0 \\ \begin{matrix} 0 \\ 0 \\ \begin{matrix} 0 \\ 0 \\ \begin{matrix} 0 \\ 0 \\ \begin{matrix} 0 \\ 0 \\ \begin{matrix} 0 \\ 0 \\ \begin{matrix} 0 \\ 0 \\ \begin{matrix} 0 \\ 0 \\ \begin{matrix} 0 \\ 0 \\ \begin{matrix} 0 \\ 0 \\ \begin{matrix} 0 \\ 0 \\ \begin{matrix} 0.2 \\ 0 \\ \begin{matrix} 0 \\ 0 \\ \begin{matrix} 0 \\ 0 \\ \begin{matrix} 0 \\ 0 \\ \begin{matrix} 0 \\ \begin{matrix} 0 \\ 0 \end{matrix} \end{matrix} \end{matrix} \end{matrix} \end{matrix} \end{matrix} \end{matrix} \end{matrix} \end{matrix} \end{matrix} \end{matrix} \end{matrix} \end{matrix} \end{matrix} \end{matrix} \end{matrix} \end{matrix} \end{matrix} \end{matrix} \end{matrix} \end{matrix} \end{matrix} \end{matrix} \end{matrix}\begin{matrix} 39 \\ 0 \\ \begin{matrix} 0 \\ 0 \\ \begin{matrix} 0 \\ 0 \\ \begin{matrix} 0 \\ 0 \\ \begin{matrix} 0 \\ 0 \\ \begin{matrix} 0 \\ 0 \\ \begin{matrix} 0 \\ 0 \\ \begin{matrix} 0 \\ 0 \\ \begin{matrix} 0 \\ 0 \\ \begin{matrix} 0 \\ 0 \\ \begin{matrix} 0 \\ 0.35 \\ \begin{matrix} 0 \\ 0.23 \\ \begin{matrix} 0.23 \\ 0.23 \\ \begin{matrix} 0.35 \\ 0 \\ \begin{matrix} 0.23 \\ 0.23 \\ \begin{matrix} 0.23 \\ 0 \\ \begin{matrix} 0 \\ 0 \\ \begin{matrix} 0 \\ 0 \\ \begin{matrix} 0 \\ 0 \\ \begin{matrix} 0 \\ 0 \\ \begin{matrix} 0.35 \\ 0 \\ \begin{matrix} 0 \\ 0 \\ \begin{matrix} 0 \\ \begin{matrix} 0.23 \\ 0.23 \end{matrix} \end{matrix} \end{matrix} \end{matrix} \end{matrix} \end{matrix} \end{matrix} \end{matrix} \end{matrix} \end{matrix} \end{matrix} \end{matrix} \end{matrix} \end{matrix} \end{matrix} \end{matrix} \end{matrix} \end{matrix} \end{matrix} \end{matrix} \end{matrix} \end{matrix} \end{matrix} \end{matrix}\begin{matrix} 40 \\ 0 \\ \begin{matrix} 0 \\ 0 \\ \begin{matrix} 0 \\ 0 \\ \begin{matrix} 0 \\ 0 \\ \begin{matrix} 0 \\ 0 \\ \begin{matrix} 0 \\ 0 \\ \begin{matrix} 0 \\ 0 \\ \begin{matrix} 0 \\ 0 \\ \begin{matrix} 0 \\ 0 \\ \begin{matrix} 0 \\ 0 \\ \begin{matrix} 0 \\ 0 \\ \begin{matrix} 0 \\ 0 \\ \begin{matrix} 0 \\ 0 \\ \begin{matrix} 0 \\ 0 \\ \begin{matrix} 0 \\ 0 \\ \begin{matrix} 0 \\ 0 \\ \begin{matrix} 0 \\ 0 \\ \begin{matrix} 0 \\ 0 \\ \begin{matrix} 0 \\ 0 \\ \begin{matrix} 0 \\ 0.2 \\ \begin{matrix} 0 \\ 0 \\ \begin{matrix} 0 \\ 0 \\ \begin{matrix} 0 \\ \begin{matrix} 0 \\ 0 \end{matrix} \end{matrix} \end{matrix} \end{matrix} \end{matrix} \end{matrix} \end{matrix} \end{matrix} \end{matrix} \end{matrix} \end{matrix} \end{matrix} \end{matrix} \end{matrix} \end{matrix} \end{matrix} \end{matrix} \end{matrix} \end{matrix} \end{matrix} \end{matrix} \end{matrix} \end{matrix} \end{matrix}\begin{matrix} 41 \\ 0 \\ \begin{matrix} 0 \\ 0 \\ \begin{matrix} 0 \\ 0 \\ \begin{matrix} 0 \\ 0 \\ \begin{matrix} 0 \\ 0 \\ \begin{matrix} 0 \\ 0 \\ \begin{matrix} 0 \\ 0 \\ \begin{matrix} 0 \\ 0 \\ \begin{matrix} 0 \\ 0 \\ \begin{matrix} 0 \\ 0 \\ \begin{matrix} 0 \\ 0.35 \\ \begin{matrix} 0 \\ 0.23 \\ \begin{matrix} 0.23 \\ 0.23 \\ \begin{matrix} 0.35 \\ 0 \\ \begin{matrix} 0.23 \\ 0.23 \\ \begin{matrix} 0.23 \\ 0 \\ \begin{matrix} 0 \\ 0 \\ \begin{matrix} 0 \\ 0 \\ \begin{matrix} 0 \\ 0 \\ \begin{matrix} 0 \\ 0 \\ \begin{matrix} 0 \\ 0 \\ \begin{matrix} 0.35 \\ 0 \\ \begin{matrix} 0 \\ \begin{matrix} 0.23 \\ 0.23 \end{matrix} \end{matrix} \end{matrix} \end{matrix} \end{matrix} \end{matrix} \end{matrix} \end{matrix} \end{matrix} \end{matrix} \end{matrix} \end{matrix} \end{matrix} \end{matrix} \end{matrix} \end{matrix} \end{matrix} \end{matrix} \end{matrix} \end{matrix} \end{matrix} \end{matrix} \end{matrix} \end{matrix}\begin{matrix} 42 \\ 0 \\ \begin{matrix} 0 \\ 0 \\ \begin{matrix} 0 \\ 0 \\ \begin{matrix} 0 \\ 0 \\ \begin{matrix} 0 \\ 0 \\ \begin{matrix} 0 \\ 0 \\ \begin{matrix} 0 \\ 0 \\ \begin{matrix} 0 \\ 0 \\ \begin{matrix} 0 \\ 0 \\ \begin{matrix} 0 \\ 0 \\ \begin{matrix} 0 \\ 0 \\ \begin{matrix} 0 \\ 0 \\ \begin{matrix} 0 \\ 0 \\ \begin{matrix} 0 \\ 0 \\ \begin{matrix} 0 \\ 0 \\ \begin{matrix} 0 \\ 0 \\ \begin{matrix} 0 \\ 0 \\ \begin{matrix} 0 \\ 0 \\ \begin{matrix} 0 \\ 0 \\ \begin{matrix} 0 \\ 0 \\ \begin{matrix} 0 \\ 0.2 \\ \begin{matrix} 0 \\ 0 \\ \begin{matrix} 0 \\ \begin{matrix} 0 \\ 0 \end{matrix} \end{matrix} \end{matrix} \end{matrix} \end{matrix} \end{matrix} \end{matrix} \end{matrix} \end{matrix} \end{matrix} \end{matrix} \end{matrix} \end{matrix} \end{matrix} \end{matrix} \end{matrix} \end{matrix} \end{matrix} \end{matrix} \end{matrix} \end{matrix} \end{matrix} \end{matrix} \end{matrix}\begin{matrix} 43 \\ \begin{matrix} 0.35 \\ 0 \\ \begin{matrix} 0.23 \\ 0.23 \\ \begin{matrix} 0.23 \\ 0.35 \\ \begin{matrix} 0 \\ 0.23 \\ \begin{matrix} 0.23 \\ 0.23 \\ \begin{matrix} 0.35 \\ 0 \\ \begin{matrix} 0.23 \\ 0.23 \\ \begin{matrix} 0.23 \\ 0.35 \\ \begin{matrix} 0 \\ 0.23 \\ \begin{matrix} 0.23 \\ 0.23 \\ \begin{matrix} 0.35 \\ 0 \\ \begin{matrix} 0.23 \\ 0.23 \\ \begin{matrix} 0.23 \\ 0.35 \\ \begin{matrix} 0 \\ 0.23 \\ \begin{matrix} 0.23 \\ 0.23 \\ \begin{matrix} 0 \\ 0 \\ \begin{matrix} 0 \\ 0 \\ \begin{matrix} 0 \\ 0 \\ \begin{matrix} 0 \\ 0 \\ \begin{matrix} 0 \\ 0 \\ \begin{matrix} 0 \\ 0 \\ \begin{matrix} 0 \\ 0.35 \\ \begin{matrix} 0.23 \\ 0.23 \end{matrix} \end{matrix} \end{matrix} \end{matrix} \end{matrix} \end{matrix} \end{matrix} \end{matrix} \end{matrix} \end{matrix} \end{matrix} \end{matrix} \end{matrix} \end{matrix} \end{matrix} \end{matrix} \end{matrix} \end{matrix} \end{matrix} \end{matrix} \end{matrix} \end{matrix} \end{matrix} \end{matrix}\begin{matrix} 44 \\ 0 \\ \begin{matrix} 0 \\ 0 \\ \begin{matrix} 0 \\ 0 \\ \begin{matrix} 0 \\ 0 \\ \begin{matrix} 0 \\ 0 \\ \begin{matrix} 0 \\ 0 \\ \begin{matrix} 0 \\ 0 \\ \begin{matrix} 0 \\ 0 \\ \begin{matrix} 0 \\ 0 \\ \begin{matrix} 0 \\ 0 \\ \begin{matrix} 0 \\ 0 \\ \begin{matrix} 0 \\ 0 \\ \begin{matrix} 0 \\ 0 \\ \begin{matrix} 0 \\ 0 \\ \begin{matrix} 0 \\ 0 \\ \begin{matrix} 0 \\ 0 \\ \begin{matrix} 0 \\ 0 \\ \begin{matrix} 0 \\ 0 \\ \begin{matrix} 0 \\ 0 \\ \begin{matrix} 0 \\ 0 \\ \begin{matrix} 0 \\ 0 \\ \begin{matrix} 0 \\ 0.2 \\ \begin{matrix} 0 \\ \begin{matrix} 0 \\ 0 \end{matrix} \end{matrix} \end{matrix} \end{matrix} \end{matrix} \end{matrix} \end{matrix} \end{matrix} \end{matrix} \end{matrix} \end{matrix} \end{matrix} \end{matrix} \end{matrix} \end{matrix} \end{matrix} \end{matrix} \end{matrix} \end{matrix} \end{matrix} \end{matrix} \end{matrix} \end{matrix} \end{matrix}\begin{matrix} 45 \\ 0.28 \\ \begin{matrix} 0 \\ 0 \\ \begin{matrix} 0 \\ 0 \\ \begin{matrix} 0.28 \\ 0 \\ \begin{matrix} 0 \\ 0 \\ \begin{matrix} 0 \\ 0.28 \\ \begin{matrix} 0 \\ 0 \\ \begin{matrix} 0 \\ 0 \\ \begin{matrix} 0.28 \\ 0 \\ \begin{matrix} 0 \\ 0 \\ \begin{matrix} 0 \\ 0.28 \\ \begin{matrix} 0 \\ 0 \\ \begin{matrix} 0 \\ 0 \\ \begin{matrix} 0.28 \\ 0 \\ \begin{matrix} 0 \\ 0 \\ \begin{matrix} 0 \\ 0.28 \\ \begin{matrix} 0 \\ 0.28 \\ \begin{matrix} 0 \\ 0.28 \\ \begin{matrix} 0 \\ 0.28 \\ \begin{matrix} 0 \\ 0.28 \\ \begin{matrix} 0 \\ 0.28 \\ \begin{matrix} 0 \\ 0.28 \\ \begin{matrix} 0 \\ \begin{matrix} 0 \\ 0 \end{matrix} \end{matrix} \end{matrix} \end{matrix} \end{matrix} \end{matrix} \end{matrix} \end{matrix} \end{matrix} \end{matrix} \end{matrix} \end{matrix} \end{matrix} \end{matrix} \end{matrix} \end{matrix} \end{matrix} \end{matrix} \end{matrix} \end{matrix} \end{matrix} \end{matrix} \end{matrix} \end{matrix}\begin{matrix} 46 \\ 0 \\ \begin{matrix} 0 \\ 0 \\ \begin{matrix} 0 \\ 0 \\ \begin{matrix} 0 \\ 0 \\ \begin{matrix} 0 \\ 0 \\ \begin{matrix} 0 \\ 0 \\ \begin{matrix} 0 \\ 0 \\ \begin{matrix} 0 \\ 0 \\ \begin{matrix} 0 \\ 0 \\ \begin{matrix} 0 \\ 0 \\ \begin{matrix} 0 \\ 0 \\ \begin{matrix} 0 \\ 0 \\ \begin{matrix} 0 \\ 0 \\ \begin{matrix} 0 \\ 0 \\ \begin{matrix} 0 \\ 0 \\ \begin{matrix} 0 \\ 0.28 \\ \begin{matrix} 0 \\ 0.28 \\ \begin{matrix} 0 \\ 0.28 \\ \begin{matrix} 0 \\ 0.28 \\ \begin{matrix} 0 \\ 0.28 \\ \begin{matrix} 0 \\ 0.28 \\ \begin{matrix} 0 \\ 0.28 \\ \begin{matrix} 0 \\ \begin{matrix} 0 \\ 0 \end{matrix} \end{matrix} \end{matrix} \end{matrix} \end{matrix} \end{matrix} \end{matrix} \end{matrix} \end{matrix} \end{matrix} \end{matrix} \end{matrix} \end{matrix} \end{matrix} \end{matrix} \end{matrix} \end{matrix} \end{matrix} \end{matrix} \end{matrix} \end{matrix} \end{matrix} \end{matrix} \end{matrix} \right]$$

**Part B. Estimation of** **the area of 28 surfaces in ICU.**

**Table B.1. Estimated area of 28 surfaces in ICU.**

| **Type** | **Surfaces** | **Area (cm^2^) (****Possible contact area)** |
| --- | --- | --- |
| Communal surfaces (CSs) | Sink surround | 50$\times$50=2500 (50$\times$10=500) |
|  | Curtain | 100$\times$200=20000 (100$\times$10=1000) |
|  | ABHR Dispense | 1.5$\times$1.5=2.25 (2.25) |
|  | Paper towel | 50$\times$30=1500 (30$\times$10=300) |
|  | Soap dispenser | 10$\times$6=60 (60) |
|  | Thermostat | 10$\times$15=150 (150) |
| Summary | | Total possible contact area: 2012.25 cm^2^ |
| Clinical equipment  (CE) | Supply cart | 50$\times$80=400 (400) |
|  | IV pump | 10$\times$0.2=2 (2) |
|  | Monitor | 10$\times$5=50 (50) |
|  | IV tubing | 10$\times$0.2=2 (2) |
|  | Monitor cables | 100$\times$0.03=3 (3) |
|  | Ventilator Circuit | 180$\times$3.14$\times$0.11^2^=6.8 (6.8) |
|  | Suction | 10$\times$3.14$\times$3^2^=282 (282) |
|  | Ventilator | 3.14$\times$10$\times$(10^2^+6^2^)^0.5^ =366 (366) |
|  | Keyboard | 50$\times$10=500 (500) |
|  | Mouse | 10$\times$4=40 (40) |
|  | Tube feed pump | 3.14$\times$1^2^$\times$15=47 (47) |
|  | Oxygen | 3.14$\times$10$\times$(10^2^+6^2^)^0.5^=366 (366) |
|  | SCD | 0 |
|  | Thermometer | 3$\times$15$\times$2=90 (90) |
|  | Linen hamper | 4$\times$50$\times$4=800 (800) |
| Summary | | Total possible contact area: 2954.8 cm^2^ |
| HTSs | Bed rail | 100$\times$0.8=80 (80) |
|  | Bed surface | 200$\times$80=16000 (80$\times$25=2000) |
| Summary | | Total possible contact area: 2080 cm^2^ |
| MTSs | Trash can | 20$\times$20=400 (20$\times$10=200) |
|  | Bedside table | 40$\times$40=1600 (1600) |
| Summary | | Total possible contact area: 1800 cm^2^ |
| LTSs | Over-bed table | 100$\times$20=2000 (2000) |
|  | Chair | 30$\times30=$900 (30$\times$20=600) |
|  | Light switch | 1.5$\times$1.5=2.25 (2.25) |
| Summary | | Total possible contact area: 2602.25 cm^2^ |

**Part C. Model parameters**

**Touching behaviors in ICU**

**a. Direct contact between HCWs and patients.**

McArdle et al. observed different HCWs contact rate with patients (McArdle et al. 2006) (see detail in Table C.1). Just as the assumption in the main text, each patient would visit by nurse 8 times per day, by doctor 1 times per day. The direct contact rates with nurses and ICU doctors per interaction per person are 110$\div$8=14 and 9 times per interaction. The study by Cheng et al. (2015) in a fully occupied six-bedded cubicle shown that the medical staff and nursing staff direct contact with patient’s body are 81 and 253 times during a 66-hour observation (two hours per day for 33 days). We assume that the observation time of each day is the working hour of HCWs, and during the two hours observation, the nurse and patient just visit one patient. Because the number of doctors and nurses in the ICU is unknown in the study, it’s assumed that the doctor/nurse to patient ratio are 1:6 (Temime et al. 2009) and 1:2 (Dara and Afessa 2005) respectively. So in the six-bedded cubicle, which is fully occupied, the estimated direct contact rates with per patient by doctor and nurse per day are 81$\div$33=2.5 and 253$\div$33=7.7 times per patient per interaction respectively. The estimated direct contact rate by doctor in the study by Cheng et al. (2015) is much lower than that from McArdle et al. (2006). It’s possible because that the patient in the study by Cheng et al. (2015) is not only cared by one doctor. It’s assumed that the contact with patient’s body is touching patient’s hands. In this study, we took the average value from the above two studies, the estimated doctors direct contact rate with patient’s hands is 6 times per day. Since in this study, it’s assumed that doctor only visited each patient once per day with duration 27 minutes, so the doctor direct contact rate with patient’s hands during healthcare visit is 6$\div$0.45=13 times per hour, with standard deviation (SD) 10 (the SD of the two searched values from literatures). One nurse are assumed care each patient 4 time per day, so the estimated nurse direct contact rate with each patient during healthcare visit is 11$\div$0.45=24 times per hour.

**b. HCWs’ contact with environmental surfaces.**

The observation study by McArdle et al. identified that the each HCW in ICU contacted environmental surface 191 times per day (95% confidence interval (CI) 174-210) (McArdle et al. 2006), but no detailed contact information on each surface. Huslage et al. quantitatively defined the HCWs contact rate on 28 surfaces in ICU (Huslage et al. 2010). And according to our surface classification, the hospital surfaces are divided into five types: communal surfaces (CSs), clinical equipment (CE), high-touch surfaces (HTSs), medium-touch surfaces (MTSs) and low-touch surfaces (LTSs). The estimated number of HCWs contact on CSs, CE, HTSs, MTSs and LTSs per interaction is 0.70, 1.44, 6.95, 1.55, 0.7 (see detail in Table C.2), but no detailed information the doctors and nurse contact rate on each surface respectively. Another study provided the doctors and nurse contact on hospital environmental surfaces respectively, but only the contact information rates on 9 top-touch surfaces are provided (see detail in Table C.3). According to the study on the 9 surfaces, the overall number of contact by nurse on surfaces is about 3.5 times of that by medical staff (Cheng et al. 2015). In addition, take the bed rail as an example, which is a high-touch surface in the study by Huslage et al. (2010). The medical staff and nurse staff would touch the bed rail 83 times and 377 times during a 66-hour observation (33 days, two hour for each day), according to the assumption in the main text, during the two-hour duration, the nurse and doctor would care each patient once at most, assume that the observation two-hour are just the healthcare visit time for doctor and nurse. So each medical staff would touch each bed rail 83$\div$33=2.5 times per interaction, and one nurse would touch each bedrail 377$\div$33=11.4 times per interaction. According to the estimation from the study by Cheng et al. (2015). The number of contact on bed rail by HCWs per interaction is 7 times ((11.4+2.5)$\div$2=7.0), which is close to the result from the study by Huslage et al. (2010). So assume that the nurse contact rate on hospital surfaces is 3.5 times of that by doctors, and the results from the study by Huslage et al. (2010) is used to estimate the HCW contact rate on hospital surfaces in this study. Then the estimated doctor’s contact rate on CSs during healthcare is (0.7$\times$2/4.5)/0.45=0.7 per hour. Similarly, the estimated doctor’s contact rates on CE, HTSs, MTSs and LTSs per hour during healthcare are 1.4, 6.9, 1.5 and 0.7 respectively. The estimated nurse’s contact rates on CSs, CE, HTSs, MTSs and LTSs per hour during healthcare are 4.6, 5.0, 24.0, 5.4 and 2.4 respectively.

**c. Patients’ contact with environmental surfaces.**

Cheng et al. measured the patients contact on 9 environmental surfaces in a fully occupied six-bedded cubicle, the overall number of patient contact on the 9 environmental surfaces is about 67% of that by doctor (Cheng et al. 2015). So it’s assumed that the patient’s contact rate on CSs is 0.7$\times$0.67=0.5 per hour, Similarly, the patient’s contact rates on HTSs, MTSs and LTSs are 4.6, 1.0 and 0.5 per hour respectively. It’s assumed that patients didn’t touch CE.

**Table C.1**. Estimated mean number of daily contacts/patient between different groups of healthcare workers, with 95% confidence interval (McArdle et al. 2006).

| Staff group | Direct contact | Indirect contact |
| --- | --- | --- |
| Cohorted nurses | 109.5 (98.6-120.9) | 54.5 (47.7–61.6) |
| Non-cohorted nurses | 32.3 (25.3-41.7) | 88.1 (78.0–100.1) |
| ICU doctors | 8.6 (6.0-13.2) | 30.4 (24.9–39.0) |
| Non-ICU doctors | 2.3 (1.0-5.8) | 4.8 (2.7–8.8) |
| Physiotherapists | 5.0 (3.0-7.2) | 5.0 (3.3–7.7) |
| Other staff | 1.9 (0.7-3.9) | 8.0 (5.8–11.1) |
| All staff | 159.4 (143.7-178.1) | 190.9 (173.5–210.0) |

**Table C.2**. The number of contact on 28 surfaces in ICU by HCWs during each interaction (Huslage et al. 2010).

| **Categories** | **Surfaces** | **Contact rate (per interaction)** |
| --- | --- | --- |
| Communal surfaces (CSs) | Sink surround | 0.9 |
|  | Curtain | 0.9 |
|  | ABHR Dispense | 0.8 |
|  | Paper towel | 0.8 |
|  | Soap dispenser | 0.7 |
|  | Thermostat | 0.1 |
| Summary | | Mean 0.7; SD 0.30 |
| Clinical equipment  (CE) | Supply cart | 4.0 |
|  | IV pump | 2.5 |
|  | Monitor | 2.2 |
|  | IV tubing | 2.1 |
|  | Monitor cables | 2.0 |
|  | Ventilator Circuit | 1.8 |
|  | Suction | 1.6 |
|  | Ventilator | 1.5 |
|  | Keyboard | 1.3 |
|  | Mouse | 1.3 |
|  | Tube feed pump | 0.4 |
|  | Oxygen | 0.3 |
|  | SCD | 0.2 |
|  | Thermometer | 0.2 |
|  | Linen hamper | 0.2 |
| Summary | | Mean 1.44; SD 1.08 |
| HTSs | Bed rail | 7.8 |
|  | Bed surface | 6.1 |
| Summary | | Mean 6.95; SD 1.20 |
| MTSs | Trash can | 1.9 |
|  | Bedside table | 1.2 |
| Summary | | Mean 1.55; SD 0.49 |
| LTSs | Over-bed table | 1.0 |
|  | Chair | 0.7 |
|  | Light switch | 0.4 |
| Summary | | Mean 0.7; SD 0.3 |

**Table C.3**. The number of contacts on the top 10 high-touch items by different categories of persons during a 66-hour observation (Cheng et al. 2015).

| Items | Medical staff | Nurse staff | Patients | Visitors |
| --- | --- | --- | --- | --- |
| Bedside rail | 83 | 377 | 111 | 29 |
| Bedside table | 141 | 361 | 77 | 18 |
| Patient body | 81 | 253 | 2 | 15 |
| Patient file | 110 | 335 | 15 | 2 |
| Linen | 56 | 135 | 31 | 23 |
| Curtain | 23 | 114 | 22 | 6 |
| Bed frame | 23 | 104 | 24 | 5 |
| Locker | 3 | 22 | 27 | 13 |
| Blood pressure cuff | 1 | 38 | 1 | 0 |
| Syringe | 11 | 69 | 1 | 0 |
| Others | 167 | 909 | 159 | 15 |

**d. MRSA inactivation on hospital surfaces**

MRSA colonized patient are known to emit MRSA into environment in association with epithelial cells (Hambraeus, 1973; Gehanno et al. 2009). We assumed that the MRSA colonized patient only emitted MRSA on his/her nearby area. A field study showed there was no significant difference in MRSA counts on the floor area within 3 meters of the MRSA patient’s head (Gehanno et al. 2009). So it’s further assumed that the emission rates on three kinds private surfaces near the MRSA colonized patient are the same. And during healthcare on index patient, the CE would also be near the index patient, so it’s assumed that, during the healthcare visit to index patient, there is also MRSA pathogen emitted to the CE, with the same emission rate on the index patient nearby private surfaces. Different patients would have different pathogen emission rate, the lognormal distribution is chosen to produce a probability distribution of the pathogen emission rate, with mean value 0.6 CFU/(cm^2^-h) (Plipat et al. 2013) and standard deviation (SD) 0.03.

The inactivation of MRSA has been experimentally evaluated on a number of substrates present in healthcare settings (Ayliffe et al. 1967; Desai et al. 2011; Huang et al. 2006; Oie and Kamiya 1996; Pérez-Rodríguez et al. 2013; Petti et al. 2012). All these experiments were performed under temperature and relative humidity conditions of normal indoor environments.

The mean inactivation rates varied an order of magnitude in different studies, but qualitatively, variability within each substrate type was similar to variability between substrate types (Table C.4). This suggests that there is no statistical basis to distinguish inactivation rates among substrate types. As a result, the inactivation rates were pooled across substrates and experiments. The inactivation rate of MRSA on substrates was modeled by positive values from a normal distribution with mean 0.0082/h and standard deviation 0.0068/h.

**Table C.4.** Inactivation rates of MRSA on substrates.

| **Substrate** | **Inactivation Rate (h^-1^)** | | **Reference** |
| --- | --- | --- | --- |
|  | **Mean** | **SD** |  |
| Vinyl | 0.0173 | 0.0140 | Ayliffe et al. 1967 |
| Vinyl | 0.0057 | 0.0004 | Huang et al. 2006 |
| Plastic | 0.0054 | 0.0060 | Desai et al. 2011 |
| Upholstery | 0.0029 | 0.0013 | Petti et al. 2012 |
| Textile | 0.0041 | <0.0001 | Huang et al. 2006 |
| Dry Mops | 0.0050 | 0.0011 | Oie and Kamiya 1996 |
| Stainless steel | 0.011 | 0.0060 | Pérez-Rodríguez et al. 2013 |
| Ceramic | 0.0034 | - | Huang et al. 2006 |
| Pooled | 0.0082 | 0.0068 |  |

To our knowledge, no studies were identified that measured the MRSA survival on human hands. Gontijo et al. (1985) studied the *S.* aureus survival on hands, and concluded that the loss was due to desiccation and after the initial five minutes, the decline was less pronounced. In this study, we used the data of this second phase (after the initial five minutes), assuming a first-order decay. The die-off rate on the fingertip was estimated as 0.57/h in the study mentioned above, and the same value was used in our model.

**e. Compliance rate of hand and surface hygiene in ICU**

Table C.5 listed the summary of the HCWs hand hygiene compliance rates in ICU.

Table C.5. Compliance rate of hand hygiene in ICU.

| Setting | Average compliance rate | Source |
| --- | --- | --- |
| ICU | 30% | Preston et al. 1981 |
| ICUs | 41% | Albert and Condie 1981 |
| ICUs | 28% | Albert and Condie 1981 |
| PICU | 30% | Donowitz 1987 |
| ICU | 32% | Graham 1990 |
| ICU | 81% | Dubbert et al. 1990 |
| SICU | 51% | Pettinger and Nettleman 1991 |
| NICU | 29% | Larson et al. 1992 |
| ICUs | 40% | Doebbeling et al. 1992 |
| ICUs | 40% | Zimakoff et al. 1992 |
| ICUs | 36% | Pittet et al. 1999 |
| ICUs | 48% | Eveillard et al. 2009 |
| Average | 42% |  |

The summary of the model parameters in the model is listed in Table C.6.

**Table C.6. Summary of model parameter value and source.**

| **Parameter** | | **Description**^a^ | **Value** | **Source** |
| --- | --- | --- | --- | --- |
| **Surface area parameters** | | | | |
| *A_h_* | | Area of the palm of one hand [cm^2^] | 203 | Lee et al. 2007 |
| *A_ht_* | | Possible contact area of HTSs [cm^2^] | 2300 | Estimated |
| *A_mt_* | | Possible contact area of MTSs [cm^2^] | 2300 | Estimated |
| *A_lt_* | | Possible contact area of LTSs [cm^2^] | 2300 | Estimated |
| *A_hs_* | | Contact area between environmental surface and hand during hand-to-surface contact [cm^2^] | 42 | AuYeung et al. 2008 |
| *A_f_* | | Area of fingertip contact with exposure skin [cm^2^] | 2 | Wiertlewski  and Hayward  2012 |
| *A_e_* | | Area of the exposure skin^b^ [cm^2^] | 4 | Assumed |
| **MRSA shedding parameters** | | | | |
| *e_ht_* | | Rate of MRSA emission to the HTSs of MRSA colonized patient [CFU/cm^2.^h] (lognormal distribution) | Mean 0.6 | Estimate |
|  | |  | SD^c^: 0.3 |  |
| *e_mt_* | | Rate of MRSA emission to the MTSs of MRSA colonized patient [CFU/cm^2.^h] (lognormal distribution) | Mean 0.6 | Plipat et al. 2013 |
|  | |  | SD: 0.3 |  |
| *e_lt_* | | Rate of MRSA emission to the LTSs of MRSA colonized patient [CFU/cm^2.^h] (lognormal distribution) | Mean 0.6 | Plipat et al. 2013 |
|  | |  | SD: 0.3 |  |
| *e_h_* | | Rate of MRSA emission to on the MRSA colonized patient’s hands [CFU/cm^2.^h] (lognormal distribution) | Mean 0.6 | Plipat et al. 2013 |
|  | |  | SD: 0.3 |  |
| *e_ce_* | | Rate of MRSA emission to the CE during healthcare of the index patient [CFU/cm^2.^h] (lognormal distribution) | Mean 0.6 | Plipat et al. 2013 |
|  | |  | SD: 0.3 |  |
| **MRSA inactivation parameters** | | | | |
| *d_h_* | | Rate of MRSA inactivation on hands of patients and HCWs [/h] | 0.57 | Gontijo et a. 1985 |
| *d_ht_* | | Rate of MRSA inactivation on HTSs [/h] (normal distribution) | Mean: 0.0082 | Estimated |
|  | |  | SD: 0.0068 |  |
| *d_mt_* | | Rate of MRSA inactivation on MTSs [/h] (normal distribution) | Mean: 0.0082 | Estimated |
|  | |  | SD: 0.0068 |  |
| *d_lt_* | | Rate of MRSA inactivation on LTSs [/h] (normal distribution) | Mean: 0.0082 | Estimated |
|  | |  | SD: 0.0068 |  |
| *d_cs_* | | Rate of MRSA inactivation on CSs [/h] (normal distribution) | Mean: 0.0082 | Estimated |
|  | |  | SD: 0.0068 |  |
| *d_ce_* | | Rate of MRSA inactivation on CE [/h] (normal distribution) | Mean: 0.0082 | Estimated |
|  | |  | SD: 0.0068 |  |
| **Contact event parameters** | | | | |
| $\beta_{e}$ | | Rate of hand contact rate with exposure skin [/h] | 5 | Assumed |
| $\beta_{pcs}$ | | Rate of patient hand contact rate with CSs [/h] (normal distribution) | Mean: 0.5 | Estimated |
|  | |  | SD: 0.2 |  |
| $\beta_{pht}$ | | Rate of patient hand contact rate with HTSs [/h] (normal distribution) | Mean: 4.6 | Estimated |
|  | |  | SD: 0.8 |  |
| $\beta_{pmt}$ | | Rate of patient hand contact rate with MTSs [/h] (normal distribution) | Mean: 1.0 | Estimated |
|  | |  | SD: 0.3 |  |
| $\beta_{plt}$ | | Rate of patient hand contact rate with LTSs [/h] (normal distribution) | Mean: 0.5 | Estimated |
|  | |  | SD: 0.2 |  |
| $\beta_{np}$ | | Rate of nurse hand contact with a patient [/h] (normal distribution) | Mean: 24.0 | Estimated |
|  | |  | SD: 9.0 |  |
| $\beta_{nht}$ | | Rate of nurse hand contact with HTSs during healthcare visit [/h] (normal distribution) | Mean: 24.1 | Estimated |
|  | |  | SD: 4.2 |  |
| $\beta_{nmt}$ | | Rate of nurse hand contact with MTSs during one patient healthcare visit [/h] (normal distribution) | Mean: 5.3 | Estimated |
|  | |  | SD: 1.7 |  |
| $\beta_{nlt}$ | | Rate of nurse hand contact with LTSs during healthcare visit [/h] (normal distribution) | Mean: 2.5 | Estimated |
|  | |  | SD: 1.0 |  |
| $\beta_{ncs}$ | | Rate of nurse hand contact with CSs during healthcare visit [/h] (normal distribution) | Mean: 2.5 | Estimated |
|  | |  | SD: 1.0 |  |
| $\beta_{nce}$ | | Rate of nurse hand contact with CE during healthcare visit [/h] (normal distribution) | Mean: 4.9 | Estimated |
|  | |  | SD: 3.7 |  |
| $\beta_{dp}$ | | Rate of doctor (physician) hand contact with a patient during one patient healthcare visit [/h] (normal distribution) | Mean: 13.0 | Estimated |
|  | |  | SD: 10.0 |  |
| $\beta_{dht}$ | | Rate of doctor (physician) hand contact with HTSs during one patient healthcare visit [/h] (normal distribution) | Mean: 6.9 | Estimated |
|  | |  | SD: 1.2 |  |
| $\beta_{dmt}$ | | Rate of doctor (physician) hand contact with MTSs during one patient healthcare visit [/h] (normal distribution) | Mean: 1.5 | Estimated |
|  | |  | SD: 0.5 |  |
| $\beta_{dlt}$ | | Rate of doctor (physician) hand contact with LTSs during one patient healthcare visit [/h] (normal distribution) | Mean: 0.7 | Estimated |
|  | |  | SD: 0.3 |  |
| $\beta_{dcs}$ | | Rate of doctor hand contact with CSs during healthcare visit [/h] (normal distribution) | Mean: 0.7 | Estimated |
|  | |  | SD: 0.3 |  |
| $\beta_{dce}$ | | Rate of doctor hand contact with CE during healthcare visit [/h] (normal distribution) | Mean: 1.4 | Estimated |
|  | |  | SD: 1.0 |  |
| **Transfer efficacy** **parameters** | | | | |
| *τ_hn_* | Efficiency of MRSA transfer from hand to exposure skin upon contact [-] | | 0.35 | Rusin et al. 2002 |
| *τ_nh_* | Efficiency of MRSA transfer from exposure skin to hand upon contact [-] | | 0.2 | Plipat et al. 2013 |
| *τ_hht_* | Efficiency of MRSA transfer from hand to HTSs upon contact [-] | | 0.23 | Lopez et al. 2013 |
| *τ_hth_* | Efficiency of MRA transfer from HTSs to hand upon contact [-] | | 0.28 | Lopez 2013 |
| *τ_hmt_* | Efficiency of MRSA transfer from hand to MTSs upon contact [-] | | 0.23 | Lopez 2013 |
| *τ_mth_* | Efficiency of MRA transfer from MTSs to hand upon contact [-] | | 0.28 | Lopez 2013 |
| *τ_hlt_* | Efficiency of MRSA transfer from hand to LTSs upon contact [-] | | 0.23 | Lopez 2013 |
| *τ_lth_* | Efficiency of MRA transfer from LTSs to hand upon contact [-] | | 0.28 | Lopez 2013 |
| *τ_hcs_* | Efficiency of MRSA transfer from hand to CSs upon contact [-] | | 0.23 | Lopez 2013 |
| *τ_csh_* | Efficiency of MRA transfer from CSs to hand upon contact [-] | | 0.28 | Lopez 2013 |
| *τ_hme_* | Efficiency of MRSA transfer from hand to CE upon contact [-] | | 0.23 | Lopez 2013 |
| *τ_meh_* | Efficiency of MRA transfer from CE to hand upon contact [-] | | 0.28 | Lopez 2013 |
| **Intervention parameters** | | | | |
| *I_h_* | Nurse and doctor (physician) hand cleaning efficiency [-] | | 0.90 | Temime et al. 2009 |
| *I_ht_* | HTSs cleaning efficacy | | 0.91 | Cheng et al. 2011 |
| *I_mt_* | MTSs cleaning efficacy | | 0.91 | Cheng et al. 2011 |
| *I_lt_* | LTSs cleaning efficacy | | 0.91 | Cheng et al. 2011 |
| *I_w_* | CSs cleaning efficacy | | 0.91 | Cheng et al. 2011 |

^a^HTSs: High-touch surfaces, MTSs: Medium-touch surfaces, LTSs: Low-touch surfaces, CSs: communal surfaces, CE: clinical equipment

^b^In our model, the individual 1 is assumed to be the MRSA colonized patient (index patient), so the “exposure skin” of patient 1 refer to the nose area, which has high MRSA concentration and act as the source of pathogen.

^c^SD: standard deviation

**Part D. Sensitivity analysis of key parameters**

Since the aim of this study is to quantify the relative importance of different surfaces with different contact frequencies on MRSA transmission in ICUs, we used the relative percentage increase of mean pathogen concentration on patients 2 and 3 if non-compliance of cleaning on one type of surfaces happened, as the index to do the sensitivity analysis. We have showed that the HTSs played the most important role in the main text, so the percentage increase of mean pathogen concentration if the HTSs were not cleaned is set as the reference. According to the sensitivity analysis, we have showed that the with the change of the parameter value, though the relative importance of different surfaces would change little, but all results showed that the high-touch surfaces was the most important in the surface cleaning on controlling MRSA transmission in the ICUs.


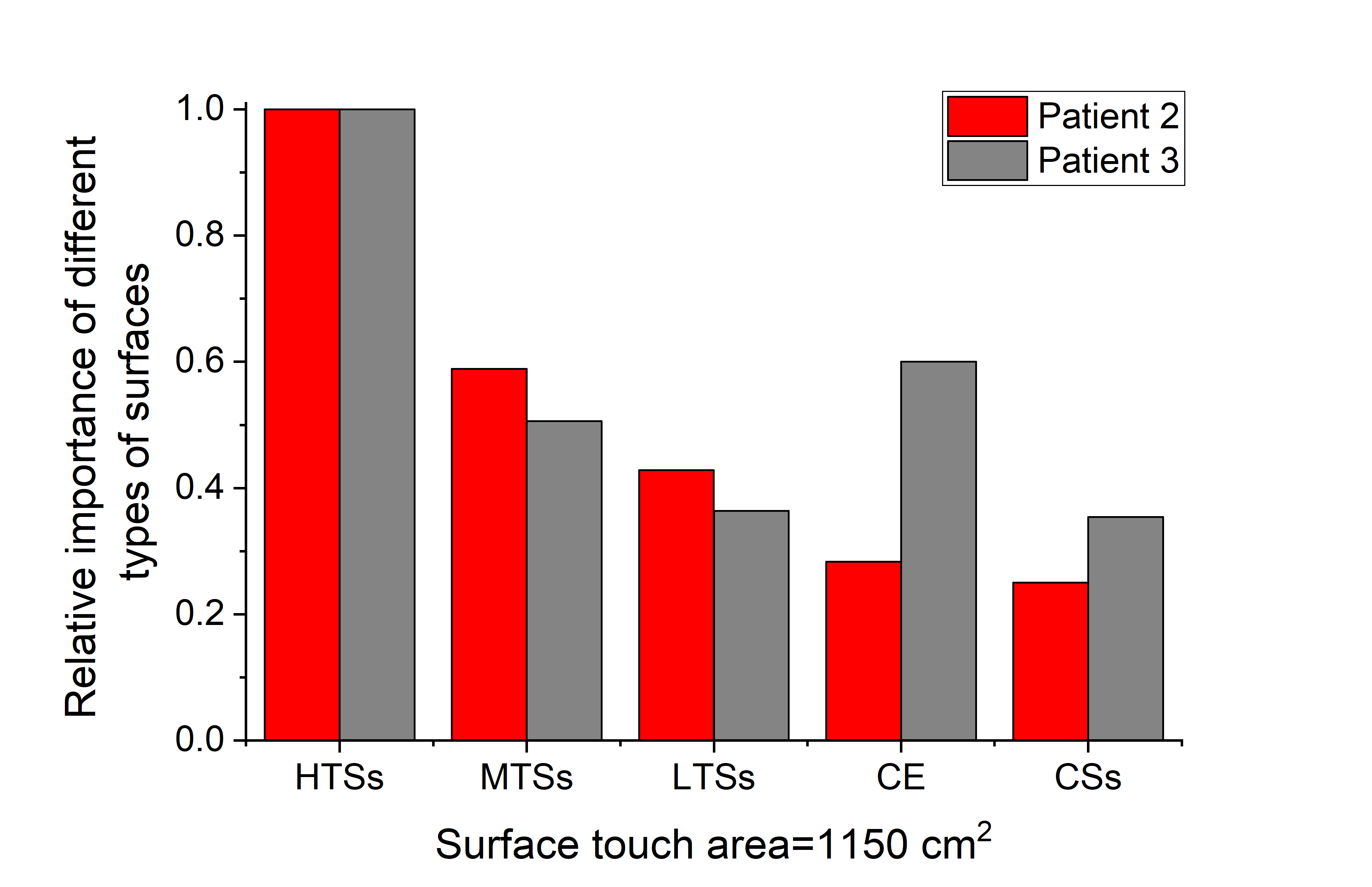

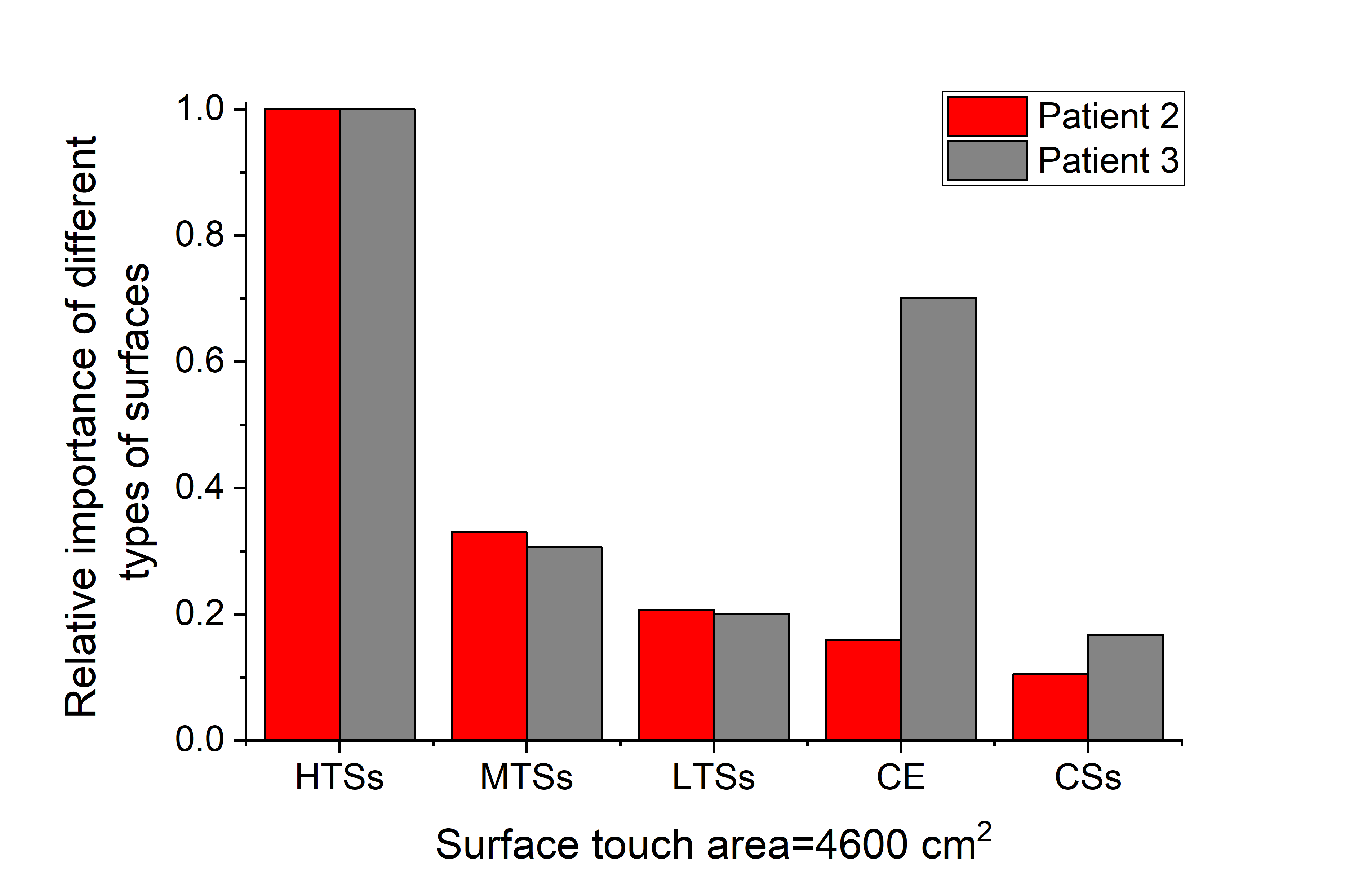


(a) (b)

**Figure S1**. Sensitivity analysis of the surface touched area, the surface touch area is assumed to be 2300 cm^2^ in the main text, (a) surface touch area is assumed to be 1150 cm^2^, (b) surface touch area is assumed to be 4600 cm^2^.


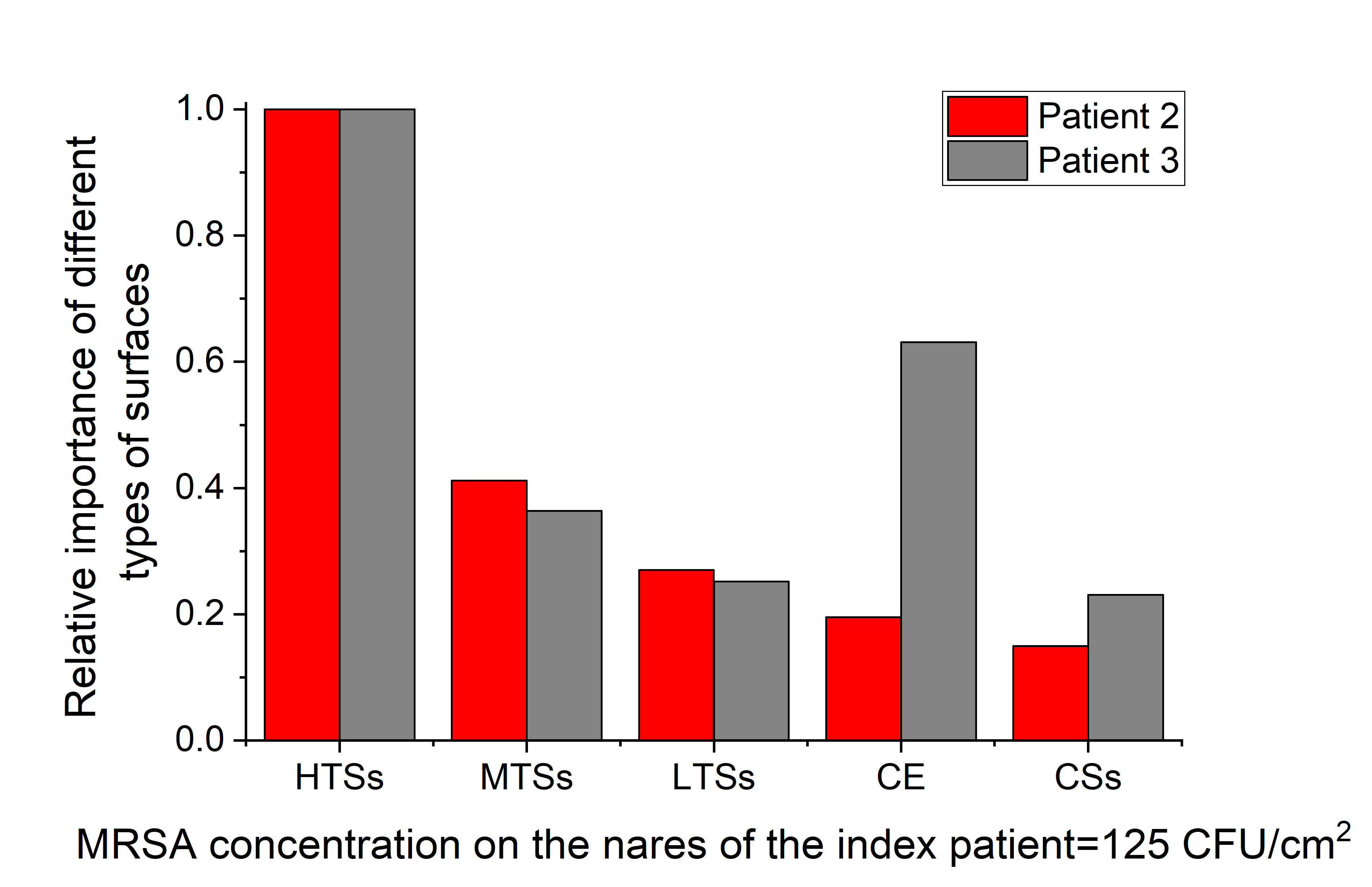

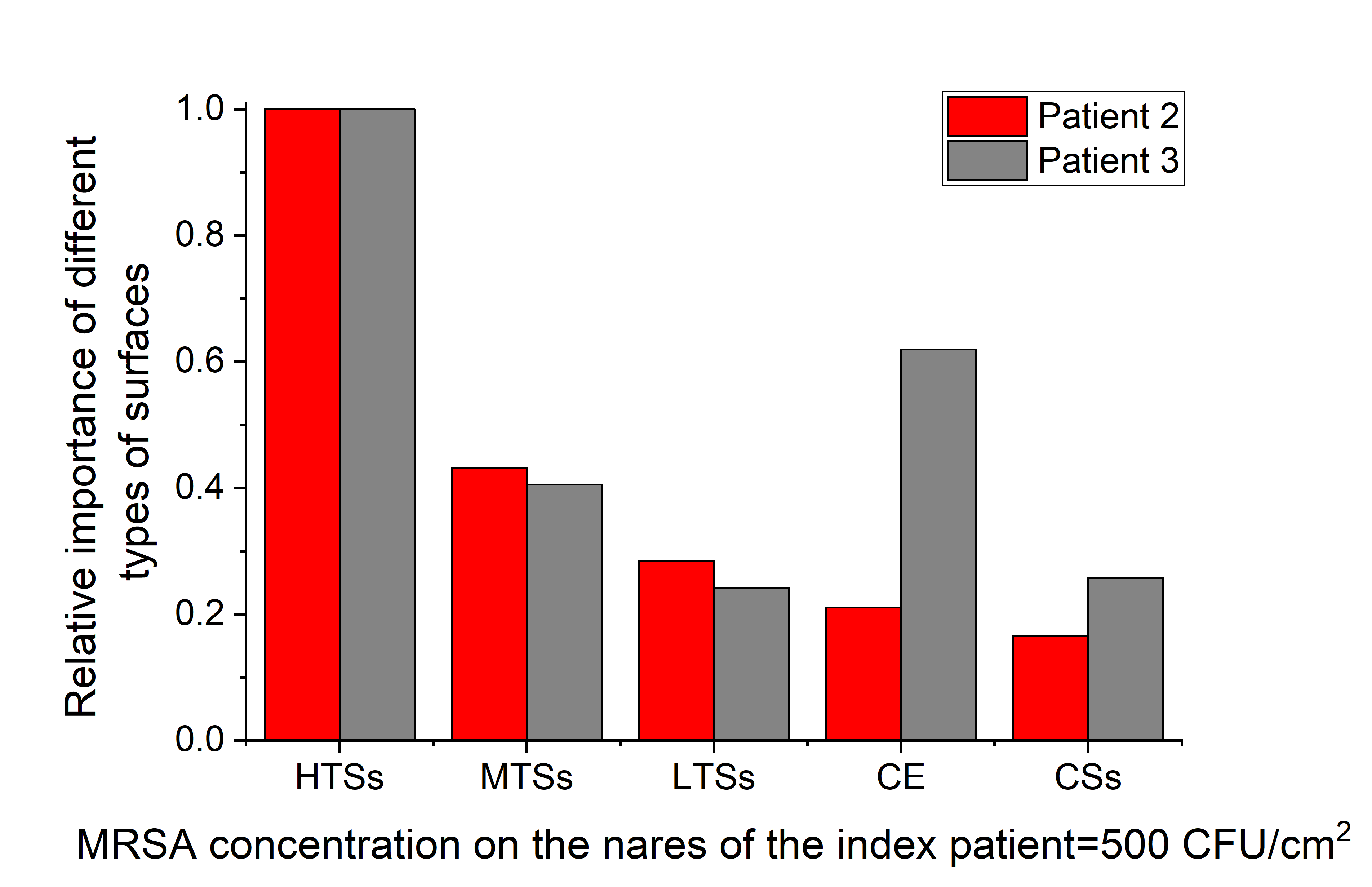


**Figure S2**. Sensitivity analysis of MRSA concentration on the nares of the index patient, the MRSA concentration on the nares of the index patient is assumed to be 250 CFU/cm^2^ in the main text, (a) MRSA concentration on the nares of the index patient is assumed to be 125 CFU/cm^2^, (a) MRSA concentration on the nares of the index patient is assumed to be 500 CFU/cm^2^.


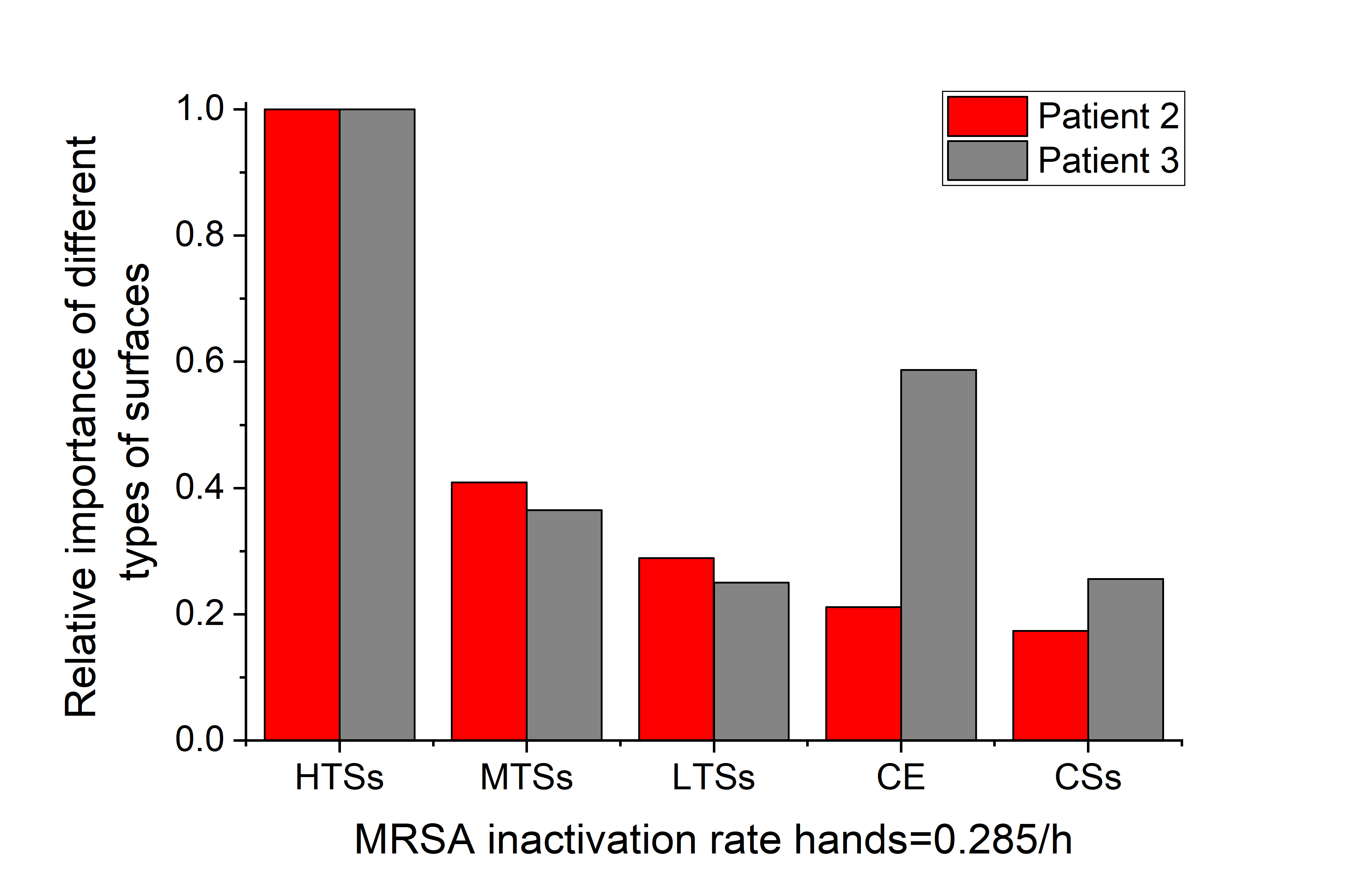

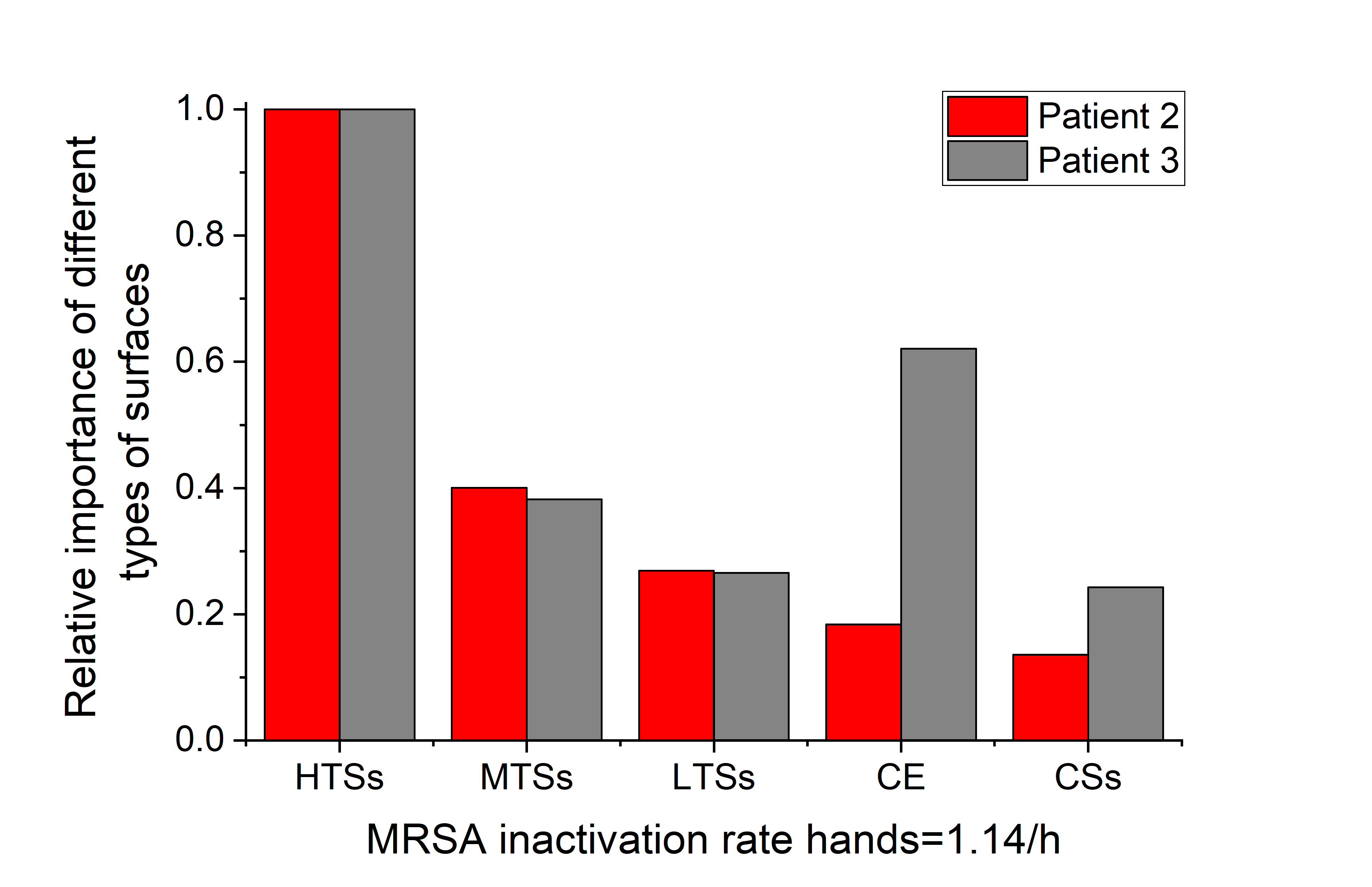


**Figure S3**. Sensitivity analysis of MRSA inactivation rate hands, the MRSA inactivation rate hands is assumed to be 0.57/h in the main text, (a) MRSA inactivation rate hands is assumed to be 0.285/h, (a) MRSA inactivation rate hands is assumed to be 1.14/h.


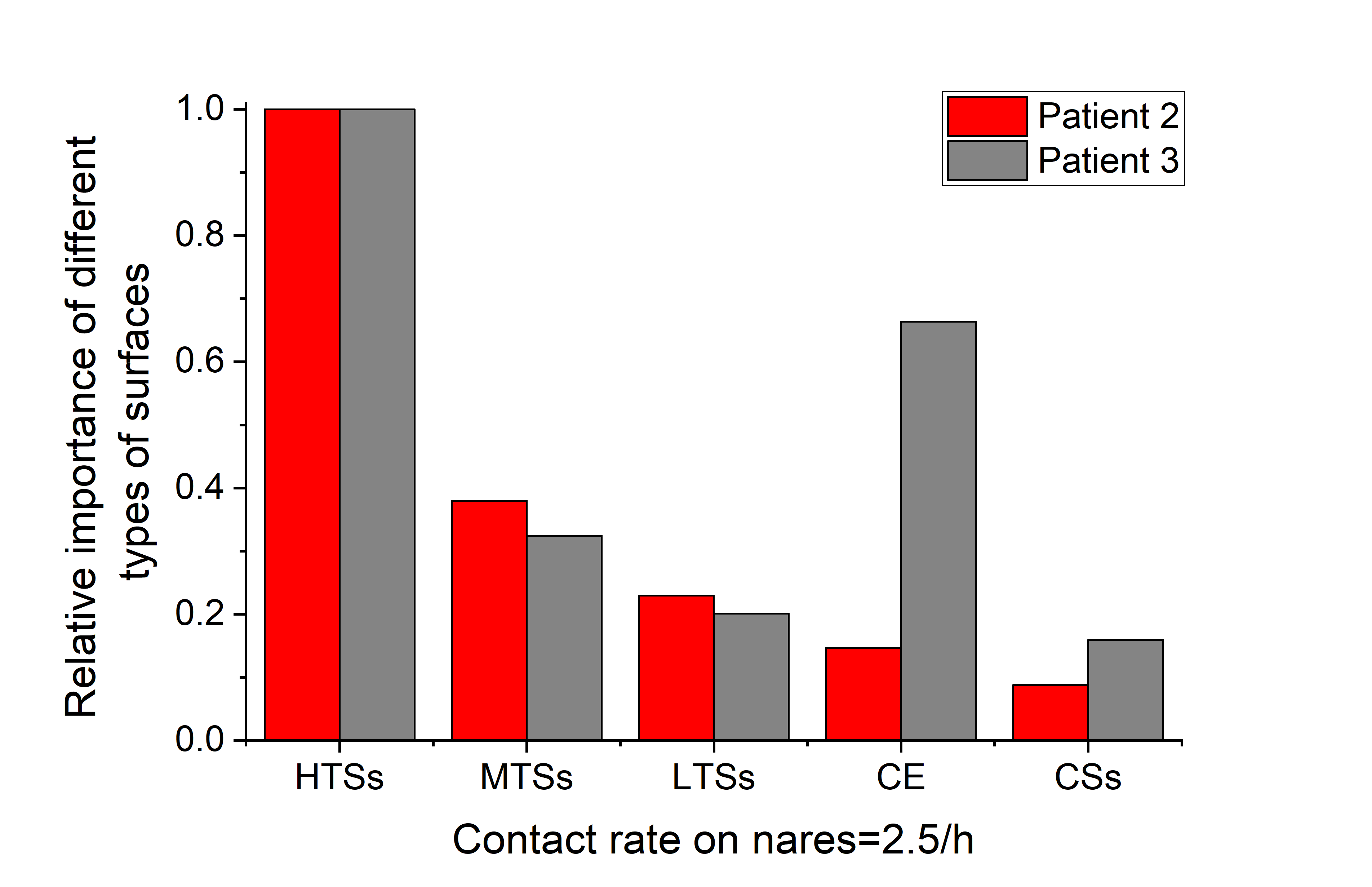


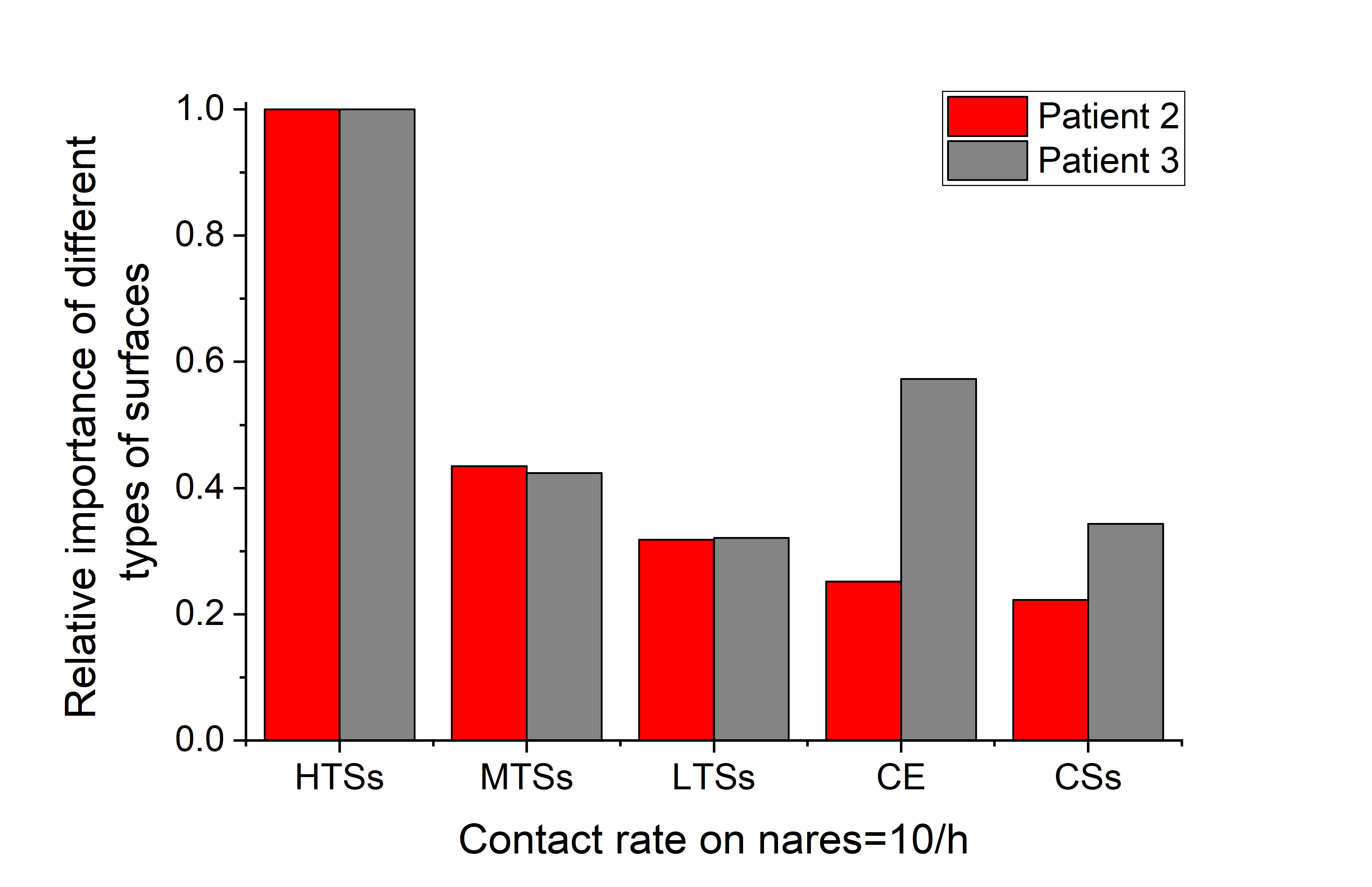


**Figure S4**. Sensitivity analysis of contact rate on nares, the contact rate on nares is assumed to be 5/h in the main text, (a) the contact rate on nares is assumed to be 2.5/h, (a) the contact rate on nares is assumed to be 10/h.

**Reference in the Supplementary**

1. AuYeung W, Canales RA, Leckie JO. The fraction of total hand surface area involved in young children's outdoor hand-to-object contacts. Environ Research 2008, 108(3): 294-299.
2. Ayliffe GAJ, Collins BJ, Lowbury EJL, Babb JR, Lilly HA. Ward floors and other surfaces as reservoirs of hospital infection. J Hyg Camb 1967, 65(4):515-536.
3. Cheng VCC, Chau PH, Lee WM, Ho SKY, Lee DWY, So SYC, Wong SCY, Tai JWM, Yuen KY. Hand-touch contact assessment of high-touch and mutual-touch surfaces among healthcare workers, patients, and visitors. J Hosp Infect, 2015, 90(3): 220-225.
4. Dara SI, Afessa B. Intensivist-to-bed ratio: association with outcomes in the medical ICU. Chest 2005, 128(2), 567-572
5. Desai R, Rannaraj PS, Agoplan J, Sugar CA, Liu GY, Miller LG. Survival and transmission of community-associated methicillin resistant Staphylococcus aureus from fomites. Am J Infect Control 2011, 39(3):219-225.
6. Gontijo Filho PP, Stumpf M, Cardoso CL. Survival of gram-negative and gram-positive bacteria artificially applied on the hands. J Clin Microbiol 1985, 21(4):652-653.
7. Huang R, Mehta S, Weed D. Methicillin-resistant Staphylococcus aureus survival on hospital fomites. Infect Control Hosp Epidemiol 2006, 27(11):1267-1269.
8. Huslage K, Rutala WA, Weber DJ. A quantitative approach to defining “high-touch” surfaces in hospitals. Infect Control Hosp Epidemio 2010, 31(8): 850-853.
9. Lee JY, Choi JW, Kim H. Determination of hand surface area by sex and body shape using alginate. J Physiol Anthropol 2007, 26(4): 475-483.
10. Lopez GU. Transfer of microorganisms from fomites to hands and risk assessment of contaminated and disinfected surfaces. PhD thesis. 2013, The University of Arizona.
11. Lopez GU, Gerba CP, Tamimi AH, Kitajima M, Maxwell SL, Rose JB. Transfer efficiency of bacteria and viruses from porous and nonporous fomites to fingers under different relative humidity conditions. Appl Environ Microbiol 2013, 79(18):5728-5734.
12. McArdle FI, Lee RJ, Gibb AP, Walsh TS. How much time is needed for hand hygiene in intensive care? A prospective trained observer study of rates of contact between healthcare workers and intensive care patients. J Hosp Infect 2006, 62(3):304-310.
13. Lopez GU. Transfer of microorganisms from fomites to hands and risk assessment of contaminated and disinfected surfaces. PhD thesis. 2013, The University of Arizona.
14. Lopez GU, Gerba CP, Tamimi AH, Kitajima M, Maxwell SL, Rose JB. Transfer efficiency of bacteria and viruses from porous and nonporous fomites to fingers under different relative humidity conditions. Appl Environ Microbiol 2013, 79(18):5728-5734.
15. McArdle FI, Lee RJ, Gibb AP, Walsh TS. How much time is needed for hand hygiene in intensive care? A prospective trained observer study of rates of contact between healthcare workers and intensive care patients. J Hosp Infect 2006, 62(3):304-310.
16. Oie S, Kamiya A. Survival of methicillin-resistant Staphylococcus aureus (MRSA) on naturally contaminated dry mops. J Hosp Infect 1996, 34(2):145-149.
17. Perez-Rodriguez F, Posada-Izquierdo GD, Valero A, Garcia-Gimeno RM, Zurera G. Modeling survival kinetics of Staphylococcus aureus and Escherichia coli O157:H7 on stainless steel surfaces solid with different substrates under static conditions of temperature and relative humidity. Food Microbiol 2013, 33(2):197-204.
18. Petti S, De Giusti M, Moroni C, Polimeni A. Long-term survival curve of methicillin-resistant Staphylococcus aureus on clinical contact surfaces in natural-like conditions. Am J Infect Control 2012, 40(10):1010-1012
19. Temime L, Opatowski L, Pannet Y, Brun-Buisson C, Boëlle, PY, Guillemot D.. Peripatetic health-care workers as potential superspreaders. PNAS 2009, 106(43), 18420-18425.
20. Preston GA, Larson EL, Stamm W. The effect of private isolation rooms on patient care practices, colonization and infection in an intensive care unit. Am J Med 1981, 70(3):641-645.
21. Albert RK, Condie F. Hand-washing patterns in medical intensive- care units. N Engl J Med 1981, 304(24):1465-1466.
22. Donowitz LG. Handwashing technique in a pediatric intensive care unit. Am J Dis Child 1987, 141(6):683-685.
23. Graham M. Frequency and duration of handwashing in an intensive care unit. Am J Infect Control 1990,18(2):77-81.
24. Dubbert PM, Dolce J, Richter W, Miller M, Chapman S. Increasing ICU staff handwashing: effects of education and group feedback. Infect Control Hosp Epidemiol 1990, 11(4):191-193.
25. Pettinger A, Nettleman M. Epidemiology of isolation precautions. Infect Control Hosp Epidemiol 1991, 12(5):303-307.
26. Larson EL, McGinley KJ, Foglia A, Leyden JJ, Boland N, Larson J, Altobelli LC, Salazar-Lindo E. Handwashing practices and resistance and density of bacterial hand flora on two pediatric units in Lima, Peru. Am J Infect Control 1992, 20(2):65-72.
27. Doebbeling BN, Stanley GL, Sheetz CT, Pfaller MA, Houston AK, Annis L, Li N, Wenzei RP. Comparative efficacy of alternative hand-washing agents in reducing nosocomial infections in intensive care units. N Engl J Med 1992, 327(2):88-93.
28. Zimakoff J, Kjelsberg AB, Larsen SO, Holstein B. A multicenter questionnaire investigation of attitudes toward hand hygiene, assessed by the staff in fifteen hospitals in Denmark and Norway. Am J Infect Control 1992;20:58-64.
29. Pittet D, Mourouga P, Perneger TV, members of the Infection Control Program. Compliance with handwashing in a teaching hospital. Ann Intern Med 1999, 130(2):126-130.
30. Desai R, Rannaraj PS, Agoplan J, Sugar CA, Liu GY, Miller LG. Survival and transmission of community-associated methicillin resistant Staphylococcus aureus from fomites. Am J Infect Control 2011, 39(3):219-225.
31. Gehanno JF, Louvel A, Nouvellon M, Caillard JF, Pestel-Caron M. Aerial dispersal of methicillin-resistant Staphylococcus aureus in hospital rooms by infected or colonized patients. J Hosp Infect 2009, 71:256-262.
32. Gontijo Filho PP, Stumpf M, Cardoso CL. Survival of gram-negative and gram-positive bacteria artificially applied on the hands. J Clin Microbiol 1985, 21(4):652-653.
33. Hambraeus A. Dispersal and transfer of Staphylococcus aureus in an isolation ward for burned patients. J Hyg Camb 1973, 71:787-797.
34. Hayden MK, Bonten MJM, Blom DW, Lyle EA, van de Vijver DA, Weinstein RA. Reduction in acquisition of vancomycin-resistant enterococcus after enforcement of routine environmental cleaning measures. Clin Infect Dis 2006, 42(11):1552-1560.
35. Huang R, Mehta S, Weed D. Methicillin-resistant Staphylococcus aureus survival on hospital fomites. Infect Control Hosp Epidemiol 2006, 27(11):1267-1269.
36. Oie S, Kamiya A. Survival of methicillin-resistant Staphylococcus aureus (MRSA) on naturally contaminated dry mops. J Hosp Infect 1996, 34(2):145-149.
37. Perez-Rodriguez F, Posada-Izquierdo GD, Valero A, Garcia-Gimeno RM, Zurera G. Modeling survival kinetics of Staphylococcus aureus and Escherichia coli O157:H7 on stainless steel surfaces solid with different substrates under static conditions of temperature and relative humidity. Food Microbiol 2013, 33(2):197-204.
38. Pittet D. Compliance with hand disinfection and its impact on hospital-acquired infections. J Hosp Infect 2001, 48: 40-46.
